# Supplementary material for: FOXP3 expression diversifies the metabolic capacity and enhances the efficacy of CD8 T cells in adoptive immunotherapy of melanoma
Source: Mol Ther. 2022 Aug 31;31(1):48–65. doi: 10.1016/j.ymthe.2022.08.017 (PMC9840123; doi:10.1016/j.ymthe.2022.08.017)
Supplement: Document S1. Figures S1–S9, Tables S1 and S2, and supplementary methods [file mmc1.pdf]

YMTHE, Volume *31*

Supplemental Information

FOXP3 expression diversifies the metabolic capacity and enhances the efficacy of CD8 T cells in adoptive immunotherapy of melanoma

Enrique Conde, Noelia Casares, Uxua Mancheño, Edurne Elizalde, Enric Vercher, Roberto Capozzi, Eva Santamaria, Juan R. Rodriguez-Madoz, Felipe Prosper, Juan J. Lasarte, Teresa Lozano, and Sandra Hervas-Stubbs

SUPPLEMENTARY DATA

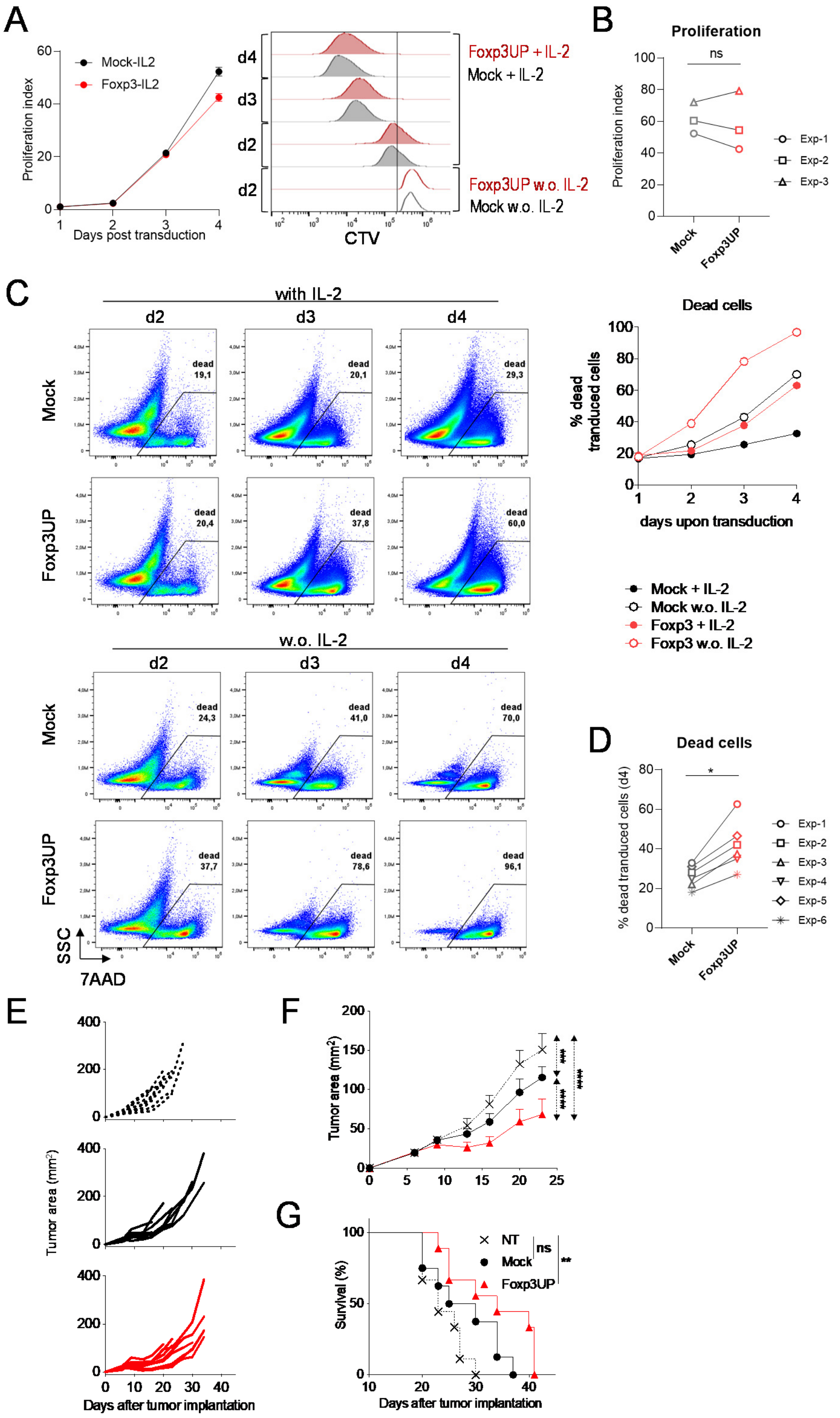

Figure S1. Effects of FOXP3 overexpression in CD8 T cells. (**A and B**) Proliferation rate of live transduced cells measured by CTV dilution assay. Magnetically sorted untouched $CD8^+$ splenocytes were activated and 48 hours later they were labeled with CTV dye and infected with an empty RV or an RV encoding FOXP3 to generate Mock and Foxp3UP CD8 T cells, respectively. After transduction, cells were cultured with (+) or without (w.o.) IL-2 for 4 days. Cells were harvested and stained with anti-CD90.1 (transduced cell surrogate marker) mAb and 7AAD to assess proliferation. (**A**) The graph shows the proliferation index calculated as "Median Fluorescence Intensity (MFI) of cells cultured w.o. IL-2 corresponding to day 1 (average of replicates) / CTV of cells expanded with IL-2 at the day indicated". (Right) Overlay histograms showing CTV MFI of representative wells of Mock and Foxp3UP CD8 T cells expanded with or without IL2 at the days indicated. (**B**) Proliferation index of cells cultured with IL-2 (day 4 post infection). Compiled data from 3 independent experiments. (**C**) Percentage of dead ($7AAD^+$) cells within transduced ($CD90.1^+$) cells along the culture period. Dot plots show representative wells of each culture condition at the days indicated. (**D**) Percentage of dead cells within transduced cells at day 4 of culture. Compiled data from 6 independent experiments. (**E-G**) Six-day B16F10 tumor-bearing mice were treated with 7-day in vitro-expanded Foxp3UP or Mock Pmel cells (10^7/mouse; 7 mice/group). Tumor size (mm^2) from individual mice (**E**), average tumor size (**F**) and overall survival (**G**). Data are represented as mean±SD (A and C) and mean±SEM (F). Symbols represent individual mice (**F**) or experiments (**B and D**). Statistical significance was determined using paired t test (**B and D**), nonlinear regression (curve fit) (**F**), and Mantel-Cox test (**G**). ***$p < 0.0005$, **$p < 0.005$, *$p < 0.05$. One experiment representative of 6 (**C**), 3 (**A**) and 2 (**E-G**) is shown.

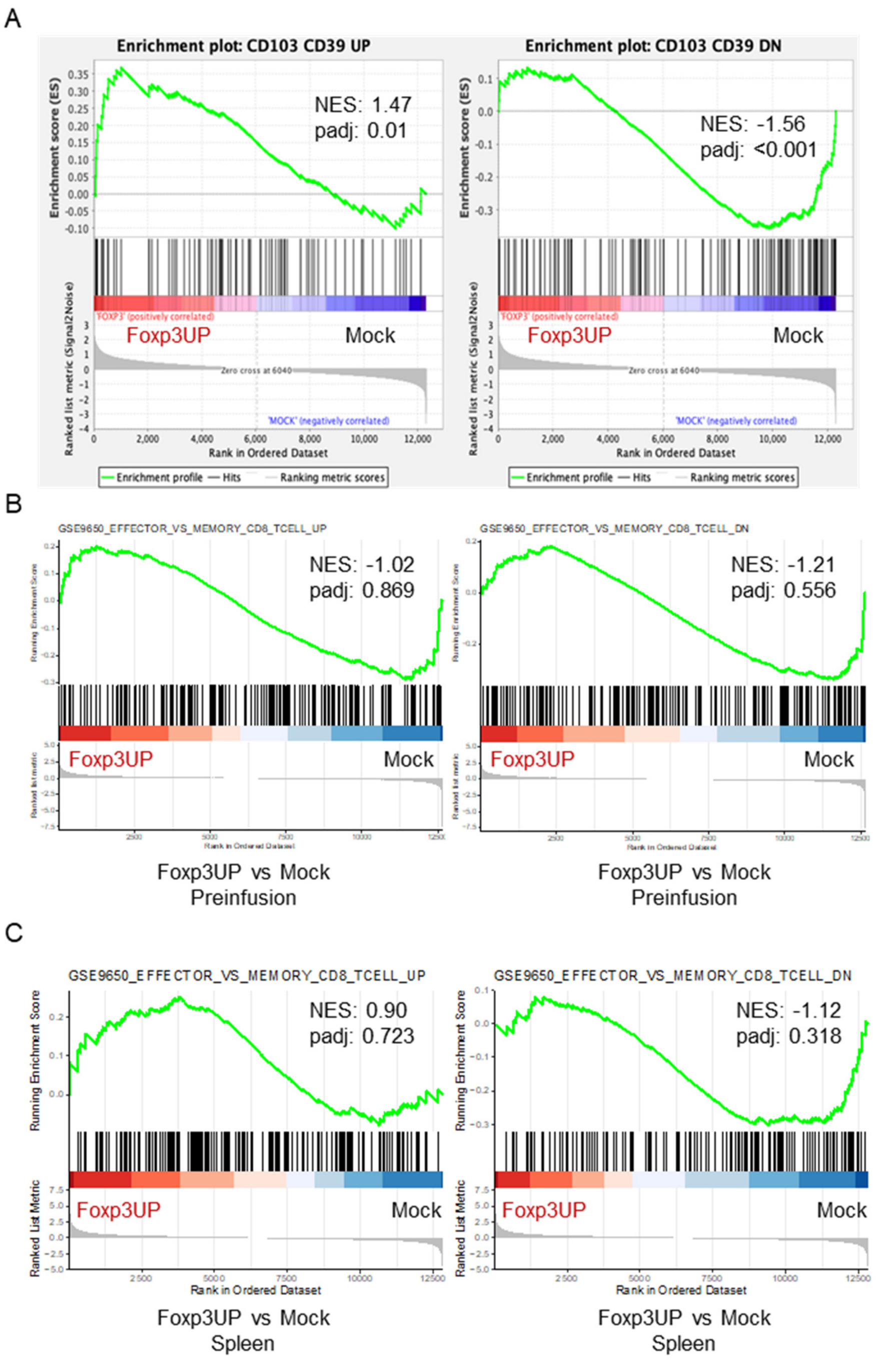

Figure S2. **Transcriptomic signatures of tumor-infiltrating Foxp3UP CD8 T cells** (**A**) GSEA of CD103^{+}CD39^{+} cell gene sets [upregulated (UP) and downregulated (DN) genes] obtained from GSE114944 in the Foxp3UP vs Mock TIL dataset. (**B and C**) In parallel to experiments shown in figure 2, RNAseq was performed in Foxp3UP and Mock OT-I cells prior to infusion (preinfusion) (**B**) and in those infiltrating the spleen after ACT (**C**). GSEA enrichment score curve of "Effector vs memory CD8+ " upregulated (UP) and downregulated (DN) gene sets in the transcriptome of Fox3UP vs Mock CD8 T cells presented as the normalized enrichment score (NES). (**A-C**) Compiled data from 4 different experiments.

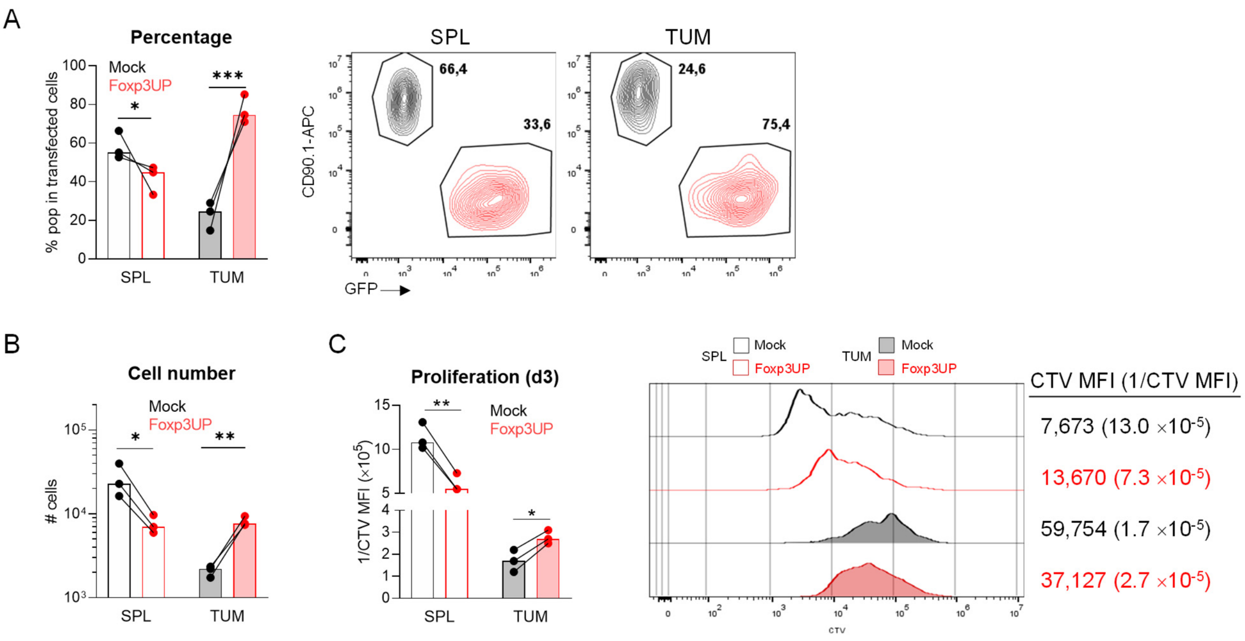

Figure S3. FOXP3 overexpression enhanced CD8 T cell proliferation in the TME. Second experiment representative of the one shown in figure 4A-E. OT-I cells were analyzed in tumor and spleen at day 3. (**A**) Graph (left) and representative dot plots show the percentage of Foxp3UP and Mock OT-I cells in total transduced (GPF^{+} plus CD90.1^{+}) CD45.1^{+} cells in tumor (TUM) and the spleen (SPL). (**B**) Total number of Foxp3UP and Mock CD8 T cells in tumor and the spleen. (**C**) (Left) Proliferation [depicted as reverse of CTV MFI (1/CTV MFI)] of Foxp3UP and Mock OT-I cells in tumor and spleen. (Right) Representative histogram. In this experiment, the number of Foxp3UP and Mock CD8 T cells per mg of tumor was not determined because tumor weight was not recorded. Data are represented as mean. Symbols represent individual mice. Statistical significance was determined using paired *t* test ***p < 0.0005, **p < 0.005, *p < 0.05.

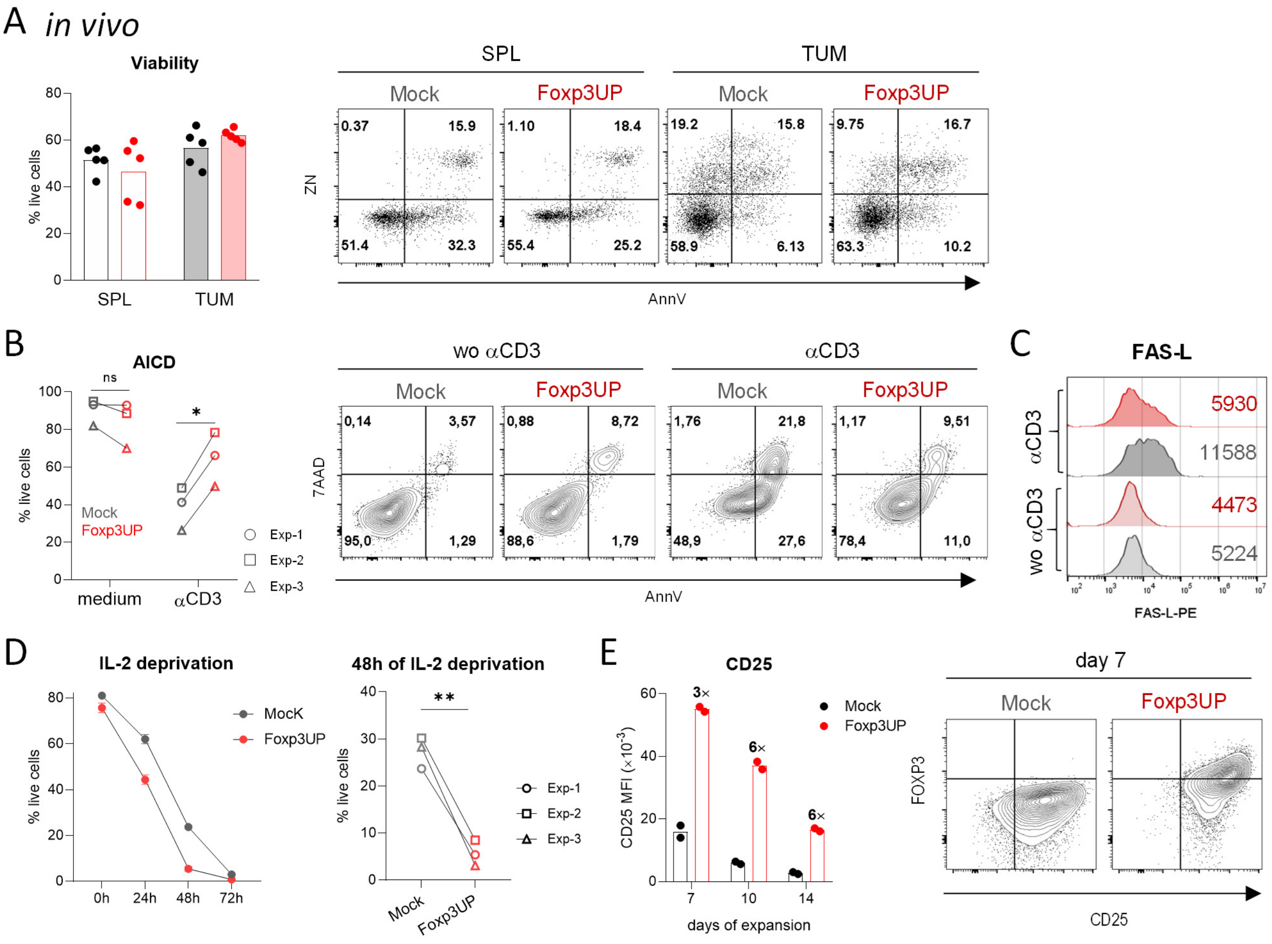

Figure S4. Effect of FOXP3 overexpression on CD8 T cell sensitivity to AICD and IL-2 deprivation. (A) *In vivo* viability of transferred cells. Since GFP expression is lost when cells die, *in vivo* viability assessment was performed using CD90.1 as the transduced cell surrogate marker. Foxp3UP (CD90.1$^+$) and Mock (CD90.1$^+$) OT1 (CD45.1$^+$) cells were separately injected into 10-day B16OVA tumor-bearing BL6 (CD45.2$^+$) mice. Five days later, cells from tumors and spleens were stained with Zombie NIR (ZN) and Annexin V (AnnV) and viability [% of viable (ZN$^-$AnnV$^-$) cells within transduced (CD90.1$^+$CD45.1$^+$) cells] was assessed by FACS. Bar plot and representative dot plots are shown. **(B)** AICD Assay. 4-day *in vitro*-expanded Mock (CD90.1$^+$) and Foxp3UP (CD90.1$^+$) CD8 T cells cleaned from dead cells were cultured in 96-well plates coated with or without (resting) anti-CD3 mAb (1µg/ml) in the absence of IL-2. After 24 hours of culture, samples were stained with 7AAD and AnnV and analyzed by FACS. (**B, left**) Percentage of live (7AAD$^-$AnnV$^-$) cells within transduced (CD90.1$^+$) cells. Compiled data from 3 independent experiments. (**B, right**) Representative dot plots from one experiment. **(C)** FASL surface expression depicted as MFI. Cells cultured as in **B** and stained with anti-FASL mAb, AnnV and 7AAD dye. Expression of FASL was analyzed in live transduced (7AAD$^-$AnnV$^-$CD90.1$^+$) cells by FACS. **(D)** IL-2 deprivation assay. Eight-day *in vitro*-expanded Mock (CD90.1$^+$) and Foxp3UP (CD90.1$^+$) CD8 T cells cleaned from dead cells were cultured in the absence of IL-2. The percentage of live (7AAD$^-$AnnV$^-$) cells of transduced (CD90.1$^+$) cells was analyzed at baseline and at different time of culture. **(D, left)** One representative experiment. (**D, right**) Percentage of live cells after 48h of IL-2 deprivation. Compiled data from 3 independent experiments. **(E)** CD25 surface expression depicted as MFI. CD8$^+$ splenocytes were activated (day 0) and 48 hours later they were transduced with Mock or FOXP3 RV. After transduction, cells were cultured with IL-2 up to day 14. Expression of CD25 was analyzed in live transduced (7AAD$^-$CD90.1$^+$) cells by FACS**.** Data are represented as mean (**A and E**), mean±SD (**D**). Symbols represent individual mice (**A**), independent experiments (**B and D left**) or experimental duplicates (**E, left**). Statistical significance was determined using two-way ANOVA for multiple comparisons (**A**) and paired t test (**B left and D right**). **p < 0.005, *p < 0.05. One experiment representative of 2 (**A and E**) and 3 (**C**) experiments is shown.

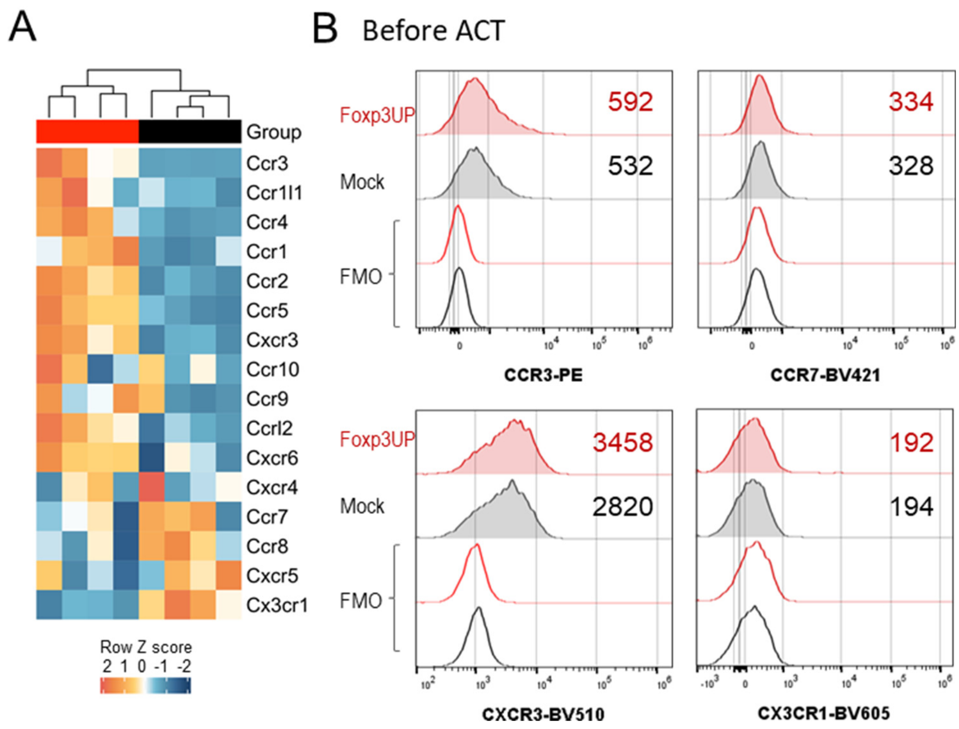

Figure S5. Effect of FOXP3 overexpression on the surface expression of chemokine receptors by CD8 T cells. (**A**) Gene expression heat map of chemokine receptors in Foxp3UP and Mock OT-I cells isolated form tumors (Data from RNAseq). (**B**) Expression of different chemokine receptors in 4-day *in vitro*-expanded Foxp3UP and Mock OT-I cells. Representative histograms are shown. As negative control, fluorescence minus one (FMO) is shown. Compiled data from 4 different experiments (**A**). One experiment representative of 2 experiments (**B**).

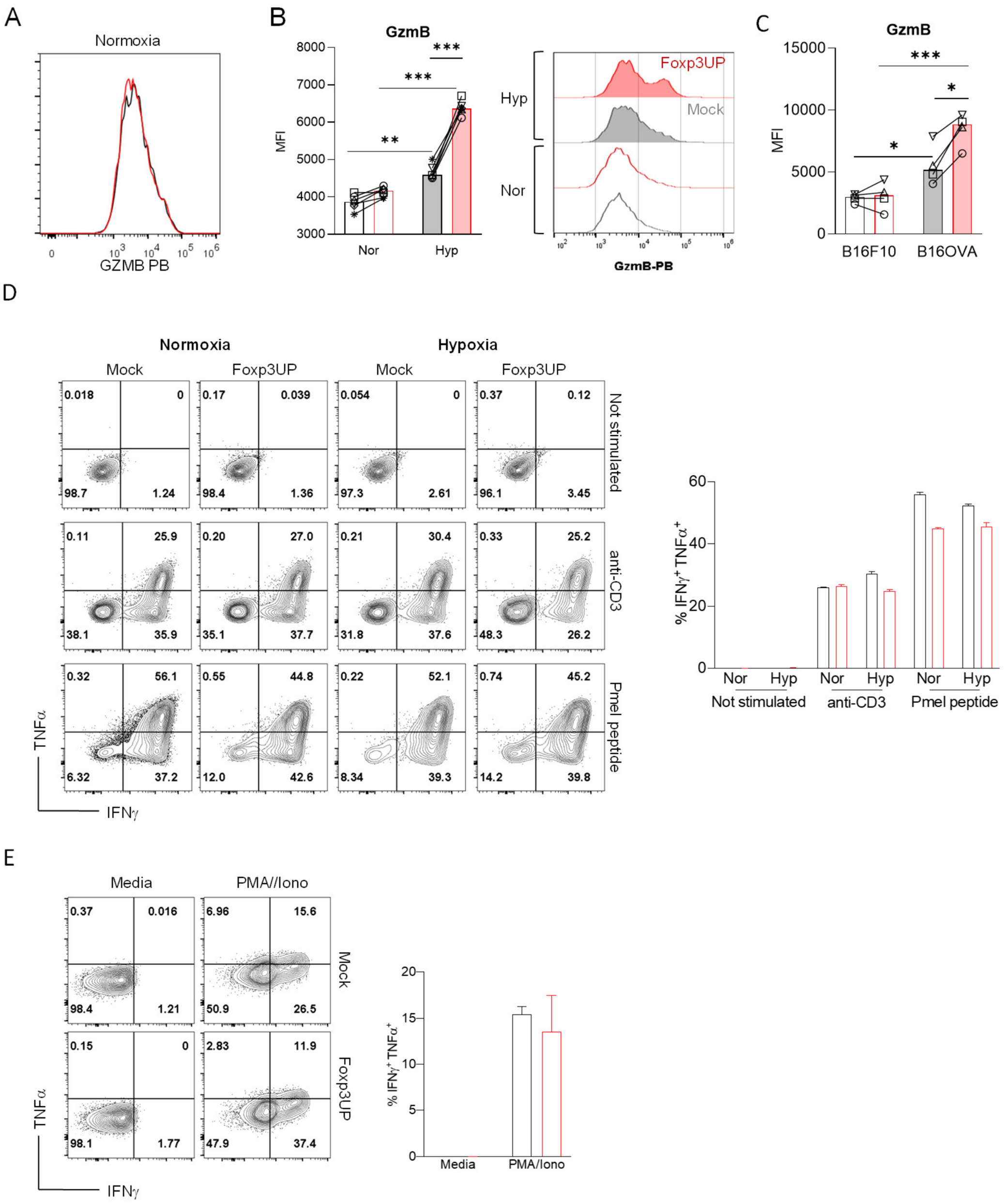

Figure S6. *In vitro*, Foxp3UP CD8 T cells exhibited enhanced granzyme B (GzmB) expression but diminished production of effector cytokines. (**A**) Overlay histograms showing GzmB MFI in Mock and Foxp3UP CD8 T cells before transfer. (**B and C**) Effect of hypoxia and tumor cells on the GzmB expression (depicted as MFI) in T cells. (**B**) Activated Mock (GFP^+) and Foxp3UP (GFP^+) OT-I cells were cultured *in vitro* for 48 hours under normoxic (20% O_2) or hypoxic (1% O_2) conditions. Graph shows compiled data from 6 different experiments. (**C**) OT-1 T cells were cultured (18 hours) with cognate (B16OVA) or non-cognate (B16F10) tumor cells at a T cell:tumor cell ratio of 5:1. Graph shows compiled data from 3 different experiments. (**D and E**) Cells were cultured in normoxic (**D and E**) or hypoxic (**D**) conditions as in **B**. At 48 hours of culture, OT-I cells were restimulated with OVA peptide (**D**) or PMA/Ionomycin (PMA/Iono) (**E**) and Brefeldin A. IFNγ and TNFα production was assessed 4 hours later by FACS. Representative FACS dot plots are depicted (**D and E**). (**D and E, right**) Percentage of $IFN\gamma^+TNF\alpha^+$ cells within Mock and Foxp3UP CD8 T cells. Data are represented as mean (**B and C**) and mean±SD (**D and E**). Symbols represent independent experiments (**B and C**). Statistical significance was determined using two-way ANOVA for multiple comparisons (**B and C**). ***$p < 0.0005$, **$p < 0.005$, *$p < 0.05$. One experiment representative of 2 (**D and E**), 4 (**A**) or 6 (**B, right**) experiments is shown.

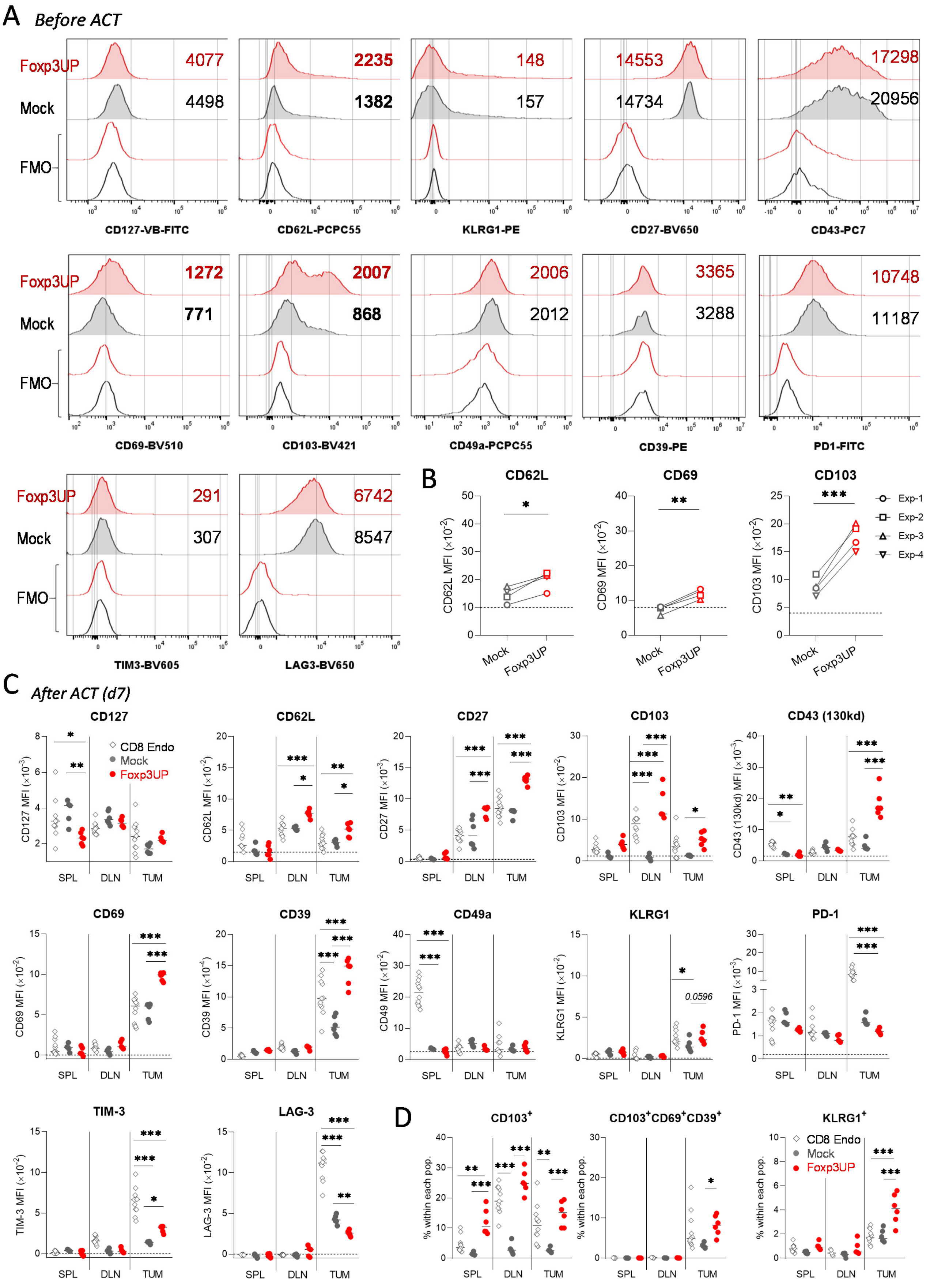

Figure S7. Continued on next page

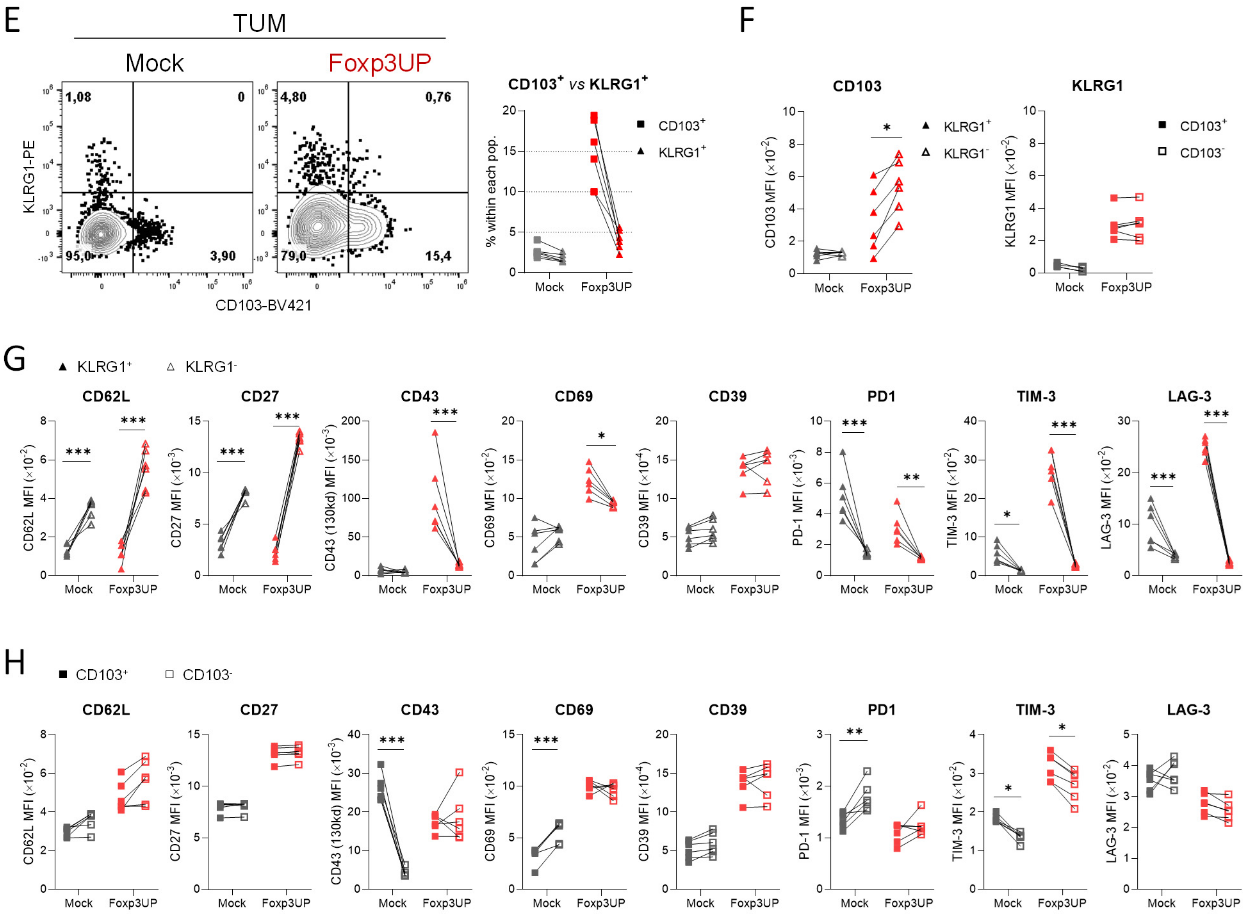

Figure S7. Effect of FOXP3 overexpression on the expression of different markers associated with differentiation, activation and exhaustion of CD8 T cells. (**A and B**) Expression of different surface markers in 4-day *in vitro*-expanded Foxp3UP and Mock OT-I cells. (**A**) Representative histograms. As negative control, FMO is shown. (**B**) MFI of CD62L, CD69 and CD103 in Foxp3UP and Mock CD8 T cells. Compiled data from 4 independent experiments. Dotted line indicates FMO values. (**C-H**) 4-day *in vitro*-expanded Foxp3UP and Mock OT1 cells were injected separately into 10-day B16OVA tumor-bearing BL6 mice (n = 6). The expression of different surface markers was assessed (FACS) in Foxp3UP and Mock CD8 T cells from spleen, dLN and tumors at day 7 of ACT. (**C**) MFI of assessed markers in Foxp3UP and Mock CD8 T cells. Dotted line indicates FMO values. (**D**) Percentage of $CD103^+$, $CD103^+CD69^+CD39^+$ and $KLRG1^+$ within each cell subset inndicated. (**C and D**) As reference, endogenous (endo) CD8 T cells are shown. (**E**) Percentage of $CD103^+$ cells vs $KLRG1^+$ cells within Mock and Foxp3UP CD8 TILs. (**F**) MFI of CD103 and KLRG1 marker on $KLRG1^+$ *vs* $KLRG1^-$ cells (left) and $CD103^+$ *vs* $CD103^-$ cells (right), respectively, within Foxp3UP and Mock OT-I TILs. (**G and H**) MFI of indicated markers in $KLRG1^+$ *vs* $KLRG1^-$ cells (**G**) and $CD103^+$ *vs* $CD103^-$ cells (**H**) within Foxp3UP and Mock OT-I TILs. Data are represented as median (**C**). Symbols represent independent experiments (**B**) or individual mice (**C-H**). Statistical significance was determined using paired *t* tests (**B**) and two-way ANOVA for multiple comparisons (**C-H**). (**C-H**) Only significances between relevant groups are shown. ***p < 0.0005, **p < 0.005, *p < 0.05. One experiment representative of 2 is shown (**A and C-H**).

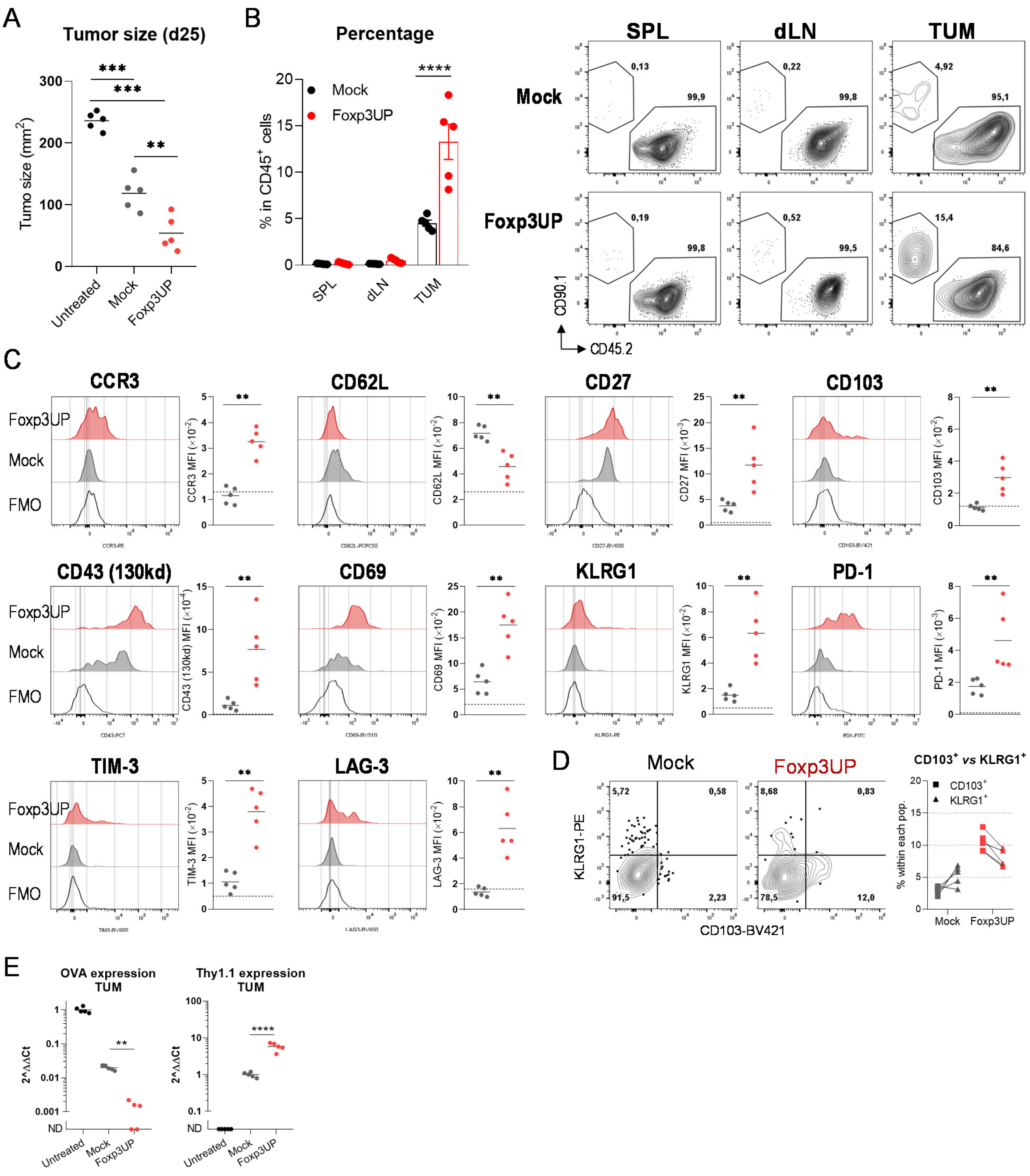

Figure S8. Foxp3UP CD8 T cells outnumbered Mock cells in tumors and exhibited a greater ability to control tumor growth in the late-phase of ACT at the expense of becoming exhausted. Foxp3UP (CD90.1^{+}) and Mock (CD90.1^{+}) OT1 (CD45.1^{+}) cells were separately injected into 10-day B16OVA-bearing BL6 (CD45.2^{+}) mice (10^{6}/mouse; 5 mice/group). As control, one group of mice was left untreated. Twenty-five days later, cells from spleens, dLNs and tumors were analyzed by FACS. (**A**) Tumor size (mm^{2}). (**B**) Percentage of transduced (CD90.1^{+}) cells within CD45^{+} cells in treated mice. (Right) Representative dot plots. CD45.2^{+} cells are endogenous cells. (**C**) The expression of different surface markers was assessed in Foxp3UP and Mock OT-I TILs. Representative histograms (left) and graphs (right) showing the MFIs for each marker. As negative control, FMO corresponding to Foxp3UP cells is included (empty grey histogram in the overlaid histograms and dotted line in the graphs). (**D**) OVA and Thy1.1 gene expression determined by quantitative RT-PCR using RNA isolated from total tumor. ND (not detected). Data are represented as mean (**A, C**-D) and mean+SEM (**B**). Symbols represent individual mice (**A-D**). Statistical significance was determined using unpaired *t* test (**C and D**) and two-way ANOVA for multiple comparisons (**A and B**). (**B**) Only significances between relevant groups are shown. ****p < 0.0001, ***p < 0.0005, **p < 0.005. One experiment representative of 2 experiments.

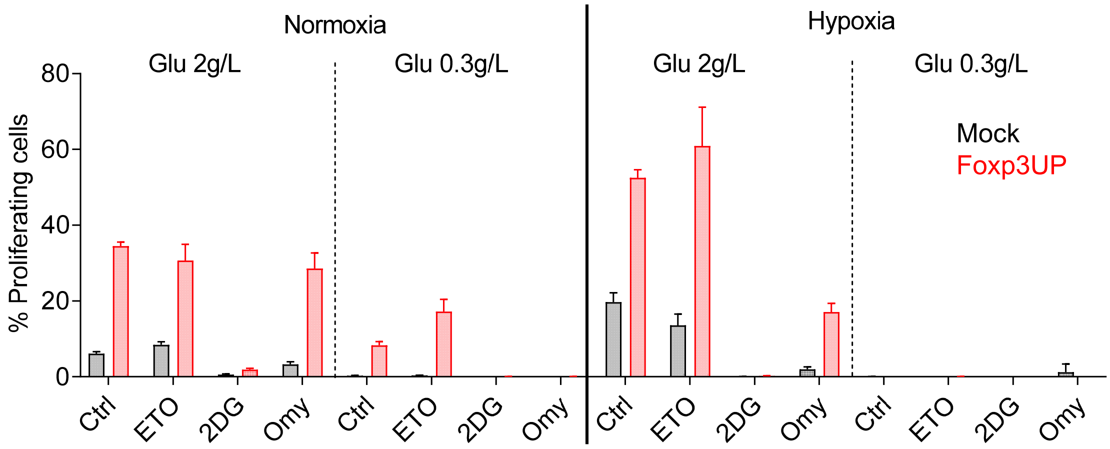

Figure S9. Proliferation of CD8 T cells in medium supplemented with low-glucose and dialyzed serum in the presence/absence or metabolic inhibitor under normoxic and hypoxic conditions. 8-day *in vitro*-activated Foxp3UP and Mock CD8 T cells (both GFP^+) were labeled with CTV dye and re-stimulated *in vitro* (72 hours) with plate-bound anti-CD3 mAb in glucose-free DMEM medium supplemented with normal or low glucose concentration (2 and 0.3 g/L, respectively), dialyzed fetal calf serum (FCS) in the presence or absence of 2-DG (1 mM), Eto (50 µM) or Oligo (3 nM), under normoxia (20% O_2) or hypoxia (1% O_2). The percentage of proliferating cells within transduced (GFP^+) cells is shown. Data are represented as mean±SD.

Table S1. GSEA of the comparison of Foxp3UP/Mock CD8 T cells before and after ACT in the spleen and tumor. GSEA by clusterProfiler illustrates gene sets significantly enriched in Foxp3UP/Mock cell comparison before and after ACT in the spleen and tumor. NES and padj in Foxp3UP/Mock cell comparison are shown. Those NES with a significant padj (padj < 0.05) are shown in bold. NA: not available

	BEFORE ACT		AFTER ACT			
			SPLEEN		TUMOR	
**Description**	**NES**	**padj**	**NES**	**padj**	**NES**	**padj**
Long-chain fatty acid metabolism	1.4198	*0.8117*	-1.0749	*0.8351*	**2.0133**	***0.0208***
Cell adhesion molecules	1.3152	*0.6769*	1.1161	*0.8326*	**1.9713**	***0.0035***
Tolerance induction	1.1341	*0.8744*	1.3966	*0.6984*	**1.9658**	***0.0269***
Oxidative phosphorylation	NA	*NA*	1.1615	*0.7702*	**1.8406**	***0.0042***
Leukocyte migration	1.1451	*0.8242*	1.1283	*0.7551*	**1.7568**	***0.0160***
cAMP-mediated signaling	1.2633	*0.8117*	-1.3747	*0.6781*	**1.7469**	***0.0466***
Cell chemotaxis	-1.2244	*0.8117*	-1.4005	*0.3519*	**1.6538**	***0.0266***
T cell proliferation	-1.1409	*0.8327*	0.9728	*0.8683*	**1.6456**	***0.0287***
Focal adhesion-PI3K-AKT-MTOR signaling pathway	0.9251	*0.8684*	NA	*NA*	**1.5786**	***0.0076***
Hypoxia	0.9164	*0.8079*	1.1854	*0.5327*	**1.4939**	***0.0117***
Adipogenesis	1.2129	*0.3981*	-1.0692	*0.6584*	**1.3900**	***0.0269***
Glycolysis	NA	*NA*	0.9410	*0.7427*	**1.3533**	***0.0361***
MTORC1 signaling	-1.2514	*0.3413*	-0.9966	*0.9140*	**-1.2802**	***0.0349***
WNT-beta-catenin signaling	-1.0957	*0.5629*	1.0317	*0.7083*	**-1.5268**	***0.0349***
TNFa signaling via NF-κB	1.0688	*0.5601*	-1.3841	*0.2889*	**-1.6707**	***0.0014***
Signaling pathways regulating pluripotency of stem cells	-1.1068	*0.7972*	0.8970	*0.9521*	**-2.2534**	***0.0042***
Ribosome biogenesis in eukaryotes	-1.0595	*0.8349*	-1.6486	*0.1661*	**-2.7562**	***0.0042***
MYC targets (V2)	-0.8982	*0.8037*	-1.3864	*0.3433*	**-2.9127**	***0.0009***

Table S2. Distinctive phenotypes of genetically modified CD8 T cells

<table>
<tr><th rowspan="2">Engineered CD8 T cells</th><th rowspan="2">CD8 donor mouse strain</th><th colspan="2">Donor cell phenotype</th><th rowspan="2">Retroviral Vector</th><th rowspan="2">Distinctive phenotype of genetically modified CD8 T cell</th></tr>
<tr><th>CD45 allele</th><th>CD90 allele</th></tr>
<tr><td rowspan="3">Foxp3UP</td><td rowspan="2">OT-1xCD45.1</td><td rowspan="2">CD45.1</td><td rowspan="2">CD90.2</td><td>MSCV-Foxp3-IRES-Gfp</td><td>CD45.1$^+$GFP$^+$</td></tr>
<tr><td>MSCV-Foxp3-IRES-Thy1.1(#)</td><td>CD45.1$^+$CD90.1$^+$</td></tr>
<tr><td>Pmel (Thy1.1)</td><td>CD45.2</td><td>CD90.1</td><td>MSCV-Foxp3-IRES-Gfp</td><td>CD90.1$^+$GFP$^+$</td></tr>
<tr><td rowspan="3">Mock</td><td rowspan="2">OT-1xCD45.1</td><td rowspan="2">CD45.1</td><td rowspan="2">CD90.2</td><td>MSCV-IRES-Gfp</td><td>CD45.1$^+$GFP$^+$</td></tr>
<tr><td>MSCV-IRES-Thy1.1</td><td>CD45.1$^+$CD90.1$^+$</td></tr>
<tr><td>Pmel (Thy1.1)</td><td>CD45.2</td><td>CD90.1</td><td>MSCV-IRES-Gfp</td><td>CD90.1$^+$GFP$^+$</td></tr>
</table>

(#) *Thy1.1* encodes CD90.1 protein.

SUPPLEMENTARY METHODS

Plasmids

pCL-Eco was a gift from Inder Verma[1] (Addgene plasmid # 12371). MSCV-IRES-*Gfp* was a gift from Tannishtha Reya (Addgene plasmid # 20672). MSCV-IRES-*Thy1.1* DEST[2] was a gift from Anjana Rao (Addgene plasmid # 17442). Mouse *Foxp3* open reading frame (CCDS29965.1) was synthesized (GenScript) and inserted into MSCV-IRES- *Gfp* and MSCV-IRES-*Thy1.1* plasmids.

Retrovirus production

PLATE cells (6×10^5 cells/well) were seeded 24 hours before transfection into 6-well plates in 2 mL/well of PLATE medium [DMEM-Glutamax, FCS 10%, sodium pyruvate 1%, essential amino acids 1%, HEPES, 100 U/mL penicillin (P) and 100 µg/mL streptomycin (S)] without P/S. The next day, cells (70% confluence) were transfected with a mixture containing plasmids and Lipofectamine 2000 (500 µL/well). The mixtures contained 3 µg/well of transgene-carrying plasmid, 2 µg/well of pCL-Eco (helper plasmid) and 10 µL/well of Lipofectamine 2000, and were prepared in OPTIMEM medium according to the manufacturer´s protocol. PLATE medium was changed 24 hours post transfection. The supernatant containing the retroviruses (RV) was collected 48 hours and 72 hours post transfection. Debris was removed by centrifugation at 2000 rpm for 1 minute. Supernatants were kept at 4ºC until T cell transduction.

Tissue processing

Tumor-bearing mice were sacrificed and excised tumors were incubated in a dissociation solution [RPMI-1640-glutamax supplemented with 400 U/mL collagenase D (Roche) and 50 µg/mL of DNase I (Roche)] for 30 minutes at 37ºC. Tumors were mechanically homogenized, filtered through a 70 µm nylon cell strainer (FALCON) and centrifuged at 2000 rpm for 8 minutes. After erythrocyte lysis with ammonium-chloride-potassium (ACK) lysing buffer (0.15 M NH_4Cl, 1 mM $KHCO_3$ and 0.1 mM Na_2EDTA), tumor cell suspension ($10^6\times$cells/ml) was overlaid on 3 mL of Lympholyte-M (Cederlane) and centrifuged at 1400 g for 20 min at room temperature. The interface was collected and washed in phosphate buffered saline (PBS) before mAb staining and flow sorting as detailed below. In some experiments, spleens and draining lymph nodes were also harvested, homogenized by mechanical dissociation through a 70µm nylon cell strainer and treated with ACK buffer to remove erythrocytes.

Cell labelling for FACS

Different fluorophore-conjugated mAbs or proteins were used for FACS and cell sorting: antibody against mouse CD90.1 (OX-7), GFP (FM264G), FOXP3 (FJK-16s), CD8 (53-6-7), CD45 (I3/2.3), CD45.1 (A20), CD45.2 (104), CD103 (2 E7), CD69 (HI.2F3), CD39 (Duha59), FASL (MFL3), IFNγ (XMG1,2), TNFα (MP6-XT22), GLUT1 (EPR3915), CD36 (HM36), Ki-67 (16A8), CD25 (3C7), CD62L (MEL-14), CD27 (LG.3A10), CD127 (A7R34), CD49a (HMa1), KLRG1 (14C2A07), CD43 (130 kd) (1B11), PD-1 (29F-1A12), TIM-3 (RMT3-23), LAG-3 (C9B7W), CCR3 (J073E5), CCR4 (2G12), CCR7 (4B12), CXCR3 (Cxcr3-173), CX3CR1 (Z8-50), human GZMB (GB11) and Annexin V. All these reagents were fluorochrome-labeled and obtained from Biolegend. Other reagents and dyes used were: 2-NBDG [2-(N-(7-Nitrobenz-2-oxa-1,3-diazol-4-yl)Amino)-2-Deoxyglucose] (Invitrogen), Bodipy-493 (Thermofisher), Bodipy-FL-C16 (Thermofisher), Zombie NIR Fixable dye (Biolegend), SYTOX Blue Dead Cell Stain (Thermofisher), 7-aminoactinomycin D (7AAD) (Thermofisher) and cell trace violet (CTV) (Invitrogen).

For analysis of surface markers, cells were incubated with Zombie NIR Fixable dye and subsequently with a mix of fluorophore-conjugated mAbs containing purified anti-CD16/32 (2.4G2) (Fc Block) mAb at RT (15 min). For tetramer staining, cells were first stained with APC-labeled H-2Kb/p15E(604-611)-tetramer (MBL) and, after washing, with fluorochrome-conjugated mAbs against mouse CD8 (KT15), CD45.1 and CD90.1 in the presence of Fc-Block. For analysis of chemokine receptors, cells were incubated with a mix containing the fluorophore-conjugated mAbs specific for the chemokine receptors and Fc Block at 37ºC (30 min), followed by mAbs specific for cell population markers at RT (15 min). For intracellular staining (CD36, GLUT1, granzyme B, Ki-67, IFNγ and TNFα) after incubation with Zombie NIR and fluorophore-conjugated mAb against surface markers, cells were fixed and permeabilized with Cytofix/cytoperm buffer (BD Biosciences) and then stained with the respective mAbs in the presence of purified Rat IgG. Anti-GFP mAb was added to the mix to counteract the loss of GFP intensity upon fixation. Before the intracellular detection of IFNγ and TNFα, cells were incubated with cognate peptides [OVA peptide SIINFEKL (1 μg/ml), Pmel peptide (EGSRNQDWL, 2μg/ml), p15E(604-611) peptide (KSPWFTTL, 1 μg/ml) (Neomps)], in the presence of Brefeldin A (diluted 1:1000) (Biolegend) for 4-6 hours. For intracellular staining of Foxp3, the FOXP3 Staining kit (eBiosciences) was used.

For CTV labeling, cells were resuspended in PBS at 10^6 cell/ml and mixed 1:1 (volume) with CTV (10 μM in PBS) (CTV final concentration 5μM). Cells were incubated 15 minutes in darkness at room temperature, after which 100μl of bovine serum per ml of sample were added for quenching. Lastly, cells were washed with PBS.

For staining with Annexin V, cells were surface stained with mAb as described above, washed in 1× Annexin Binding Buffer (Biolegend) and stained with PE-conjugated Annexin V and 7AAD in 1× Annexin Binding Buffer.

For glucose uptake, cells (1-5×10^5) were cultured in glucose-free RPMI medium for 60 minutes at 37ºC. Then, the glucose analog 2-NBDG was added (20 μM final concentration) and cells were cultured for an additional 30 minutes. Cells were surface stained with mAbs and labeled with 7AAD before FACS analysis.

For FA uptake, cells (1-5×10^5) were cultured in PBS supplemented with FA-free bovine serum albumin (FA free-BSA) (20μM) for 60 minutes at 37ºC in an incubator. Bodipy-FL C16, the analog of palmitic acid, was added to the cells (1μM final concentration) and they were cultured for an additional 30 minutes. Surface staining with mAbs and 7AAD labeling were performed before FACS analysis.

For lipid droplet content, cells (1-5×10^5) were surface stained with mAbs and resuspended in PBS-FA free-BSA (20 μM). Bodipy-493 was added (1 μg/ml final concentration) and cells were cultured for 60 minutes at 4ºC. 7AAD live/dead staining was performed before FACS analysis.

Chemotaxis assay

CD8 T cell migration was evaluated using 24-well, transwell plates with 5 μm pore size (Costar). Genetically modified CD8 T cells were washed and adjusted to 10^6 cells/ml in complete medium. One hundred μl of cell suspension were placed in the top chamber of the transwell. CCL22 (Inmunotool) prepared at the indicated concentration in culture medium (500 μl per well) was placed in the bottom chamber of the transwell. After 3 hours of incubation at 37ºC, the top chamber was removed and the number of cells in the bottom chamber was counted by FACS using a Cytoflex volumetric cytometer (Beckman-Coulter).

Suppression assay

$CD8^+$ splenocytes (2×10^4/well) isolated from BL6 ($CD45.2^+$) mice and labeled with CTV were activated (96-well polystyrene round-bottom microwell plates, Nunc) with anti-CD3/CD28 mAb-coated beads (2:1, bead:CD45.2 cell ratio) in the absence of IL-2. Activation occurred in co-culture with in vitro pre-activated (7 days) Foxp3UP or Mock OT-I ($CD45.1^+$) T cells, or with Foxp3UP or Mock OT-I ($CD45.1^+$) cells isolated from tumors 7 days after ACT. at different $CD45.1^+$:$CD45.2^+$ ratios. After 72 hours, cells were stained with Zombie NIR and anti-CD45.2 mAb and proliferation of $CD45.2^+$ CD8 cells was determined by CTV dilution and FACS analysis. As positive control, CD4 Treg ($CD4^+CD25^+$) cells were used. Murine $CD4^+CD25^+$ cells were isolated from the spleen of BL6 ($CD45.2^+$) mice using the $CD4^+CD25^+$ Regulatory T Cell Isolation Kit (Miltenyi) following the manufacture's recommendations.

Activation-induced cell death (AICD) Assay

Genetically modified CD8 T cells expanded i*n vitro* for 4 days were cleaned form dead cells using the Dead Cell Removal Kit (Miltenyi) following the manufacturer's instructions. Cells were cultured (10^5/well, 200 µl) in 96-well plates coated with or without anti-CD3 (1 µg/ml) in the absence of IL-2. After 24 hours of culture, samples were stained with anti-CD90.1 and anti-FASL mAbs, Annexin V and 7AAD and the surface expression of FASL and viability of transduced cells was analyzed by FACS.

IL-2 cytokine withdrawal-induced cell death (CWID) assay

Genetically modified CD8 T cells expanded *in vitro* for 8 days were cleaned from dead cells using the Dead Cell Removal Kit (Miltenyi). Cells were cultured (5×10^5/well, 1000 µl) in 48-well plates in complete medium without IL-2 for 72 hours, after which they were harvested and stained with Annexin V, 7AAD dye and anti-CD90.1 mAb. The viability of transduced cells was analyzed by FACS.

***Ova* and *Thy1.1* gene expression analysis**

RNA isolated from tumors (Maxwell 16 LEV simplyRNA tissue kit, Promega) (1 µg/sample) was treated with DNase I (Invitrogen) prior to RT with Moloney murine leukemia virus reverse transcriptase (Invitrogen) in the presence of RNaseOUT (Invitrogen) and 1/4 of cDNA preparation was used for each PCR. QPCR was performed with 200 nM of validated primers in 25 µl reactions using the Platinum SYBR Green One-Step qPCR kit (Invitrogen) and the following program: 50°C for 2 min, 95°C for 10 min, and 40 cycles of amplification at 95°C for 15 s and 62°C for 60 s. Relative levels of target mRNA were compared with *Gapdh* using the $2^{\Delta\Delta Ct}$ method, where the control cell type or treatment group was normalized to 1. Primers were obtained from Integrated DNA Technologies. *Ova*: (F) 5′-CACAAGCAATGCCTTTCAGA-3′; and (R) 5′-GAATGGATGGTCAGCCCTAA-3′; *Gapdh*: (F) 5′-AACTTTGGCATTGTGGAAGG-3′ and (R) 5′-ACACATTGGGGGTAGGAACA-3′; and *Thy1.1*: (F): GCCAACTTCACCACCAAGGATG and (R) GATGTGTTCTGAACCAGCAGGC.

Statistical analysis

The standard deviation (SD) was used to estimate the dispersion of individual observations over the mean, while SEM was used to estimate the variability of the means of different groups[3]. We used SD for the results obtained in an analytical assay, in which (for each experimental condition being compared) there were triplicate to quintuplicate analytical determinations from one representative experiment. We used SEM for the results obtained in a biological experiment, in which (for each

group being compared) there were data from different animals, or when (for each group being compared) compiled data from different experiments were available. For simple comparisons, unpaired or paired two-tailed Student *t* tests were used. Multiple comparisons were performed by two-way ANOVA followed by the Bonferroni multiple comparisons test. Tumor growth curves were compared using non-linear fit test. The FDR calculation was made using the Benjamini-Hochberg method. Differential survival was assessed using the Mantel-Cox test. Significance was set at $p < 0.05$.

REFERENCES OF SUPPLEMENTARY DATA

1. Naviaux, R.K., Costanzi, E., Haas, M. and Verma, I.M. (1996). The pCL vector system: rapid production of helper-free, high-titer, recombinant retroviruses. J. Virol. 70, 5701–5705.
2. Wu, Y., Borde. M., Heissmeyer, V., Feuerer, M., Lapan, A.D., Stroud, J.C. et al. (2006). FOXP3 Controls Regulatory T Cell Function through Cooperation with NFAT. Cell 126, 375–387.
3. Barde, P. and Barde, M. (2012). What to use to express the variability of data: Standard deviation or standard error of mean? Perspect. Clin. Res. 3, 113-116.
