# Supplementary material for: FOXP3 expression diversifies the metabolic capacity and enhances the efficacy of CD8 T cells in adoptive immunotherapy of melanoma
Source: Mol Ther. 2022 Aug 31;31(1):48–65. doi: 10.1016/j.ymthe.2022.08.017 (PMC9840123; doi:10.1016/j.ymthe.2022.08.017)
Supplement: Document S2. Article plus supplemental information [file mmc2.pdf]

Molecular Therapy
Original Article

FOXP3 expression diversifies the metabolic capacity and enhances the efficacy of CD8 T cells in adoptive immunotherapy of melanoma

Enrique Conde,[1,2] Noelia Casares,[1,2] Uxua Mancheño,[1,2] Edurne Elizalde,[1,2] Enric Vercher,[1,2] Roberto Capozzi,[1,2] Eva Santamaria,[3,4] Juan R. Rodriguez-Madoz,[2,5,6] Felipe Prosper,[2,5,6,7] Juan J. Lasarte,[1,2] Teresa Lozano,[1,2] and Sandra Hervas-Stubbs[1,2,4]

[1]Program of Immunology and Immunotherapy, Center for Applied Medical Research (CIMA), University of Navarra, Avenida Pio XII 55, Pamplona, 31008 Navarra, Spain; [2]Instituto de Investigación Sanitaria de Navarra (IdiSNA), Avenida Pio XII 55, Pamplona, 31008 Navarra, Spain; [3]Hepatology Program, CIMA, University of Navarra, Pamplona, 31008 Navarra, Spain; [4]CIBERehd, Instituto de Salud Carlos III, 28029 Madrid, Spain; [5]Hemat-Oncology Program, CIMA Universidad de Navarra, Pamplona, 31008 Navarra, Spain; [6]Centro de Investigación Biomédica en Red de Cáncer (CIBERONC), 28029 Madrid, Spain; [7]Hematology and Cell Therapy Department, Clínica Universidad de Navarra, Pamplona, 31008 Navarra, Spain

Regulatory T cells overwhelm conventional T cells in the tumor microenvironment (TME) thanks to a FOXP3-driven metabolic program that allows them to engage different metabolic pathways. Using a melanoma model of adoptive T cell therapy (ACT), we show that FOXP3 overexpression in mature CD8 T cells improved their antitumor efficacy, favoring their tumor recruitment, proliferation, and cytotoxicity. FOXP3-overexpressing (Foxp3UP) CD8 T cells exhibited features of tissue-resident memory-like and effector T cells, but not suppressor activity. Transcriptomic analysis of tumor-infiltrating Foxp3UP CD8 T cells showed positive enrichment in a wide variety of metabolic pathways, such as glycolysis, fatty acid (FA) metabolism, and oxidative phosphorylation (OXPHOS). Intratumoral Foxp3UP CD8 T cells exhibited an enhanced capacity for glucose and FA uptake as well as accumulation of intracellular lipids. Interestingly, Foxp3UP CD8 T cells compensated for the loss of mitochondrial respiration-driven ATP production by activating aerobic glycolysis. Moreover, in limiting nutrient conditions these cells engaged FA oxidation to drive OXPHOS for their energy demands. Importantly, their ability to couple glycolysis and OXPHOS allowed them to sustain proliferation under glucose restriction. Our findings demonstrate a hitherto unknown role for FOXP3 in the adaptation of CD8 T cells to TME that may enhance their efficacy in ACT.

INTRODUCTION

Adoptive T cell therapy (ACT) is a new generation of cancer treatments that could drastically change clinical strategies. However, the very biology of T cells and the tumor microenvironment (TME) limits the efficacy of ACT. Although it is necessary for the efficient control of tumor growth that transferred T cells acquire full effector functions, terminally differentiated effector T cells (TEF) have a very short half-life, and the lack of replacement of these cells limits the long-term tumor control by ACT.[1] Moreover, during their differentiation into TEF cells, T cells start expressing inhibitory receptors as a means to keep the immune response controlled.[2] Concomitantly, tumor cells evolve and express the respective ligands, thereby evading immune attack. Activation-induced cell death (AICD) upon antigen encounter also limits the ability of transferred cells to control tumors.[3,4] Along with physical barriers and the recruitment of suppressor cells, the metabolic features of the TME also represent an important hurdle for ACT.[5] Like tumor cells, activated T cells are highly dependent on glycolysis. Competition for glucose generates a state of glucose restriction in the TME and leads to the inhibition of T cell glycolysis, which in turn impairs T cell adhesion, proliferation, and effector functions. This not only affects endogenous tumor-infiltrating lymphocytes (TILs) but also transferred T cells. In this context, increased tumor-intrinsic glycolytic activity is associated with a poor response of melanoma patients to ACT.[6] Apart from glucose restriction, the TME poses other metabolic barriers for T cells, such as depletion of critical amino acids, lactate released by tumor cells and the resulting acidosis, hypoxia in poorly vascularized tumor areas,[5] and high levels of extracellular potassium that impair the absorption of nutrients.[7] All these metabolic pitfalls limit TILs in their ability to differentiate into fully competent TEF cells.[8]

Unlike conventional T cells (Tconv), forkhead box P3 (FOXP3) CD4 regulatory T cells (Tregs) can reprogram their metabolism in adverse conditions, allowing them to optimize nutrients and exploit supplemental metabolic routes.[9,10] For example, intratumoral CD4 Tregs

Received 27 January 2022; accepted 25 August 2022;
https://doi.org/10.1016/j.ymthe.2022.08.017.

Correspondence: Teresa Lozano, PhD, Center for Applied Medical Research (CIMA), University of Navarra, Avenida Pio XII 55, Pamplona, 31008 Navarra, Spain.
E-mail: tlmoreda@unav.es
Correspondence: Sandra Hervas-Stubbs, PhD, Center for Applied Medical Research (CIMA), University of Navarra, Avenida Pio XII 55, Pamplona, 31008 Navarra, Spain.
E-mail: mshervas@unav.es

 © 2022 The Author(s).
This is an open access article under the CC BY license (http://creativecommons.org/licenses/by/4.0/).

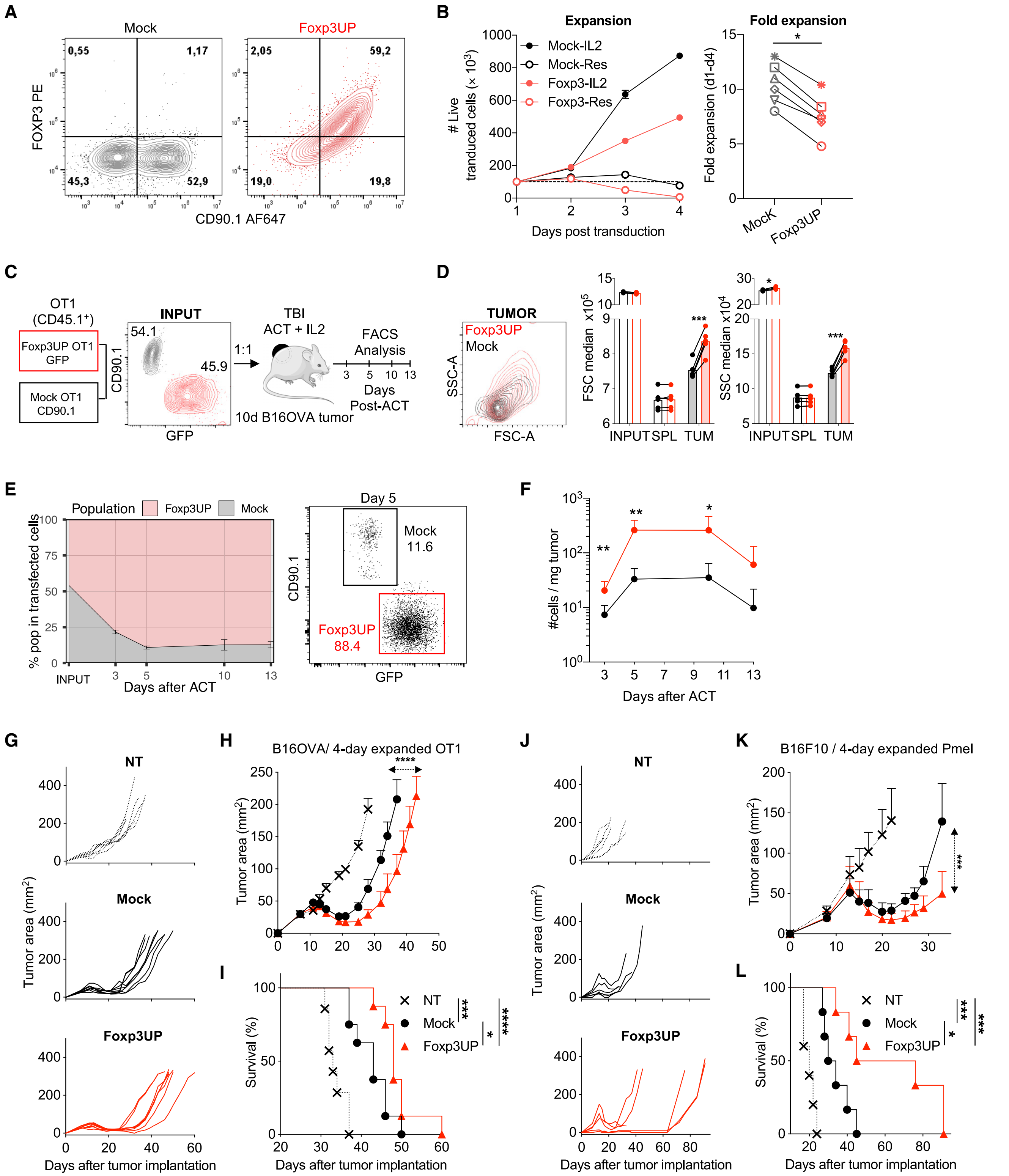

(legend on next page)

upregulate glucose transporter 1 (GLUT1)[11] and utilize glucokinase,[12] which enables them to capture glucose at high rates. Interestingly, CD4 Tregs are programmed by FOXP3 to be flexible in their fuel choice, thereby allowing them to adopt a catabolic metabolic program with increased capacity for fatty acid (FA) oxidation (FAO)-fueled oxidative phosphorylation (OXPHOS).[13] In addition, hypoxia promotes glycolysis and indirectly fosters the oxidation of FA in Tregs.[14] Furthermore, CD4 Tregs can oxidize lactate and are more resistant to lactate-mediated impairment of proliferation than Tconv.[15,16] All these metabolic advantages allow Tregs to survive and proliferate in the hostile TME overwhelming Tconv.

$FOXP3^+$ cells within the CD8 T cell subset have also been described in human and mouse tumors.[17–24] Characterization of $FOXP3^+$ CD8 T cells is difficult owing to their low frequency (0.4% and 0.1% of circulating T cells in humans and mice, respectively) and because their isolation is hampered by the localization of FOXP3 in the nucleus. Currently, it is not known how these cells develop *in vivo*, nor the metabolic program that supports them. Within $CD8^+FOXP3^+$ T cells in cancer, there is evidence for an immunosuppressive population,[17–20] but also for effector cells with no suppressor hallmarks.[21,22] Recently, in cervical cancer and melanoma, elevated levels of $CD8^+FOXP3^+$ T cells were observed in responders to anti-PD-1 therapy.[23,24] This subset was characterized by an early effector memory phenotype with intermediate PD-1 levels and co-expression of several inhibitory receptors, but, rather than being exhausted, they produced higher levels of granzyme-B (GzmB) and effector cytokines as compared with their $CD8^+FOXP3^-$ counterparts.[23] Similarly, activated Tconv transiently express some FOXP3 yet show no suppressive properties.[25,26] The fact that other factors are necessary to support FOXP3 in generating regulatory properties[27,28] could explain why FOXP3 expression is not always associated with suppressor functions.[21–24,29]

Given the ability of CD4 Tregs to adapt their metabolism to survive and proliferate in the hostile TME,[11] with FOXP3 driving this adaptation,[13] and the suggestive findings about the putative role of $FOXP3^+$ CD8 T cells as tumor-specific effector T cells,[21,22] we decided to overexpress FOXP3 in mature CD8 T cells and study how this affected their metabolic competences and antitumor efficacy in adoptive immunotherapy for melanoma.

RESULTS

FOXP3 overexpression in CD8 T cells improved their tumor recruitment and therapeutic efficacy in ACT

To determine whether FOXP3 overexpression in mature CD8 T cells affected their antitumor properties, murine CD8 T cells were infected with an empty or FOXP3-encoding retrovirus (RV) to generate mock (modified with empty vectors) and Foxp3UP CD8 T cells, respectively (Figure 1A). Expression of FOXP3 in CD8 T cells impaired their expansion *in vitro* (Figure 1B), mainly due to a lower survival capacity under standard T cell culture conditions (Figures S1A–S1D), resembling behavior of CD4 Tregs in similar settings.[30]

To evaluate the behavior of Foxp3UP CD8 T cells in ACT schedules, mock ($CD90.1^+$) and Foxp3UP (GFP^+) OT-I cells were mixed (1:1) and transferred into B16OVA tumor-bearing mice (Figure 1C). Before transfer, Foxp3UP OT-I cells exhibited similar size (forward scatter [FSC] values) but slightly higher complexity (side scatter [SSC] values) than mock cells. However, after transfer, Foxp3UP CD8 T cells showed larger size and complexity than their mock counterparts in the tumor, whereas in the spleen they both exhibited similar FSC and SSC values (Figure 1D). This suggested that Foxp3UP CD8 T cells were likely more activated than mock cells in the tumor. Notably, overexpression of FOXP3 markedly increased the number of Foxp3UP CD8 TILs from day 5 onward (Figures 1E and 1F). To further evaluate the effect of FOXP3 overexpression on the therapeutic efficacy of CD8 T cells, 4-day *in vitro*-expanded Foxp3UP or mock OT-I cells were adoptively transferred to B16OVA tumor-bearing mice. Notably, Foxp3UP CD8 T cells significantly restrained tumor growth and enhanced overall survival (Figures 1G–1I). The enhanced antitumor efficacy of FOXP3-overexpressing CD8 T cells was also verified using Pmel cells in B16F10 tumor-bearing mice (Figures 1J–1L). As previously described,[31] prolonged culture cells (7-day *in vitro*-expanded cells) exhibited modest long-term tumor control, but even then Foxp3UP Pmel cells were more efficient at controlling early tumor growth (Figures S1E–S1G).

Figure 1. FOXP3 overexpression in CD8 T cells improved their antitumor efficacy in ACT

$CD8^+$ splenocytes were activated, and 48 h later they were infected with an empty RV or an RV encoding FOXP3 to generate mock and Foxp3UP CD8 T cells, respectively. (A) Detection of FOXP3 in transduced ($CD90.1^+$) CD8 T cells 2 days after retroviral infection (assessed by intracellular staining and FACS). (B) After infection, cells were cultured with or without (Res) IL-2 for 4 days. Left: the absolute number (#) of live transduced cells ($7AAD^-CD90.1^+$) was determined using a volumetric cytometer (Cytoflex). Right: fold expansion (between days 1 and 4 after transduction) of CD8 T cells cultured with IL-2. Compiled data from six different experiments. (C) Four-day *in vitro*-expanded Foxp3UP (GFP^+) and mock ($CD90.1^+$) OT-I ($CD45.1^+$) cells were mixed 1:1 and injected (i.v.) into TBI BL6 ($CD45.2^+$) mice bearing 10-day-established B16OVA tumors (n = 6). Foxp3UP ($GFP^+CD45.1^+$) and mock ($CD90.1^+CD45.1^+$) cells inside tumors were analyzed by FACS at different times after transfer. The contour plot shows the mixed cell input. (D) Overlay dot plot showing forward scatter (FSC) and side scatter (SSC) parameters of transduced cells within the tumor at day 5 of ACT. Bar graphs show FSC and SSC median values of Foxp3UP and mock CD8 T cells before transfer (INPUT) and at day 5 of ACT in the spleen (SPL) and tumor (TUM). (E) Graph showing the percentage of Foxp3UP and mock cells in total transduced (GPF^+ plus $CD90.1^+$) $CD45.1^+$ TILs on days 0 (INPUT), 3, 5, 10, and 13. A representative dot plot of day 5 is also shown. (F) Number of Foxp3UP and mock cells normalized to mg of tumor. (H–L) Effect of FOXP3 overexpression on the antitumor properties of CD8 T cells. (G–I) Eight-day B16OVA tumor-bearing mice were treated with 4-day *in vitro*-expanded Foxp3UP or mock OT-I cells (2×10^6) (8 mice/group). (J–L) Ten-day B16F10 tumor-bearing mice were treated with 4-day *in vitro*-expanded Foxp3UP or mock Pmel cells (4×10^6) (6 mice/group). Tumor size (mm^2) from individual mice (G and J), average tumor size (H and K), and overall survival (I and L). Data are presented as mean (D), mean ± SD (B, left), and mean ± SEM (E, F, H, and K). Symbols represent individual mice (D) or experiments (B, right). Statistical significance was determined using paired t test (B [right], D, and F), non-linear regression (curve fit) (H and K), and Mantel-Cox test (I and L). ***$p < 0.0005$, **$p < 0.005$, *$p < 0.05$. One experiment was representative of two (B [left] and E–L) or eight (A and D) experiments.

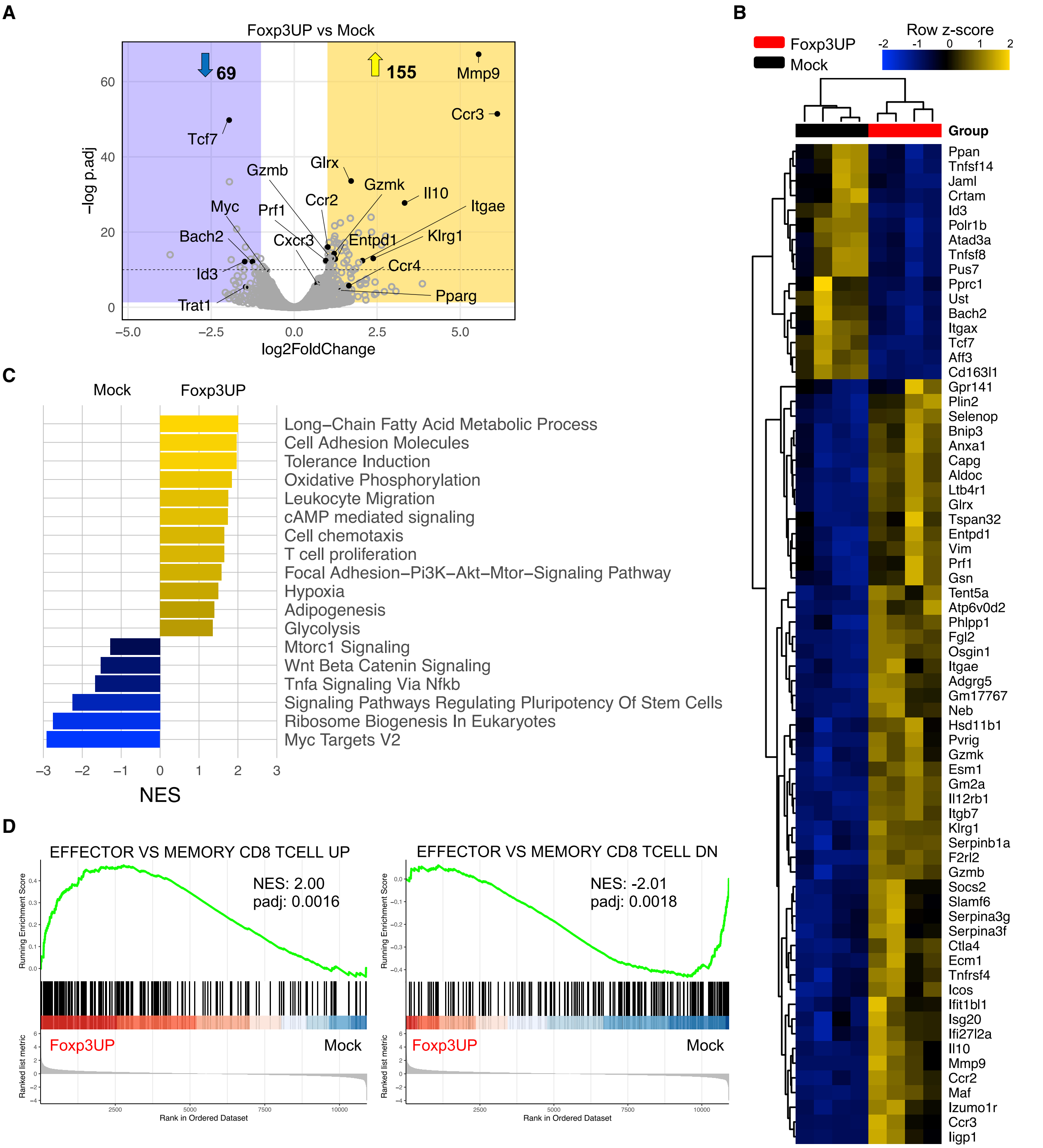

Figure 2. Transcriptomic signature of Foxp3UP TILs

(A–D) TBI 10-day B16OVA-bearing BL6 ($CD45.2^+$) mice (n = 5) received an i.v. injection of a mix containing 4-day *in vitro*-expanded mock ($CD90.1^+$) and Foxp3UP (GFP^+) (1:1 ratio) OT-I ($CD45.1^+$) cells. Five days later, Foxp3UP ($GFP^+CD45.1^+$) and mock ($CD90.1^+CD45.1^+$) CD8 T cells infiltrating the tumor were separately isolated by FACS and used for RNA-seq. Differentially expressed gene (DEG) analysis using Foxp3UP and mock CD8 T cells from four independent experiments was performed. (A) Volcano plot

(legend continued on next page)

FOXP3-overexpressing CD8 T cells exhibited a diverse metabolic transcriptional program and a TEF cell gene signature within the tumor

To explore the effect of FOXP3 in the transcriptional program of transferred CD8 T cells, we isolated tumor-infiltrating Foxp3UP and mock OT-I cells at day 5 of ACT and analyzed them by RNA sequencing (RNA-seq). The two most upregulated genes in Foxp3UP CD8 TILs were *Ccr3* and *Mmp9* (Figures 2A and 2B). Genes involved in cytotoxicity, such as *GzmB*, *GzmK*, and *Prf1*, were also upregulated in these cells. Notably, *Itgae* and *Entpd1*, encoding for CD103 and CD39, respectively, were also upregulated in Foxp3UP CD8 TILs. Co-expression of CD103 and CD39 identifies tumor-reactive CD8 TILs with tissue-resident memory T (TRM) cell features.[32] Enrichment analysis comparing CD39$^+$CD103$^+$CD8$^+$ TIL signature (GSE114944)[32] with our dataset portrayed significant similarities with Foxp3UP CD8 TILs (Figure S2A). Importantly, gene set enrichment analysis (GSEA) using clusterProfiler showed positive enrichment of metabolic pathways, such as glycolysis, FA metabolism, adipogenesis, and OXPHOS in Foxp3UP CD8 TILs (Figure 2C). The genetic signatures related to T cell proliferation, chemotaxis, leukocyte migration, cell adhesion, hypoxia, PI3K-AKT and cAMP signaling, and tolerance induction were also positively enriched in Foxp3UP CD8 TILs. In contrast, genes involved in mTORC1 signaling, Myc targets, tumor necrosis factor α (TNFα) signaling, and ribosome biogenesis were negatively enriched. Consequent with downregulation of *Tcf7*, *Id3*, and *Bach2* (Figures 2A and 2B), the WNT β-catenin signaling pathway and other pathways regulating the pluripotency of stem cells appeared negatively enriched in Foxp3UP CD8 TILs (Figure 2C). Notably, the GSEA of the Foxp3UP/mock cell comparison prior to infusion and after ACT in the spleen did not reveal significant enrichment in any of these pathways (Table S1).

Given the positive enrichment in cell migration, adhesion and proliferation gene signatures, and the negative enrichment in WNT β-catenin signaling in Foxp3UP CD8 TILs, we hypothesized that FOXP3 could promote TEF cell differentiation within the TME. Comparing the GSEA of our Foxp3UP/mock dataset with published immunologic signature gene set collections from the Molecular Signatures Database (MSigDB) revealed that gene transcripts associated with TEF cells were significantly enriched in Foxp3UP cells infiltrating the tumors (Figure 2D) but not in cells prior to transfer (Figure S2B), nor in transferred cells isolated from the spleen (Figure S2C).

The expression of FOXP3 in CD8 T cells did not endow them with suppressive activity

RNA-seq data indicated that genes related to tolerance induction, mainly CD4 Treg hallmark genes (such as *Il10* and *Ctla4*), were enriched in Foxp3UP CD8 TILs (Figures 2A–2C). To find out whether overexpression of FOXP3 in CD8 T cells conferred suppressive properties, we performed a classic suppression assay by activating CD8 T cells (CD45.2$^+$) in the presence of Foxp3UP or mock CD8 T cells (CD45.1$^+$), either expanded *in vitro* (Figures 3A and 3B) or isolated from tumors after ACT (Figures 3C and 3D). As shown in Figure 3, Foxp3UP CD8 T cells did not differ from mock cells in their suppressive activity on recently activated CD8 T cells.

To further confirm that Foxp3UP CD8 T cells were devoid of suppressive properties, we injected mock GFP$^+$ OT-I cells together with mock CD90.1$^+$ OT-I cells or Foxp3UP CD90.1$^+$ OT-I cells (mix 1:1) into B16OVA tumor-bearing mice and analyzed the number of mock GFP$^+$ OT-I cells in the tumor at day 7 of ACT. As depicted in Figure 3E, the number of mock GFP$^+$ OT-I cells was similar regardless of whether they had been injected with mock CD90.1$^+$ OT-I cells or with Foxp3UP CD90.1$^+$ OT-I cells. In addition, we assessed the effect of Foxp3UP CD8 T cells on endogenous tumor-specific T cells. To this end, we analyzed the frequency of T cells specific for the p15E antigen, a tumor-associated antigen of B16F10-derived cell lines.[33] We chose this T cell population because it did not compete with the transferred OT-I cells for the antigen. The percentage of endogenous TILs specific for the H-2Kb-restricted p15E(604–611) epitope was similar (as shown by tetramer staining) regardless of whether or not mice had received Foxp3UP OT-I cells (Figure 3F). The same was observed when tumor cell suspensions were stimulated with p15E(604–611) peptide and the production of interferon-γ (IFNγ) and TNFα was analyzed in endogenous CD8 TILs (Figure 3G). Taken together, these data clearly indicate that FOXP3 expression on CD8 T cells does not confer suppressive activity.

The increased number of Foxp3UP CD8 T cells in the tumor infiltrate may be due to their enhanced ability to migrate and proliferate within the tumor

Since transcriptomic data showed upregulation of genes related to T cell proliferation (Figure 2C), we sought to investigate the proliferative capacity of transferred Foxp3UP CD8 T cells. Thus, Foxp3UP (GFP$^+$) and mock (CD90.1$^+$) OT-I cells were mixed (1:1), labeled with cell trace violet (CTV), and adoptively transferred into tumor-bearing mice. On day 3 of ACT, they were analyzed in tumors and the spleen (Figure 4A). In agreement with our previous data (Figures 1E and 1F), overexpression of FOXP3 markedly increased the presence of transferred CD8 T cells within the tumor (Figures 4B–4D, S3A, and S3B). However, this difference was not seen in the spleen. As depicted by the CTV dilution assay, at day 3 of ACT transferred cells infiltrating the spleen have proliferated more than those in the tumor (lower CTV median fluorescence intensity [MFI] values, or higher reverse MFI [1/CTV] values, in spleen than in tumor) (Figures 4E and S3C). The recent *in vitro* activation and/or homeostatic signals after ACT may have driven the proliferation of transferred cells in the spleen, while the antigen encounter and TME signals may also have influenced their proliferation in the

depicting DEGs of interest (cutoff $\log_2$ fold change > 1, p_{adj} < 0.05). (B) Heatmap representation of hierarchical clustering of highly significant DEGs ($p_{adj} < 10^{-10}$). (C) GSEA by clusterProfiler illustrates gene sets positively (normalized enrichment score [NES] > 0) or negatively (NES < 0) enriched in Foxp3UP versus mock TILs. All depicted pathways reached the false discovery rate <0.05 cutoff. (D) GSEA enrichment score curve of "Effector versus memory CD8 T cell" upregulated (UP) and downregulated (DN) gene sets in Fox3UP versus mock TILs presented as the NES.

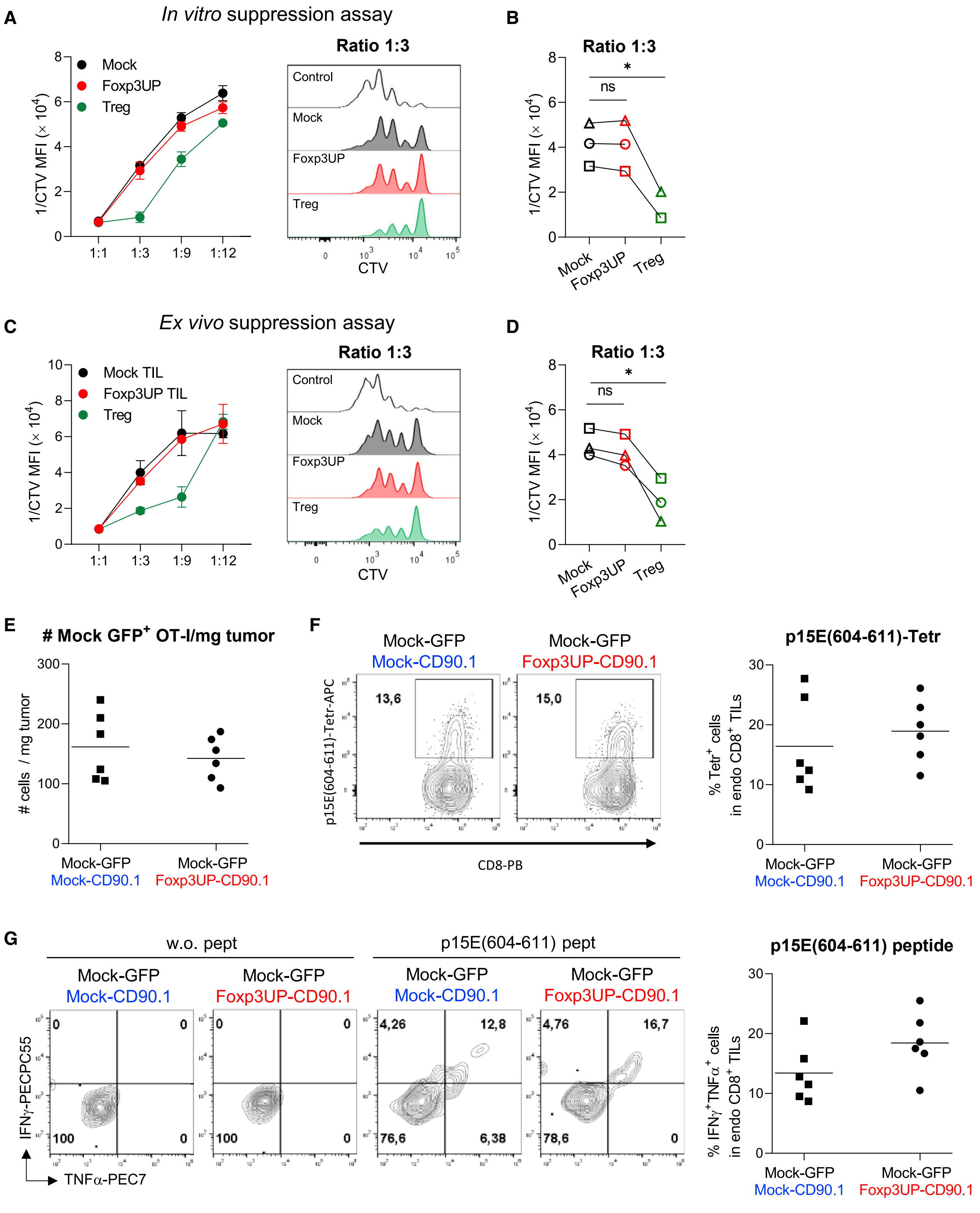

(legend on next page)

tumor. Interestingly, Foxp3UP OT-I cells exhibited a significantly more extensive proliferation than mock cells in the tumor but not in the spleen, where mock OT-I proliferated slightly better (Figures 4E and S3C). Ki-67 staining of OT-I cells on day 7 confirmed the data observed with the CTV dilution assay (Figure 4F). Interestingly, on this day OT-I cells from the spleen showed a lower level of Ki-67 expression than their counterparts in the tumor, indicating that the extensive proliferation seen outside the tumor on day 3 had slowed.

Viability between Foxp3UP and mock CD8 T cells did not differ in tumors and the spleen (Figure S4A). Because the ingestion of apoptotic cells by macrophages could potentially impair the detection of dead cells *in vivo*, we decided to study apoptosis *in vitro*. Interestingly, forced FOXP3 expression in CD8 T cells protected them from AICD (Figure S4B). Upregulation of FAS ligand (FASL) is intimately associated with AICD in TEF cells.[34] Notably, *in vitro* restimulation induced higher levels of FASL expression on the surface of mock CD8 T cells as compared with Foxp3UP CD8 T cells (Figure S4C). Cytokine-withdrawal-induced cell death also compromises survival of TEF cells.[35] Viability of activated CD8 T cells *in vitro* was seriously compromised in the absence of interleukin-2 (IL-2), with Foxp3UP CD8 T cells being more affected by IL-2 withdrawal (Figure S4D). Interestingly, Foxp3UP CD8 T cells had higher expression levels of CD25 (IL-2Rα) than their mock counterparts (Figure S4E).

Consistent with the enrichment of gene sets related to cell chemotaxis and leukocyte migration (Figure 2C), high expression of genes coding for chemokine receptors was found in Foxp3UP CD8 TILs (Figure S5A). To confirm RNA-seq data, we analyzed the expression of various chemokine receptors before and after ACT by flow cytometry (fluorescence-activated cell sorting [FACS]). Prior to transfer, no differences in the expression of CCR3, CCR7, CXCR3, and CX3CR1 were observed between Foxp3UP and mock CD8 T cells (Figure S5B). However, Foxp3UP CD8 T cells exhibited higher expression levels of CCR4 (Figure 4G). CCR4 is normally expressed in CD4 Tregs and mediates their immigration to tumors in response to the chemokine C-C motif ligand 22 (CCL22).[36] Similarly, Foxp3UP CD8 T cells migrated more efficiently in response to CCL22 than mock CD8 T cells (Figure 4H). Notably, after ACT, Foxp3Up CD8 T cells infiltrating tumors and draining lymph nodes (dLNs) expressed even higher levels of CCR4 than their mock counterparts (Figure 4I). The same was observed for CCR3. Compared with mock cells, Foxp3UP CD8 T cells expressed higher levels of CXCR3 in the dLNs, whereas they expressed less CX3CR1 in tumors. No differences were observed between Foxp3UP and mock cells regarding CCR7 expression. Given the role of CCR4 and CCR3 in recruiting T cells and other immune cells to the tumor,[37] the enhanced expression of these chemokine receptors on transferred Foxp3UP CD8 T cells may also explain their increased numbers in the tumors.

Foxp3UP-overexpressing CD8 T cells exhibited intense cytotoxic activity

Foxp3UP CD8 TILs displayed increased expression of key cytotoxic genes (Figure 5A). FACS analysis confirmed that transferred Foxp3UP CD8 T cells expressed higher levels of GzmB than mock CD8 T cells in the tumor (Figure 5B). However, before transfer (Figure S6A) and after transfer in the spleen and dLNs (Figure 5B), their GzmB content was similar to that of mock cells. GzmB expression in Foxp3UP CD8 TILs was also higher than in endogenous TILs (Figure 5B). It was previously reported that hypoxia promotes the expression of GzmB in CD8 T cells.[38] Interestingly, Foxp3UP CD8 T cells produced higher levels of GzmB than their mock counterparts when cultured *in vitro* under hypoxic conditions (Figure S6B). Furthermore, incubation with tumor cells expressing the cognate antigen increased GzmB expression in CD8 T cells, especially in Foxp3UP CD8 T cells (Figure S6C). Finally, Foxp3UP CD8 T cells exhibited greater killing activity against tumor cells naturally expressing their cognate antigen (Figures 5C and 5D) and unrelated syngeneic tumor cells pulsed with the cognate epitope (Figure 5E). Only at a saturated concentration of the cognate antigen (peptide-pulsed B16OVA cells) did mock CD8 T cells reach the killing activity of Foxp3UP cells (Figure 5E). Strikingly,

Figure 3. The expression of FOXP3 in CD8 T cells did not lead to suppressive activity

(A and B) *In vitro* T cell proliferation suppression assay. $CD8^+$ splenocytes were isolated from BL6 ($CD45.2^+$) mice and labeled with CTV. Cells were activated with anti-CD3/CD28 mAb-coated beads and cultured either alone or in the presence of 7-day *in vitro*-expanded Foxp3UP or mock ($CD45.1^+$) OT-I cells at different "suppressor" ($CD45.1^+$):effector ($CD45.2^+$) cell ratios (5 replicates/condition). After 72 h, proliferation of $CD45.2^+CD8^+$ cells was determined by FACS analysis. (C and D) *Ex vivo* T cell proliferation suppression assay. Eight-day B16OVA tumor-bearing BL6 ($CD45.2^+$) mice received an i.v. injection of a mix containing 4-day *in vitro*-expanded mock ($CD90.1^+$) and Foxp3UP (GFP^+) OT-I ($CD45.1^+$) (1:1 ratio) cells. Seven days later, Foxp3UP ($GFP^+CD45.1^+$) and mock ($CD90.1^+CD45.1^+$) CD8 T cells infiltrating the tumor were separately isolated by FACS and tested *ex vivo* in a suppression assay as described in (A) and (B) (3 replicates/condition). (A–D) Data in the graphs are plotted as the reverse of CTV MFI (1/CTV MFI). High 1/CTV MFI values signify higher proliferation of CD8 $CD45.2^+$ cells and therefore less suppressive activity of $CD45.1^+$ cells. (A and C, left) Graphs showing 1/CTV MFI values at different cell ratios. (A and C, right) Histograms of CTV MFI values at ratio 1:3. In (B) and (D), data were compiled from three independent experiments. In (A–D), CD4 Tregs ($CD45.1^+CD4^+CD25^+$) were used as positive control (3 replicates/condition). (E–G) A mixture (1:1) of mock GFP^+ OT-I cells together with mock $CD90.1^+$ OT-I cells or Foxp3UP $CD90.1^+$ OT-I cells (total 4×10^6 cells) was injected into B16OVA tumor-bearing BL6 mice and mock GFP^+ OT-I, and endogenous CD8 TILs were analyzed at day 7 of transfer. (E) Number of mock GFP^+ OT-I TILs per mg of tumor. (F) Percentage of p15E(604–611)-specific endogenous CD8 TILs, as determined by tetramer staining. Representative dot plots are shown on the left. Cells were gated on $CD45.2^+CD8^+$ (endo CD8) T cells. (G) At day 7 of ACT, total cells from tumors were restimulated *ex vivo* with or without p15E(604–611) peptide, and production of IFNγ and TNFα was assessed 5 h later by FACS. Left: representative dot plots. Cells were gated on $CD45.2^+CD8^+$ (endo CD8) T cells. Right: percentage of $IFN\gamma^+TNF\alpha^+$ cells within endogenous CD8 TILs after peptide stimulation. Data are presented as mean (E, F, and G graphs) and mean ± SD (A and C). Symbols represent individual mice (E, F, and G graphs) or independent experiments (B and D). Statistical significance was determined using two-way ANOVA for multiple comparisons (B and D) or unpaired t test (E, F, and G graphs). *$p < 0.05$. One experiment was representative of two (E–G) or three (A and C) experiments.

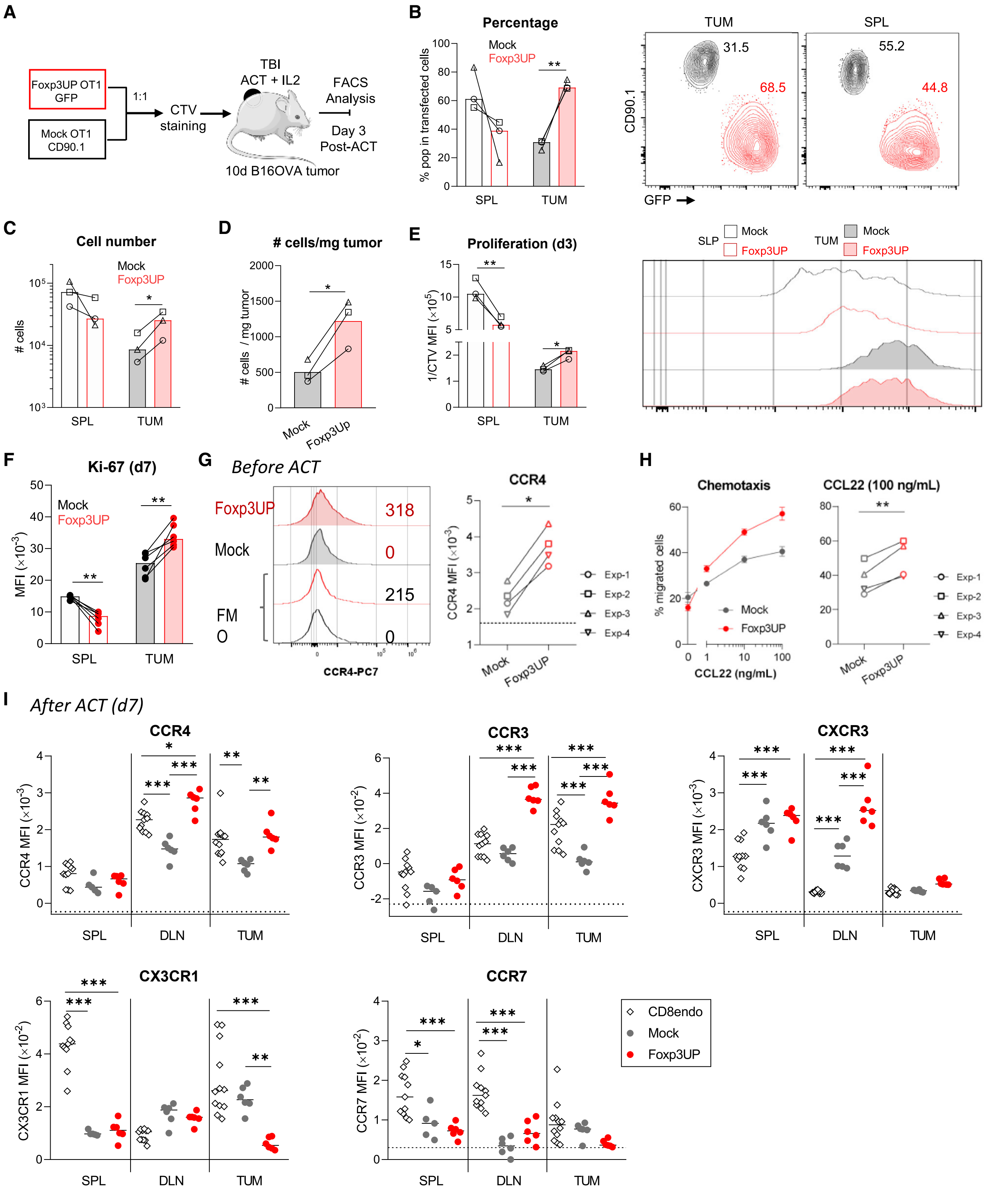

(legend on next page)

despite their TEF cell gene signature and high cytotoxic activity, Foxp3UP CD8 T cells isolated from tumors and dLNs rendered significantly less IFNγ$^+$TNFα$^+$-producing cells when restimulated *ex vivo* with the cognate antigen (Figure 5F). When stimulated with peptide, anti-CD3 monoclonal antibody (mAb), or phorbol 12-myristate 13-acetate (PMA) *in vitro*, Foxp3UP CD8 T cells produced similar or even lower levels of IFNγ and TNFα than their mock counterparts (under both normoxic and hypoxic conditions) (Figures S6D and S6E). Together, these observations indicated that FOXP3 overexpression in CD8 T cells amplified their cytotoxic activity while impairing production of IFNγ and TNFα. However, the enhanced intratumoral accumulation of Foxp3UP CD8 T cells (Figures 1E and 1F) counteracted the negative effect of FoxP3 overexpression on cytokine production and resulted in a higher number of IFNγ$^+$ TNFα$^+$ CD8 T cells within the tumor (Figure 5G).

Foxp3UP CD8 T cells outnumbered mock cells and exhibited a greater ability to control tumor growth in the late phase of ACT, at the expense of becoming highly differentiated and exhausted

RNA-seq data indicated that Foxp3UP CD8 TILs expressed some characteristic markers of TRM cells. On the other hand, these cells also exhibited a TEF cell gene signature. To assess the differentiation and exhaustion state of these cells, we analyzed the expression of several surface markers related to T cell memory,[39] activation, and exhaustion by FACS. Prior to infusion, Foxp3UP CD8 T cells exhibited slightly higher expression levels of CD62L and CD69, and, most markedly, CD103 (Figures S7A and S7B). At day 7 of ACT, the bulk of Foxp3UP CD8 T cells infiltrating dLNs and tumors showed higher expression levels of CD62L, CD27, and CD103 compared with mock cells. However, Foxp3UP CD8 T cell in the tumor exhibited a more activated and differentiated phenotype with higher expression of CD43 (130-kD), CD69, CD39, and KLRG1 (Figure S7C). Strikingly, while Foxp3UP CD8 TILs expressed more TIM-3 than mock cells, the opposite was true for LAG-3. No differences in PD-1 levels were observed.

CD103 is one of the most characteristic markers of TRM cells.[40] Other markers, such as CD69, CD39, and CD49a, may also be co-expressed with CD103, defining this population.[40] Interestingly, compared with mock cells, Foxp3UP CD8 T cells had a higher proportion of CD103$^+$ cells in spleens, dLNs, and tumors and a higher proportion of CD103$^+$CD69$^+$CD39$^+$ cells in tumors (Figure S7D). KLRG1 has been postulated to be a marker of highly differentiated T cells.[39] The percentage of KLRG1$^+$ cells was also higher within the Foxp3UP CD8 TIL population (Figure S7D). Notably, KLRG1 and CD103 identified almost mutually exclusive populations in OT1 TILs, with CD103$^+$ cells dominating over KLRG1$^+$ cells at day 7 of ACT (Figures S7E and S7F). KLRG1$^+$ Foxp3UP CD8 TILs expressed lower levels of CD103 than their KLRG1$^-$ counterparts (Figure S7F). Moreover, KLRG1$^+$ cells in both Foxp3UP and mock TIL subsets expressed lower levels of CD62L and CD27, and higher levels of PD-1, TIM-3, and LAG-3 compared with KLRG1$^-$ cells, indicating that they were more differentiated and exhausted (Figure S7G). Within Foxp3UP CD8 TILs, KLRG1$^+$ cells also expressed higher levels of the activation markers CD69 and CD43 (130-kD). By contrast, hardly any differences were observed in the expression of these markers when CD103$^+$ and CD103$^-$ cells were compared (Figures S7F and S7H). These results indicated that two populations co-existed within FOXP3 CD8 TILs: a population expressing CD103, CD62L, and CD27 (also present in dLNs although much less activated), and a more differentiated and exhausted subset expressing KLRG1.

We also addressed the differentiation state of FoxP3UP CD8 T cells at day 25 of ACT. We chose this day because at this time point mock group tumors began to grow, while in the Foxp3UP group tumors were still contained (Figures 1H and S8A). At day 25, Foxp3UP CD8 T cells outnumbered mock cells in the tumor, while both populations were barely detectable in spleen and dLNs (Figure S8B). Interestingly, Foxp3UP CD8 TILs still expressed higher levels of CCR3 than their mock counterparts (Figure S8C), but no difference was observed in terms of CCR4 expression (data not shown). At this time point, the bulk of Foxp3UP CD8 TILs exhibited a more differentiated and exhausted phenotype (Figure S8C), and the frequency of CD103$^+$ and KLRG1$^+$ cells was more balanced (Figure S8D). Strikingly, these highly differentiated and exhausted populations

Figure 4. Effect of FOXP3 overexpression on the proliferation and chemotactic properties of CD8 T cells

(A–E) Four-day *in vitro*-expanded Foxp3UP (GFP$^+$) and mock (CD90.1$^+$) OT1 (CD45.1$^+$) cells were mixed (1:1 ratio), labeled with CTV dye, and injected (i.v.) into 10-day B16OVA-bearing BL6 (CD45.2$^+$) mice (n = 3). Foxp3UP (GFP$^+$CD45.1$^+$) and mock (CD90.1$^+$CD45.1$^+$) CD8 T cells were analyzed in tumor and the spleen at day 3. (A) Schematic diagram of the experimental approach. (B) Graphs and representative dot plots showing the percentage of Foxp3UP and mock CD8 T cells in total transduced (GPF$^+$ plus CD90.1$^+$) CD45.1$^+$ cells in tumor (TUM) and the spleen (SPL). (C) Total number of Foxp3UP and mock CD8 T cells in tumor and the spleen. (D) Number of Foxp3UP and mock CD8 T cells normalized to mg of tumor. (E) Graphs showing proliferation (depicted as reverse of CTV MFI [1/CTV MFI]) of Foxp3UP and mock CD8 T cells in tumor and the spleen, with representative histogram on the right. (F) Foxp3UP and mock OT1 cells were injected (i.v.) into 10-day B16OVA-bearing BL6 mice. The expression of Ki-67 was assessed (FACS) in Foxp3UP and mock CD8 T cells from spleen and tumors at day 7. (G) Expression of CCR4 in 4-day *in vitro*-expanded Foxp3UP and mock OT-I cells. Left: representative histogram. As negative control, fluorescence minus one (FMO) is shown. Right: compiled data from four independent experiments. Dotted line indicates FMO values. (H) Transwell migration assay. Left: percentage of migrated cells in response to increasing concentrations of mouse CCL22 chemokine after 3 h of incubation. Right: Compiled data from four independent experiments. (I) Four-day *in vitro*-expanded Foxp3UP and mock OT1 cells were injected (i.v.) separately into 10-day B16OVA-bearing BL6 mice. The expression of different chemokine receptors was assessed (FACS) in Foxp3UP and mock CD8 T cells from spleen, dLNs, and tumors at day 7 of ACT. Graphs show MFIs of each receptor. Dotted lines indicate FMO values. As reference, endogenous (endo) CD8 T cells are shown. Data are presented as mean (bars in B–F and I) and mean ± SD (H, left). Symbols represent individual mice (B–F and I) or experiments (G and H [right]). Statistical significance was determined using paired t test (B–F, G, and H [right]) and two-way ANOVA for multiple comparisons (I). ***$p < 0.0005$, **$p < 0.005$, *$p < 0.05$. One experiment was representative of two experiments (B–F and I).

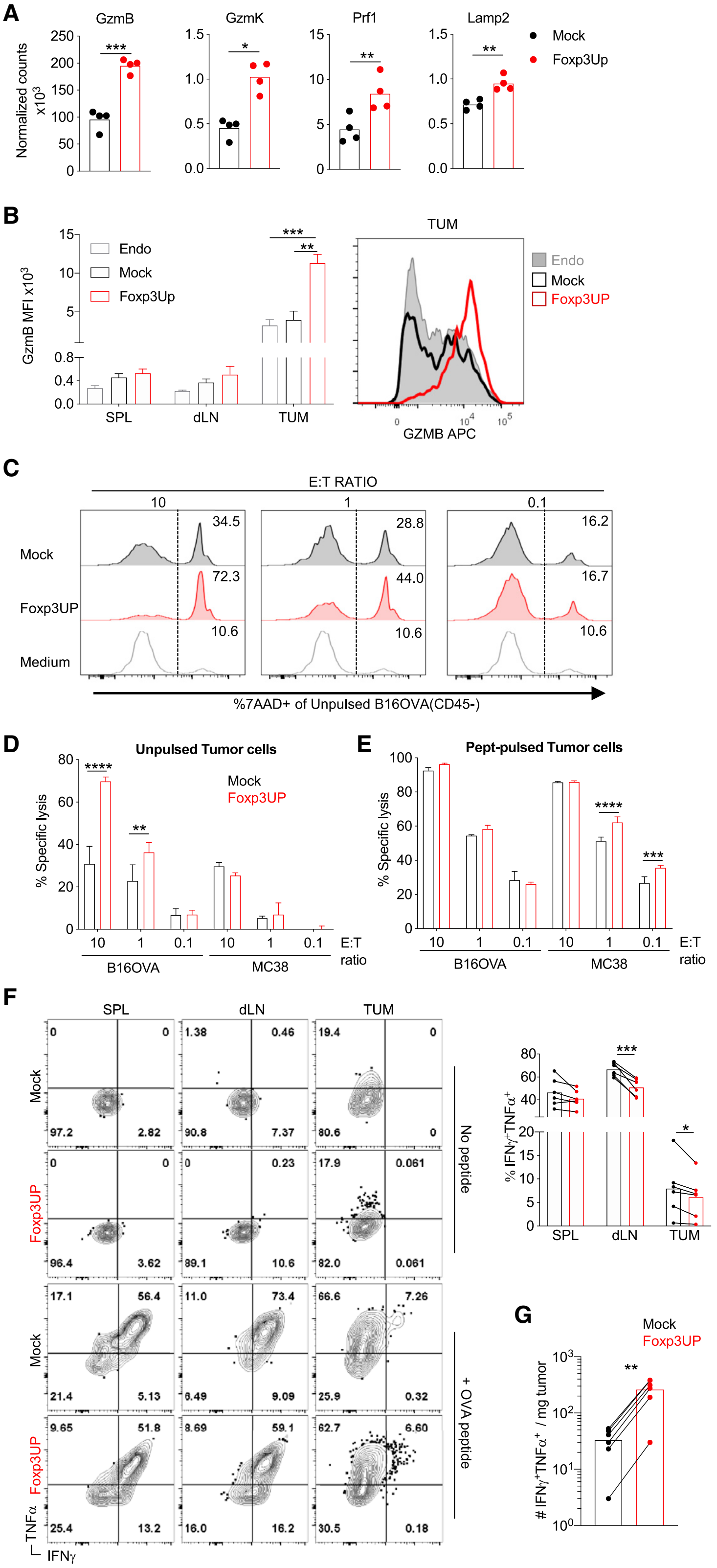

Figure 5. Enforced FOXP3 expression increased cytotoxicity in CD8 T cells

(A and B) Foxp3UP (GFP^+) and mock ($CD90.1^+$) OT-I ($CD45.1^+$) cells were co-injected at a 1:1 ratio as in Figure 2. (A) Normalized counts from an RNA-seq analysis of key cytotoxic genes in Foxp3UP ($GFP^+CD45.1^+$) and mock ($CD90.1^+CD45.1^+$) CD8 T cells isolated from tumors. (B) The graph on the left shows GzmB median fluorescent intensity (MFI) in endogenous ($CD45.2^+$) CD8 T cells (Endo) and transferred Foxp3UP ($GFP^+CD45.1^+$) and mock ($CD90.1^+CD45.1^+$) CD8 T cells in the spleen (SPL), dLNs, and tumor (TUM). Histogram on the right depicts GzmB expression in endogenous and transferred TILs. (C–E) mock or Foxp3UP Pmel cells were co-cultured with B16OVA or MC38 tumor cells that had been previously pulsed or not (unpulsed) with Pmel peptide, at different ratios of effector cells to tumor cells (E:T ratio). As a control, tumor cells were cultured alone (medium). The percentage of dead tumor cells ($7AAD^+CD45^-$) in total tumor cells ($CD45^-$) was analyzed 12 h later by FACS. (C) Representative histograms showing percentage of dead B16OVA cells at different E:T ratios. (D and E) Killing activity of Pmel cells against unpulsed (D) or pulsed (E) tumor cells depicted as the percentage of specific lysis, as described in materials and methods. (F and G) Mice were treated as in (B). At day 5 of ACT, total cells from the spleen, dLNs, and tumors were restimulated *ex vivo* with or without OVA peptide, and production of IFNγ and TNFα was assessed 5 h later by FACS. (F) Representative dot plots of cells stimulated with or without peptide (left). The graph on the right shows the percentage of $IFN\gamma^+TNF\alpha^+$ cells within Foxp3UP ($GFP^+CD45.1^+$) and mock ($CD90.1^+CD45.1^+$) CD8 T cells in spleen, dLNs, and tumor upon peptide restimulation. (G) Number of $IFN\gamma^+TNF\alpha^+$ Foxp3UP and mock OT-I TILs normalized to mg of tumor. Data are presented as mean (A, F, and G), mean + SEM (B), and mean ± SD (D and E). Symbols represent individual mice (F and G) or experiments (A). Statistical significance was determined using unpaired t test (A), paired t test (B, F, and G) and two-way ANOVA for multiple comparisons (D and E). ****$p < 0.00005$, ***$p < 0.0005$, **$p < 0.005$, *$p < 0.05$. Compiled data from four different experiments (A) or one experiment representative of two experiments (B–G) are shown.

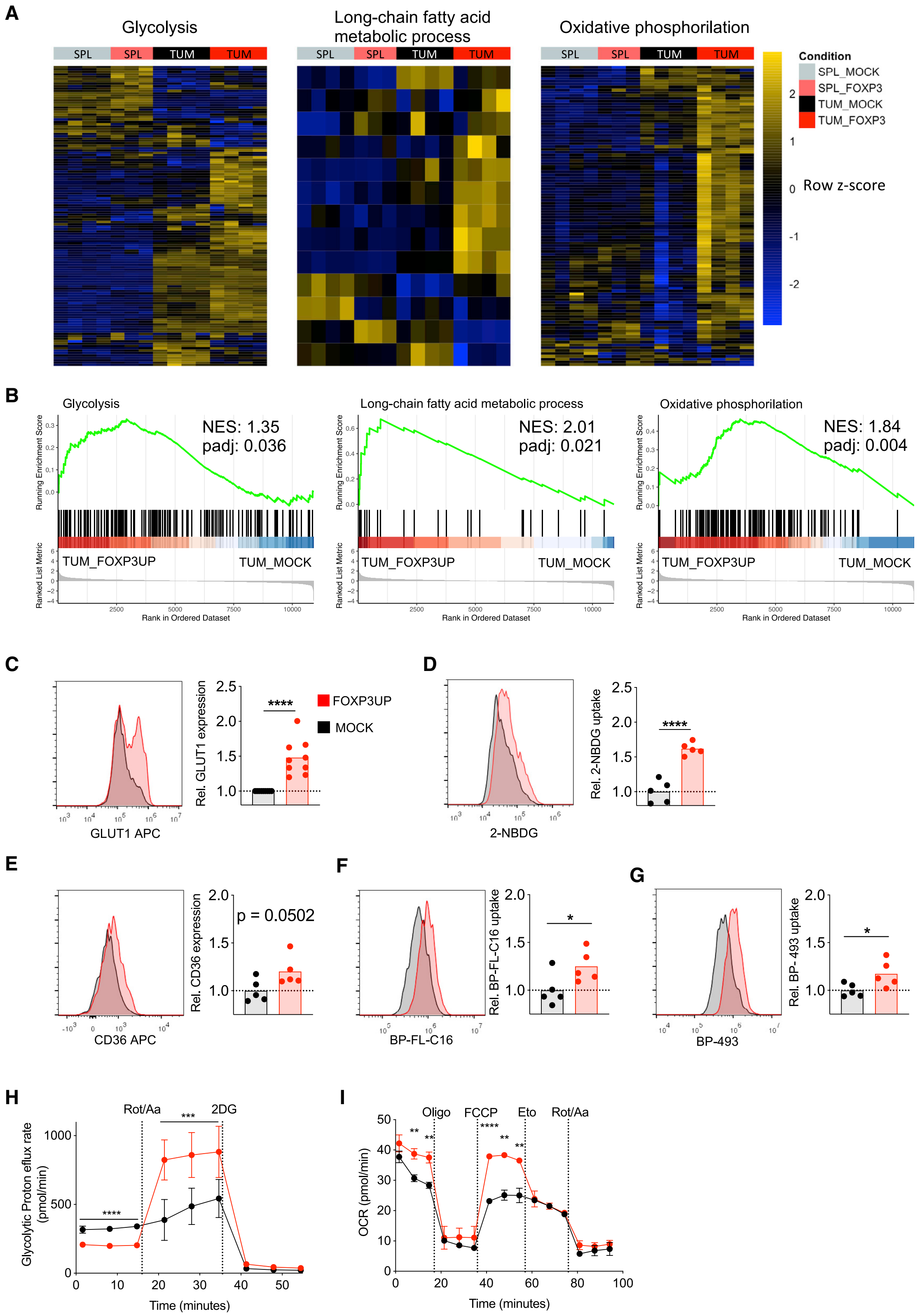

(legend on next page)

coincided with a reduced expression of the target antigen ovalbumin (OVA) in the tumor (Figure S8E), suggesting that Foxp3UP OT-I cells had more efficiently eliminated OVA-expressing tumors. The loss of the target antigen, and the state of differentiation/exhaustion of transferred cells, may explain why tumors finally escaped the action of Foxp3UP CD8 T cells.

FOXP3 overexpression in CD8 T cells allowed them to tailor their metabolism according to energy demand and nutrient availability

Gene sets related to glycolysis, lipid metabolism, and OXPHOS were positively enriched in Foxp3UP CD8 T cells as compared with mock CD8 T cells in tumor but not in the spleen (Figures 6A and 6B), suggesting that in the TME these cells had a higher capacity to engage different metabolic pathways. Interestingly, Foxp3UP CD8 TILs expressed higher levels of GLUT1 (Figure 6C) and were more efficient in taking up glucose (Figure 6D). Moreover, expression of CD36 (FA translocator) was borderline significantly higher in Foxp3UP CD8 TILs (Figure 6E), and these cells exhibited an enhanced FA uptake capacity (Figure 6F). Extracellular FA is stored by TEF cells in lipid droplets, which play a critical role in energy storage.[41] Interestingly, Foxp3UP CD8 TILs also showed higher lipid accumulation Figure 6G).

We further assessed the capacity of CD8 T cells to use aerobic glycolysis upon inhibition of mitochondrial ATP production by measuring the glycolytic proton efflux rate (GlycoPER), which correlates with lactate accumulation. When the mitochondria were operative, the glycolytic rate was slightly lower in Foxp3UP CD8 T cells than in mock CD8 T cells (Figure 6H). However, while inhibition of mitochondrial functions hardly affected the glycolytic rate of mock CD8 T cells, it markedly increased that of Foxp3UP CD8 T cells. This enhanced GlycoPER was fully abrogated by 2-deoxy-D-glucose (2-DG), indicating that it was dependent on glucose uptake. These data demonstrated an improved ability of Foxp3UP CD8 T cells to compensate for energy production through glycolysis after blockage of mitochondrial ATP production. The ability of T cells to use FAO to fuel OXPHOS allows them to survive when short of glucose.[42] To determine the effect of FOXP3 on the metabolic adaptation capacity of CD8 T cells, the oxygen consumption rate (OCR) and its regulation by FAO was monitored in CD8 T cells under starving conditions. As depicted in Figure 6I, the rate of baseline and maximal OXPHOS was higher in Foxp3UP CD8 T cells than in mock cells. Importantly, etomoxir (Eto), which is a specific inhibitor of FAO, substantially reduced the maximum OCR of Foxp3UP CD8 T cells to the levels exhibited by mock cells, suggesting that when nutrients are limiting, Foxp3UP CD8 T cells engaged FAO to drive OXPHOS.

FOXP3-overexpressing CD8 T cells exhibited enhanced proliferation under restricted metabolic conditions

Glycolysis supports the energy demand necessary for proliferation of TEF cells[43] and Tregs.[10] To determine the effect of glycolysis on the proliferation of Foxp3UP CD8 T cells, we restimulated them in serum-supplemented media with either normal or low glucose concentrations (2 and 0.3 g/L, respectively). Importantly, while both cell types proliferated similarly in the presence of normal glucose concentrations, Foxp3UP CD8 T cells did so more efficiently in the low-glucose medium (Figure 7A). Proliferation was affected by 2-DG but not by Eto (Figure 7A), and Foxp3UP CD8 T cells were similarly or somewhat more sensitive to 2-DG inhibition, especially in the low-glucose medium (Figure 7B). Interestingly, the effect of the OXPHOS inhibitor oligomycin (Omy) on proliferation varied according to the availability of glucose. While Omy hardly affected proliferation in the normal-glucose medium, it greatly inhibited CD8 T cell proliferation under glucose shortage (Figure 7B). These results suggested that when glucose was available, glucose oxidation to lactate was sufficient to generate the energy necessary to drive CD8 T cell proliferation. However, under glucose deprivation, glycolysis and OXPHOS must couple to support the energy demand necessary for proliferation of activated CD8 T cells. Strikingly, Foxp3UP CD8 T cells were more sensitive to Omy inhibition than mock CD8 T cells in the low-glucose medium, suggesting a more important role for OXPHOS in these cells (Figure 7B). In general, a similar picture was observed in hypoxia (Figure 7C) except that Eto, curiously, increased T cell proliferation when glucose was low.

Since serum itself contains a certain amount of glucose, we repeated the same experiment but with dialyzed serum-supplemented medium. Under these more extreme culture conditions, the differences between Foxp3UP and mock CD8 T cells were further exacerbated

Figure 6. FOXP3-overexpressing CD8 T cells exhibited improved glycolytic and lipidic metabolism

(A and B) Foxp3UP (GFP^+) and mock ($CD90.1^+$) OT-I ($CD45.1^+$) cells were co-injected at 1:1 ratio as described in Figure 2. Transcriptomic profile of Foxp3UP ($GFP^+CD45.1^+$) and mock ($CD90\text{-}1^+CD45.1^+$) CD8 T cells isolated from tumor and the spleen at day 5 of ACT. (A) Heatmap representation of hierarchical clustering of genes differentially expressed ($p < 0.05$) from hallmark glycolysis, KEGG long-chain FA metabolism, and OXPHOS gene set. (B) GSEA enrichment score curve of glycolysis, long-chain FA metabolism, and OXPHOS pathway in Foxp3UP versus mock TILs presented as the normalized enrichment score (NES). (C–G) Foxp3UP ($CD90.1^+$) and mock ($CD90.1^+$) OT-I ($CD45.1^+$) cells were separately injected into B16OVA tumor-bearing BL6 ($CD45.2^+$) mice, and $CD90.1^+CD45.1^+$ TILs were analyzed at day 5 upon ACT. Representative histograms and graphs showing GLUT1 expression (C), *ex vivo* 2-NBDG uptake (D), CD36 expression (E), *ex vivo* uptake of palmitate analog Bodipy-FL-C16 (BP-FL-C16) (F), and intracellular lipid droplet staining with Bodipy-493 (BP-493) (G) of Foxp3UP and mock cells. Data are presented as relative MFI values (MFI of studied cells divided by the average MFI of mock CD8 T cells). (H) GlycoPER assay. Before the assay, Foxp3UP and mock CD8 T cells were restimulated *in vitro* (2 h) with soluble anti-IgG-crosslinked anti-CD3 mAb. GlycoPER was measured at baseline and following injections with rotenone/antimycin A (Rot/AA) and 2-DG. (I) OCR assay under starving conditions. Foxp3UP and mock CD8 T cells were preconditioned (overnight) in substrate-limited growth medium and maintained in poor-nutrient Seahorse medium throughout the Seahorse assay. OCR was measured at baseline and in response to Omy, FCCP, Eto, and Rot/AA. Data are presented as mean (C–G) and mean ± SD (H and I). Symbols represent individual mice (C–G). Statistical significance was determined using unpaired t test (C–I). ****$p < 0.00005$, ***$p < 0.0005$, **$p < 0.005$, *$p < 0.05$. Compiled data from four different experiments (A and B) or one experiment representative of two (C–G) or three (H and I) experiments are shown.

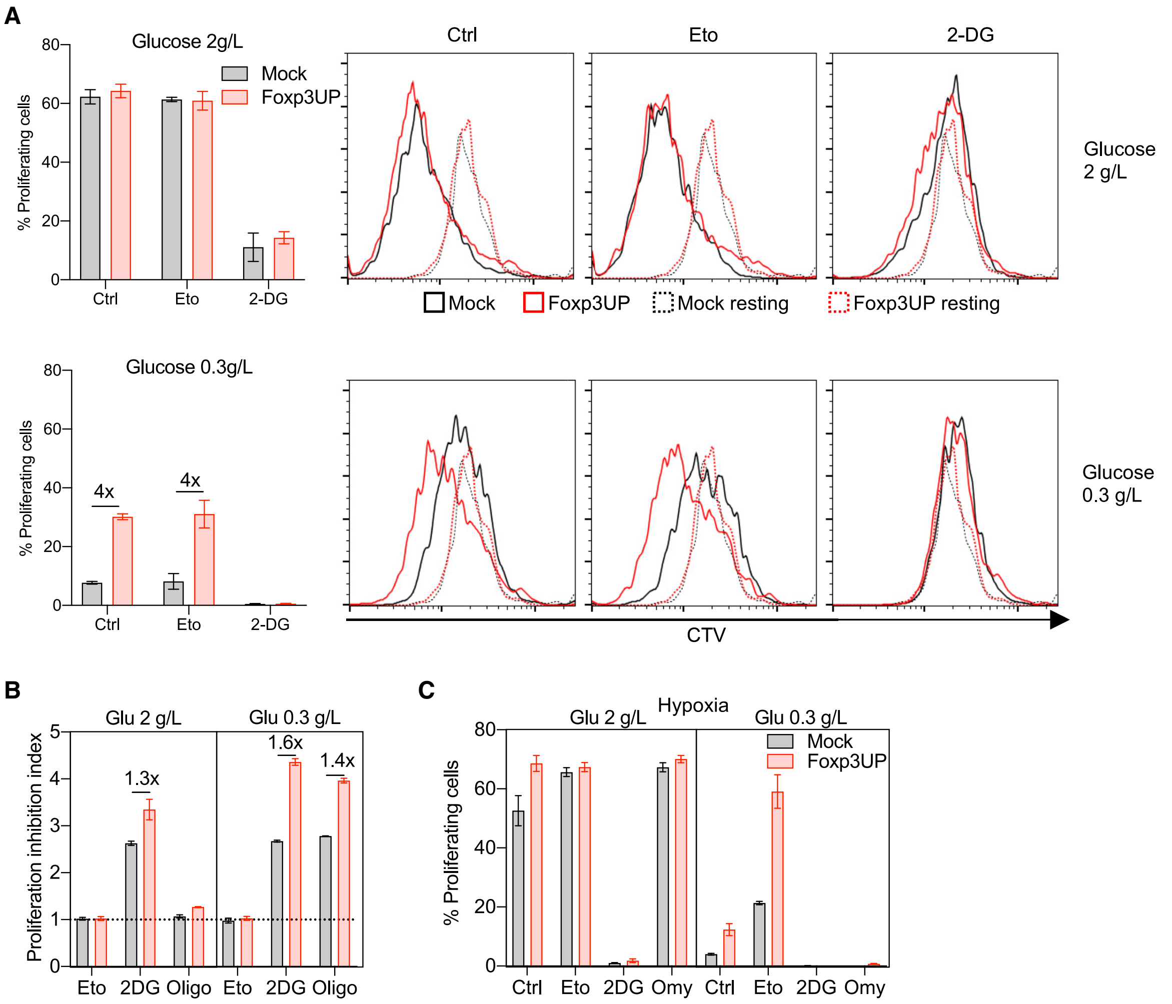

Figure 7. Advantage of FOXP3-overexpressing CD8 T cells in low-glucose conditions

(A and B) Eight-day *in vitro*-expanded Foxp3UP and mock (both GFP$^+$) CD8 T cells were labeled with CTV dye and restimulated *in vitro* (72 h) with plate-bound anti-CD3 mAb in IL-2- and glucose-free DMEM supplemented with normal or low glucose concentration and FCS in the presence or absence of 2-DG, Eto, or Omy. (A) Graphs showing the percentage of proliferating cells in transduced (GPF$^+$) cells and representative histograms of the CTV dilution assay. As reference, cells left without stimulation (Ctr) are shown (dotted histogram). (B) Proliferation inhibition index calculated as CTV MFI of inhibitor-treated cells/CTV MFI of inhibitor-free cells. (C) CTV-labeled cells cultured as in (A) and (B), but in hypoxia (1% O_2). Percentage of proliferating cells in transduced (GPF$^+$) cells. Data are presented as mean ± SD. One experiment was representative of two experiments (A–C).

(Figure S9). Notably, only Foxp3UP CD8 T cells were proliferating under conditions of low glucose and normoxia. In hypoxia, proliferation was only observed in the presence of normal glucose levels. 2-DG, but not Eto, inhibited the proliferation of both cell types under all conditions. Confirming our previous result in normoxia (Figure 7B), Omy only impaired proliferation when the glucose concentration was low (Figure S9). More importantly, Omy totally abrogated Foxp3UP cell proliferation in normoxia and low glucose, highlighting the importance of OXPHOS in driving proliferation when glucose was deficient. Strikingly, using dialyzed serum-supplemented medium, proliferation inhibition by Omy was also evident in the presence of normal glucose levels and hypoxia. Taken together, these results indicate that enforced FOXP3 expression enhanced CD8 T cell proliferation under glucose deprivation, likely by more efficient glucose usage and the coupling of glycolysis and OXPHOS.

DISCUSSION

In this study, we show that FOXP3 overexpression in mature CD8 T cells improved their therapeutic efficacy in ACT. Importantly, transcriptomic analysis of Foxp3UP CD8 TILs showed positive enrichment in a wide variety of metabolic pathways, such as glycolysis, FA metabolism, adipogenesis, and OXPHOS. This metabolic gene signature resembles that of tumor-infiltrating CD4 Tregs.[11] FACS analyses confirmed that Foxp3UP CD8 TILs exhibited an enhanced capacity for glucose and FA uptake as well as accumulation of intracellular lipids. Metabolic studies demonstrated that Foxp3UP CD8

T cells had an improved ability to compensate for the loss of mitochondrial ATP production through the activation of glycolysis. Moreover, in limiting nutrient conditions, these cells were able to engage FAO to fuel OXPHOS to meet their energy demands. The ability of Foxp3UP CD8 T cells to employ both glycolysis and FAO provides them with flexibility in fuel choice within the tumor, which may account for their enhanced antitumor properties. This is consistent with previous research showing that increasing FAO in CD8 T cells enhances their antitumor efficacy.[44,45]

FOXP3 expression in CD8 T cells favored their accumulation within the tumor. Interestingly, Foxp3UP CD8 T cells proliferated more than their mock counterparts in the tumor but not in the spleen. Notably, under extreme culture conditions that severely affect T cell proliferation (such as low glucose and hypoxia, both common features of the TME), Foxp3UP CD8 T cells had a proliferative advantage, which was impaired in the presence of inhibitors of glycolysis and OXPHOS, suggesting that both metabolic pathways were contributing. This resembles CD4 Tregs during their proliferative and migratory phase, in which a substantial proportion of glucose is used for mitochondrial oxidation.[12,46] Our findings suggest that a more efficient glucose usage and the ability to couple glycolysis and OXPHOS give Foxp3UP CD8 T cells a metabolic advantage that allows them to sustain their proliferation under metabolic restrictions, which would explain their proliferative superiority in the TME.

CCR4 mediates tumor migration of CD4 Tregs in response to CCL22,[36] highly expressed in melanoma.[47] Moreover, ectopic CCR4 expression in CD8 T cells enhances their tumor migration and therapeutic efficacy in ACT.[48] Remarkably, CCR4 expression was increased in Foxp3UP CD8 T cells, and in transwell assays these cells exhibited enhanced migration toward CCL22, which suggests that the CCL22/CCR4 axis may play a role in their tumor recruitment.[47] *Ccr3* is expressed by CD4 Tregs specifically among T cells.[49] It is known that hypoxia increases the production of CCR3 ligands[50] and that CD4 Tregs selectively accumulate in hypoxic areas.[51] Interestingly, *Ccr3* was the most expressed gene in Foxp3UP CD8 TILs, and CCR3 remained upregulated in these cells even in the late phase of ACT. These data suggest a role for the hypoxia-CCR3 axis in regulating the tumor migration of Foxp3UP CD8 T cells that warrants further study. Moreover, Foxp3UP CD8 TILs exhibited increased expression of tissue-remodeling-related genes (*Mmp9*) and cell-adhesion-related genes (*Itgb7*, *Itgae*, and *Itgam*) that may have facilitated their mobility through the tumor stroma.[52] Taken together, our data suggest that the increased intratumoral accumulation of Foxp3UP CD8 T cells may be due to their enhanced migration and proliferation within the tumors.

Importantly, Foxp3UP CD8 TILs exhibited increased GzmB levels. This was conditioned by TME-derived signals, since Foxp3UP and mock CD8 T cells did not differ in GzmB content either before or after transfer in the spleen or dLNs. Interestingly, hypoxia increased GzmB expression more markedly in Foxp3UP than in mock CD8 T cells. This finding, together with the positive enrichment of hypoxia response genes in Foxp3UP CD8 TILs, supports the idea that hypoxia-derived signals may have favored GzmB expression in these cells. However, Foxp3UP CD8 T cells exhibited impaired production of effector cytokines, such as IFNγ and TNFα, which could be explained in part by the suppressive action of FOXP3 on the *Ifng* locus.[53]

Foxp3UP CD8 T cells exhibited increased expression of the high-affinity IL-2 receptor (CD25), which may account for their high dependence on IL-2. It has been suggested that high CD25 levels may act as a sink absorbing IL-2 from the local environment, which has been proposed as one of the possible mechanisms of immunosuppression by CD4 Tregs.[54] Foxp3UP CD8 T cells also expressed the hallmark Treg suppressor gene *Il10*. However, the suppressive action of IL-10 is somewhat contradictory,[55] and more recently IL-10 has been shown to improve the expansion, cytotoxic functions, and therapeutic activity of CD8 TILs by promoting OXPHOS.[56] CD4 Tregs can also induce Tconv cell death via granzymes and perforins, or suppress Tconv priming by downregulating co-stimulatory molecules on dendritic cells via CTLA-4.[57] However, despite expressing several suppressive features of CD4 Tregs, Foxp3UP CD8 T cells did not exhibit suppressive activity.

The T cell differentiation status is very important for successful ACT. Although the presence of less-differentiated cells in the infused product guarantees long-term tumor control,[1] these cells must be able to differentiate into full TEF cells capable of killing tumor cells.[58] However, the nutrient restrictions in the TME constrain the T cell effector programs.[5,7] Interestingly, Foxp3UP CD8 TILs displayed a TEF cell gene signature within the tumor, but not before transfer, nor when isolated from the spleen, which suggests that they were capable of continuing their differentiation program in the tumor. The concurrence of the TEF cell gene signature with a diverse metabolic gene signature suggests that the capacity of Foxp3UP CD8 TILs to continue their differentiation program may be attributed to their capacity to metabolically adapt to the TME.

The presence of TRM T cells in tumors is associated with longer survival in various cancer types.[59] Interestingly, Foxp3UP CD8 TILs also exhibited a gene signature resembling that of previously described tumor-infiltrating TRM.[32] Phenotypic FACS studies confirmed the coexistence of less-differentiated cells expressing CD103 and more-differentiated cells expressing KLRG1 within Foxp3UP CD8 TILs. Divergent populations of $CD103^+$ CD8 TRM and $KLRG1^+$ CD8 TEF cells have also been described in the infiltrate of non-small cell lung cancer (NSCLC).[40] Interestingly, while $CD103^+$ cells dominated over $KLRG1^+$ cells in the early phase, a shift in favor of $KLRG1^+$ cells was observed in the late phase of ACT. This may be due to the conversion of $CD103^+$ cells into terminally differentiated TEF cells, although we cannot exclude the specific loss of $CD103^+$ cells. Furthermore, Foxp3UP CD8 T cells appeared to form a less-differentiated/memory-like cell compartment in dLNs at earlier times, which could have replenished the intratumoral Foxp3UP CD8 population and thus account for the increased number observed in late-stage tumors. Further studies are needed to determine the evolution of the different

cell subsets over time and between tissues. It is important to note that the enhanced ability of these cells to control tumor growth came at the expense of reaching a state of increased differentiation and exhaustion. This, together with the appearance of antigen-loss variants (due to the immunoediting effect of the ACT), would explain why tumors ultimately escaped the action of Foxp3UP CD8 T cells.

Here, we show that overexpression of FOXP3 in mature CD8 T cells improved their antitumor efficacy in ACT, favoring their metabolic adaptation to the TME as well as their recruitment, proliferation, and cytotoxic activity within tumors. However, in a recent publication using germline *Foxp3* mutated (scurfy) CD8 T cells, we showed that these cells exhibited superior antitumor activity than their wild-type counterparts.[60] The reasons for these seemingly opposing functions are not clear and require further study, but suggest that the role of FOXP3 in CD8 T cells depends on their stage of development and/or differentiation. Previous studies support our observations that FOXP3 enhances the effector functions of CD8 T cells within the tumors. Thus, in melanoma,[21] NSCLC,[22] and cervical cancer[23] patients, $CD8^{+}FOXP3^{+}$ cells were detected and described as early effector/effector memory cells. Moreover, elevated levels of $CD8^{+}FOXP3^{+}$ cells have been associated with response to PD-1 blockade in cervical cancer[23] patients and, more recently, in melanoma[24] patients. $CD8^{+}FOXP3^{+}$ TILs have also been associated with an effective antitumor response in mouse tumors.[21] However, exploiting FOXP3 overexpression to enhance the efficacy of CD8 T cells in ACT may also have downsides. On one hand, similar to challenges with expanding CD4 Tregs *in vitro*, which requires high doses of IL-2,[61] FOXP3 expression on CD8 T cells impaired their *in vitro* expansion. On the other hand, the ability of Foxp3UP CD8 T cells to continue their differentiation program in tumors might hamper the formation of long-term memory T cells. Enhanced manufacturing processes and strategies to favor the persistence of infused cells may be necessary for the clinical application of Foxp3UP CD8 T cells.

MATERIALS AND METHODS

Mice

OT-I (C57BL/6-Tg(TcraTcrb)1100Mjb/J), Pmel (B6.Cg-Thy1a/Cy Tg(TcraTcrb)8Rest/J), and CD45.1 (B6.SJL-$Ptprc^{a}Pep3^{b}$/BoyJ) mice were purchased from The Jackson Laboratory. OT-1 mice were crossed with CD45.1 mice to obtain homozygous OT-1 × CD45.1 mice. These strains were bred in our animal facility under specific pathogen-free conditions. C57BL/6 (BL6) mice were obtained from Harlan Laboratories. All animal handling and tumor experiments were approved by our institutional ethics committee (protocols 012-15 and 019-19) in accordance with Spanish regulations.

Cell lines and plasmids

The retroviral packaging cell line Platinum-E (PLATE) (American Type Culture Collection) was cultured in PLATE medium (DMEM-Glutamax, 10% fetal calf serum [FCS], 1% sodium pyruvate, 1% essential amino acids, 10 mM HEPES, 100 U/mL penicillin, 100 μg/mL streptomycin) supplemented with 1 μg/mL puromycin and 10 μg/mL blasticidin. The mouse melanoma cell line B16F10, B16F10 cells expressing OVA (B16OVA), and colon adenocarcinoma cell line MC38 were obtained from Dr. Melero (Center for Applied Medical Research, Spain). All tumor cell lines were verified by Idexx Radil. B16F10, B16OVA, MC38, and primary mouse T cells were cultured in complete medium (RPMI-1640-Glutamax, 10% FCS, 100 U/mL penicillin, 100 μg/mL streptomycin, 10 mg/mL gentamicin, 1 mM HEPES, 50 mM 2-mercaptoethanol). Cell lines were confirmed to be mycoplasma-free by using the MycoAlert Mycoplasma Detection Kit (Lonza).

Retroviral transduction of mouse CD8 T cells

RV production is described in supplemental methods. $CD8^{+}$ splenocytes were isolated from mouse spleens using the EasySep Mouse $CD8^{+}$ T cell Isolation Kit (STEMCELL Technologies) and activated in 24-well plates (Cellstar) coated with 2 μg/mL anti-CD3 (145-2C11) and 1 μg/mL soluble anti-CD28 (37.51) in complete medium containing 50 U/mL human IL-2 (Proleukin). At day 2 of activation, cells were resuspended in RV supplemented with 10 μg/mL protamine sulfate and 50 IU/mL human IL-2 and "spin-inoculated" at 2,000 × *g* for 90 min at 32°C. This was repeated with fresh RV supernatant the next day. Transduction efficiency was evaluated by measuring reporter protein expression (GFP, CD90.1) by FACS. For FOXP3 overexpression, CD8 T cells from OT-1 × CD45.1 and Pmel mice were genetically modified using MSCV-*Foxp3-Gfp* (or *Thy1.1*, coding for CD90.1) RV. CD8 T cells modified with empty vectors were designated as "mock." Cells were used at day 4 or day 7 of activation (day 2 or day 5 post transduction, respectively) (4-day or 7-day *in vitro*-expanded cells, respectively) or at the time indicated in the figures. Table S2 summarizes all the distinctive phenotypes of genetically modified CD8 T cells used in this study.

ACT experiments for antitumor efficacy

B16F10 or B16OVA mouse melanoma cells, which are responsive to the ACT of Pmel and OT1 TCR transgenic CD8 T cells, respectively, were used. Eight-week-old BL6 mice were subcutaneously implanted with 5×10^{5} tumor cells and, at day 7 or day 9 post tumor implantation, recipient mice were randomized and total body irradiated (TBI) with 3 Gy. At day 8 or day 10, therapy was initiated by intravenous (i.v.) injection of genetically modified CD8 T cells in combination with intraperitoneal administration of human IL-2 (Proleukin) (4×10^{4} U once daily for 4 consecutive days). The tumor growth rate was determined by blindly measuring the perpendicular diameters of tumors two or three times per week using digital calipers. The survival rate was also monitored. In line with ethics requirements, mice were sacrificed when they showed one or more of the following criteria: mean diameter of the tumor reaching 18 mm, ulcerated/necrotic tumor, and/or physical impairment (impaired mobility, signs of lethargy, lack of physical activity, and weight loss).[62] The number of euthanasia cases due to physical impairment was more frequent in tumor-bearing mice not treated with Pmel or OT-1 CD8 T cells.

ACT experiments for *ex vivo* CD8 T cell characterization assays

TBI, 10-day B16OVA-bearing BL6 mice received an i.v. injection of a 1:1 mix containing mock ($CD90.1^{+}$) and Foxp3UP (GFP^{+}) OT1 cells,

or mock ($GFP^+CD90.1^+$) and Foxp3DN ($GFP^+Cherry^+$) OT1 cells (8×10^6 total cell number/mouse). In addition, all mice received IL-2 systemically as already described. In some experiments, cell mixtures were labeled with CTV dye before injection as detailed in supplemental methods. In some experiments, genetically modified cells were separately injected. On the days indicated, mice were sacrificed and tumors, spleens, and dLNs were processed as described in supplemental methods. A single cell suspension was stained for FACS analysis as detailed below.

FACS and cell sorting

Tissue cell suspensions containing transferred CD8 T cells or *in vitro* cultured CD8 T cells were analyzed by FACS as described. The fluorophore-conjugated mAbs or protein used for FACS and cell sorting are specified in supplemental methods. Transferred CD8 T cells were distinguished from endogenous CD8 T cells by the surface marker CD45.1, while CD90.1 and GFP were used to identify and quantify each genetically modified CD8 T cell population. For FACS sorting, cells were surface-stained with mAbs against distinctive population markers (CD8, CD45.1, and/or CD90.1) in the presence of purified anti-CD16/32 mAb (Fc Block), which was followed by SYTOX Blue dead dye. Sorting of CD8 T cell subsets was performed with a FACSAria sorter (BD Biosciences) or a MoFloAstrio EQ (Beckman-Coulter). Aggregates and dead cells (SYTOX $Blue^+$) were excluded. Protocols used for surface and intracellular staining, annexin V labeling, glucose and FA uptake, and intracellular lipid droplet staining are detailed in supplemental methods. Cells were acquired using a FACS CANTO II (BD Biosciences) or Cytoflex (Beckman-Coulter). Absolute cell numbers were determined using a volumetric cytometer (Cytoflex). Data were analyzed with FlowJo software (Tree Star).

RNA analysis

For RNA-seq, cells from four independent experiments were analyzed. In brief, transduced cells isolated from the infusion cellular products (5×10^5) or transferred transduced cells isolated from tumor or spleen ($1–5 \times 10^5$) were processed and used to isolate RNA using a Maxwell 16 LEV simplyRNA tissue kit (Promega). A SMART-Seq v4 Ultra Low Input RNA Kit was used to generate cDNA, and the TruSeq RNA Sample Prep Kit v2 was used to generate RNA-seq libraries. Libraries were sequenced in a 150 bp paired-end run using NovaSeq6000 (Macrogen, Korea). FASTQ files were mapped to *Mus musculus* GRCm38.97 genome using STAR. From the resulting BAM files, raw gene counts were calculated using Subread (v.1.6.3). Next, differentially expressed genes were determined by DEseq2 (v.1.26.0) R package. Volcano plots and heatmaps were plotted using ggplot2 and heatmap R packages, respectively. GSEA was performed with clusterProfiler (v.3.14.3). The list of gene IDs with $\log_2$ fold changes from DEseq2 was used as dataset, and GO_BP, KEGG, WikiPathways, and MSigDB Hallmark collections were used as gene sets. GSEA bar plots were made using ggplot2 R package. RNA-seq raw data can be accessed at GEO: GSE206987. *Ova* and *Thy1.1* gene expression in tumor was determined by qRT-PCR using RNA isolated from total tumor, as described in supplemental methods.

Glycolytic rate assay

Two hours before the assay, CD8 T cells were stimulated with 2 μg/mL anti-CD3 mAb (145-2C11) crosslinked with 1 μg/mL anti-hamster immunoglobulin G (IgG) (MP) Ab in complete medium. They were then harvested, washed, and resuspended in Seahorse XF DMEM (pH 7.4) medium (Agilent) supplemented with 10 mM glucose, 1 mM sodium pyruvate, and 2 mM glutamine. Cells (10^5 cells/well) were plated onto Seahorse cell plates coated with Cell-Tak (Corning). The glycolytic rate test was performed by measuring the glycolytic proton efflux rate (GlycoPER), which correlates with lactate accumulation, in response to rotenone/antimycin A (0.5 μM each) and 2-DG (50 mM) (all from Sigma-Aldrich). Measurements were taken using a Seahorse XFp analyzer (Agilent).

Endogenous FA consumption assay

One day prior to the assay, CD8 T cells were cultured in substrate-limited growth medium (Seahorse XF DMEM [pH 7.4] medium [Agilent] supplemented with 0.5 mM glucose, 1 mM glutamine, 1% fetal bovine serum, 0.5 mM L-carnitine, and 5 U/mL human IL-2). On the day of the assay, cells were washed and resuspended in Seahorse XF DMEM medium supplemented with 2 mM glucose and 0.5 mM L-carnitine. Cells (10^5 cells/well) were then plated onto Cell-Tak-coated Seahorse cell plates. The OCR was measured in response to Omy (1.5 μM), carbonyl cyanide 4-(trifluoromethoxy) phenylhydrazone (FCCP; 1.5 μM) and rotenone/antimycin A (0.1 μM and 1 μM, respectively) (all from Sigma-Aldrich). Measurements were taken using a Seahorse XFp analyzer (Agilent).

In vitro proliferation in nutrient-limited medium

Genetically modified CD8 T cells were expanded for 7–10 days in complete medium containing 50 U/mL human IL-2. Cells were then labeled with 5 μM CTV dye and cultured for 72 h in 1 μg/mL anti-CD3 coated 96-well plates with high- or low-glucose medium (glucose-free RPMI medium supplemented with 10% FCS [Sigma], 50 μM 2-mercaptoethanol, 100 μg/mL penicillin/streptomycin, and 2 g/L or 0.3 g/L glucose, respectively). In some experiments, dialyzed FCS (One Shot; Invitrogen) instead of normal FCS was used. Specific metabolic inhibitors used were 2-DG (1 mM), Eto (50 μM), and Omy (3 nM) (all from Sigma). In some experiments, cells were also cultured under 1% O_2 atmosphere in an H35 Hypoxystation (Don Whitley) incubator. CTV dilution was assayed by FACS.

Assays to assess cytotoxic activity, chemotaxis, immune suppression, and sensitivity to restimulation-induced and cytokine-withdrawal-induced cell death

For cytotoxicity assays, genetically modified Pmel cells were co-cultured (12 h) with 5×10^4 B16OVA and MC38 tumor cells (test wells) in a 96-well plate at different effector cell to tumor cell (E:T) ratios. In some cases, tumor cells were previously pulsed with 2 μg/mL cognate Pmel peptide for 1 h and then washed extensively. As a control, tumor cells were cultured alone (control wells). Next, cells were stained with anti-CD45 mAb and 7AAD. The percentage of dead tumor cells ($7AAD^+CD45^-$) within total tumor cells ($CD45^-$) was analyzed by FACS. The percentage of specific lysis was calculated with the

formula: % specific lysis = ([% 7AAD$^+$ targets test wells] − [mean % 7AAD$^+$ targets control wells])/(100 − [mean % 7AAD$^+$ targets control wells]) × 100. The remaining assays are described in supplemental methods.

Statistical analysis

Statistical tests were performed using GraphPad Prism (v.8.4.0) or RStudio (v. 1.2.1335). For more details, see supplemental methods.

SUPPLEMENTAL INFORMATION

Supplemental information can be found online at https://doi.org/10.1016/j.ymthe.2022.08.017.

ACKNOWLEDGMENTS

The authors thank Dr. M.U. Latasa (CIMA) for her kind help with the hypoxic incubator and Dr. P.W. Miller (University of Navarra) and Dr. M. Hommel (CIMA) for English editing. Members of the animal facility and Flow Cytometry Unit at CIMA are also acknowledged. Work in the S.H.-S. laboratory was funded by ISCIII (PI18/00556 and PI21/00292) co-financed with “Fondos FEDER” ("Una manera de hacer Europa") and Gobierno de Navarra (Dpto. Industria) “Ayudas a Centros Tecnológicos y Organismos de Investigación” (GN2020 PC196-197, SOLIDET). J.J.L. was funded by Gobierno de Navarra, Ayudas para la realización de Proyectos Estratégicos de I+D 2019-2021 (0011-1411-2019-000079, DESCARTHeS), and Ministerio de Ciencia e Innovación (PID2019-108989RB-I00). E.C. and J.J.L. were funded by Fundación Ramón Areces.

AUTHOR CONTRIBUTIONS

Conceptualization, E.C., J.J.L., T.L., and S.H.-S.; methodology, E.C., N.C., U.M., E.E., R.C., E.V., J.R.R.-M., E.S., J.J.L., T.L., and S.H.-S.; formal analysis, E.C., N.C., and S.H.-S.; bioinformatic analysis, E.C. and J.R.R.-M.; investigation, E.C., E.V., U.M., E.E., R.C., and S.H.-S.; resources, J.R.R.-M., F.P., and J.J.L.; writing – original draft, E.C. and S.H.-S.; writing – review & editing, all authors; funding acquisition, J.J.L. and S.H.-S.

DECLARATION OF INTERESTS

The authors declare no competing interests.

REFERENCES

1. Gattinoni, L., Klebanoff, C.A., and Restifo, N.P. (2012). Paths to stemness: building the ultimate antitumour T cell. Nat. Rev. Cancer *12*, 671–684.
2. Fuertes Marraco, S.A., Neubert, N.J., Verdeil, G., and Speiser, D.E. (2015). Inhibitory receptors beyond T cell exhaustion. Front. Immunol. *6*, 310. https://doi.org/10.3389/fimmu.2015.00310.
3. Zhu, J., Powis De Tenbossche, C.G., Cané, S., Colau, D., Van Baren, N., Lurquin, C., et al. (2017). Resistance to cancer immunotherapy mediated by apoptosis of tumor-infiltrating lymphocytes. Nat. Commun. *8*, 1404. https://doi.org/10.1038/s41467-017-00784-1.
4. Horton, B.L., Williams, J.B., Cabanov, A., Spranger, S., and Gajewski, T.F. (2018). Intratumoral CD8+ T-cell apoptosis is a major component of T-cell dysfunction and impedes antitumor immunity. Cancer Immunol. Res. *6*, 14–24.
5. DePeaux, K., and Delgoffe, G.M. (2021). Metabolic barriers to cancer immunotherapy. Nat. Rev. Immunol. *21*, 785–797.
6. Cascone, T., McKenzie, J.A., Mbofung, R.M., Punt, S., Wang, Z., Xu, C., et al. (2018). Increased tumor glycolysis characterizes immune resistance to adoptive T cell therapy. Cell Metab. *27*, 977–987.
7. Vodnala, S.K., Eil, R., Kishton, R.J., Sukumar, M., Yamamoto, T.N., Ha, N.H., et al. (2019). T cell stemness and dysfunction in tumors are triggered by a common mechanism. Science *363*, eaau0135. https://doi.org/10.1126/science.aau0135.
8. Zheng, Y., Delgoffe, G.M., Meyer, C.F., Chan, W., and Powell, J.D. (2009). Anergic T cells are metabolically anergic. J. Immunol. *183*, 6095–6101.
9. Kempkes, R.W.M., Joosten, I., Koenen, H.J.P.M., and He, X. (2019). Metabolic pathways involved in regulatory T cell functionality. Front. Immunol. *10*, 2839. https://doi.org/10.3389/fimmu.2019.02839.
10. Pacella, I., and Piconese, S. (2019). Immunometabolic checkpoints of treg dynamics: adaptation to microenvironmental opportunities and challenges. Front. Immunol. *10*, 1889. https://doi.org/10.3389/fimmu.2019.01889.
11. Pacella, I., Procaccini, C., Focaccetti, C., Miacci, S., Timperi, E., Faicchia, D., et al. (2018). Fatty acid metabolism complements glycolysis in th selective regulatory t cell expansion during tumor growth. Proc. Natl. Acad. Sci. USA *115*, E6546–E6555.
12. Kishore, M., Cheung, K.C.P., Fu, H., Bonacina, F., Wang, G., Coe, D., et al. (2017). Regulatory T cell migration is dependent on glucokinase-mediated glycolysis. Immunity *47*, 875–889.
13. Howie, D., Cobbold, S.P., Adams, E., Bokum, A.T., Necula, A.S., et al. (2017). Foxp3 drives oxidative phosphorylation and protection from lipotoxicity. JCI Insight *2*, e89160. https://doi.org/10.1172/jci.insight.89160.
14. Miska, J., Lee-Chang, C., Rashidi, A., Muroski, M.E., Chang, A.L., Lopez-Rosas, A., et al. (2019). HIF-1α is a metabolic switch between glycolytic-driven migration and oxidative phosphorylation-driven immunosuppression of Tregs in glioblastoma. Cell Rep. *27*, 226–237.
15. Angelin, A., Gil-de-Gómez, L., Dahiya, S., Jiao, J., Guo, L., Levine, M.H., et al. (2017). Foxp3 reprograms T cell metabolism to function in low-glucose, high-lactate environments. Cell Metab. *25*, 1282–1293.
16. Watson, M.L.J., Vignali, P.D.A., Mullett, S.J., Overacre-Delgoffe, A.E., Peralta, R.M., Grebinoski, S., et al. (2021). Metabolic support of tumour-infiltrating regulatory T cells by lactic acid. Nature *591*, 645–651.
17. Chaput, N., Louafi, S., Bardier, A., Charlotte, F., Vaillant, J.C., Ménégaux, F., et al. (2009). Identification of CD8+CD25+Foxp 3+ suppressive T cells in colorectal cancer tissue. Gut *58*, 520–529.
18. Kiniwa, Y., Miyahara, Y., Wang, H.Y., Peng, W., Peng, G., Wheeler, T.M., et al. (2007). CD8+ Foxp3+ regulatory T cells mediate immunosuppression in prostate cancer. Clin. Cancer Res. *13*, 6947–6958.
19. Muthu Raja, K.R., Kubiczkova, L., Rihova, L., Piskacek, M., Vsianska, P., Hezova, R., et al. (2012). Functionally suppressive CD8 T regulatory cells are increased in patients with multiple myeloma: a cause for immune impairment. PLoS One *7*, e49446. https://doi.org/10.1371/journal.pone.0049446.
20. Battaglia, A., Buzzonetti, A., Baranello, C., Ferrandina, G., Martinelli, E., Fanfani, F., et al. (2009). Metastatic tumour cells favour the generation of a tolerogenic milieu in tumour draining lymph node in patients with early cervical cancer. Cancer Immunol. Immunother. *58*, 1363–1373.
21. Anichini, A., Molla, A., Vegetti, C., Bersani, I., Zappasodi, R., Arienti, F., et al. (2010). Tumor-reactive CD8+ early effector T cells identified at tumor site in primary and metastatic melanoma. Cancer Res. *70*, 8378–8387.
22. Tassi, E., Grazia, G., Vegetti, C., Bersani, I., Bertolini, G., Molla, A., et al. (2017). Early effector T lymphocytes coexpress multiple inhibitory receptors in primary non-small cell lung cancer. Cancer Res. *77*, 851–861.
23. Heeren, A.M., Rotman, J., Stam, A.G.M., Pocorni, N., Gassama, A.A., Samuels, S., et al. (2019). Efficacy of PD-1 blockade in cervical cancer is related to a CD8+FoxP3+CD25+ T-cell subset with operational effector functions despite high immune checkpoint levels. J. Immunother. Cancer *7*, 43. https://doi.org/10.1186/s40425-019-0526-z.
24. Berry, S., Giraldo, N.A., Green, B.F., Cottrell, T.R., Stein, J.E., Engle, E.L., et al. (2021). Analysis of multispectral imaging with the AstroPath platform informs efficacy of PD-1 blockade. Science *372*, eaba2609. https://doi.org/10.1126/science.aba2609.

25. Gavin, M.A., Torgerson, T.R., Houston, E., DeRoos, P., Ho, W.Y., Stray-Pedersen, A., et al. (2006). Single-cell analysis of normal and FOXP3-mutant human T cells: FOXP3 expression without regulatory T cell development. Proc. Natl. Acad. Sci. USA *103*, 6659–6664.

26. Allan, S.E., Crome, S.Q., Crellin, N.K., Passerini, L., Steiner, T.S., Bacchetta, R., et al. (2007). Activation-induced FOXP3 in human T effector cells does not suppress proliferation or cytokine production. Int. Immunol. *19*, 345–354.

27. Hill, J.A., Feuerer, M., Tash, K., Haxhinasto, S., Perez, J., Melamed, R., et al. (2007). Foxp3 transcription-factor-dependent and -independent regulation of the regulatory T cell transcriptional signature. Immunity *27*, 786–800.

28. van der Veeken, J., Campbell, C., Pritykin, Y., Schizas, M., Verter, J., Hu, W., et al. (2022). Genetic tracing reveals transcription factor Foxp3-dependent and Foxp3-independent functionality of peripherally induced Treg cells. Immunity *55*, 1173–1184.

29. Mayer, C.T., Floess, S., Baru, A.M., Lahl, K., Huehn, J., and Sparwasser, T. (2011). CD8+Foxp3+ T cells share developmental and phenotypic features with classical CD4+Foxp3+ regulatory T cells but lack potent suppressive activity. Eur. J. Immunol. *41*, 716–725.

30. Levings, M.K., Sangregorio, R., and Roncarolo, M.G. (2001). Human CD25+CD4+ T regulatory cells suppress naive and memory T cell proliferation and can be expanded in vitro without loss of function. J. Exp. Med. *193*, 1295–1301.

31. Gattinoni, L., Klebanoff, C.A., Palmer, D.C., Wrzesinski, C., Kerstann, K., Yu, Z., Finkelstein, S.E., et al. (2005). Acquisition of full effector function in vitro paradoxically impairs the in vivo antitumor efficacy of adoptively transferred CD8+ T cells. J. Clin. Invest. *115*, 1616–1626.

32. Duhen, T., Duhen, R., Montler, R., Moses, J., Moudgil, T., De Miranda, N.F., et al. (2018). Co-expression of CD39 and CD103 identifies tumor-reactive CD8 T cells in human solid tumors. Nat. Commun. *9*, 2724. https://doi.org/10.1038/s41467-018-05072-0.

33. Zeh, H.J., Perry-Lalley, D., Dudley, M.E., Rosenberg, S.A., and Yang, J.C. (1999). High avidity CTLs for two self-antigens demonstrate superior in vitro and in vivo antitumor efficacy. J. Immunol. *162*, 989–994.

34. Arnold, R., Brenner, D., Becker, M., Frey, C.R., and Krammer, P.H. (2006). How T lymphocytes switch between life and death. Eur. J. Immunol. *36*, 1654–1658.

35. Strasser, A., and Pellegrini, M. (2004). T-lymphocyte death during shutdown of an immune response. Trends Immunol. *25*, 610–615.

36. Marshall, L.A., Marubayashi, S., Jorapur, A., Jacobson, S., Zibinsky, M., Robles, O., et al. (2020). Tumors establish resistance to immunotherapy by regulating T reg recruitment via CCR4. J. Immunother. Cancer *8*, e000764. https://doi.org/10.1136/jitc-2020-000764.

37. Korbecki, J., Kojder, K., Simińska, D., Bohatyrewicz, R., Gutowska, I., Chlubek, D., et al. (2020). Cc chemokines in a tumor: a review of pro-cancer and anti-cancer properties of the ligands of receptors ccr1, ccr2, ccr3, and ccr4. Int. J. Mol. Sci. *21*, 1–29.

38. Doedens, A.L., Phan, A.T., Stradner, M.H., Fujimoto, J.K., Nguyen, J.V., Yang, E., et al. (2013). Hypoxia-inducible factors enhance the effector responses of CD8+ T cells to persistent antigen. Nat. Immunol. *14*, 1173–1182.

39. Martin, M.D., and Badovinac, V.P. (2018). Defining memory CD8 T cell. Front. Immunol. *9*, 2692. https://doi.org/10.3389/fimmu.2018.02692.

40. Corgnac, S., Malenica, I., Mezquita, L., Auclin, E., Voilin, E., Kacher, J., et al. (2020). CD103+CD8+ TRM cells accumulate in tumors of anti-PD-1-responder lung cancer patients and are tumor-reactive lymphocytes enriched with Tc17. Cell Rep. Med *1*, 100127. https://doi.org/10.1016/j.xcrm.2020.100127.

41. Welte, M.A., and Gould, A.P. (2017). Lipid droplet functions beyond energy storage. Biochim. Biophys. Acta Mol. Cell Biol. Lipids *1862*, 1260–1272.

42. Ecker, C., Guo, L., Voicu, S., Gil-de-Gómez, L., Medvec, A., Cortina, L., et al. (2018). Differential reliance on lipid metabolism as a salvage pathway underlies functional differences of T cell subsets in poor nutrient environments. Cell Rep. *23*, 741–755.

43. Menk, A.V., Scharping, N.E., Moreci, R.S., Zeng, X., Guy, C., Salvatore, S., et al. (2018). Early TCR signaling induces rapid aerobic glycolysis enabling distinct acute T cell effector functions. Cell Rep. *22*, 1509–1521.

44. Chowdhury, P.S., Chamoto, K., Kumar, A., and Honjo, T. (2018). PPAR-induced fatty acid oxidation in T cells increases the number of tumor-reactive CD8+ T cells and facilitates anti–PD-1 therapy. Cancer Immunol. Res. *6*, 1375–1387.

45. Zhang, Y., Kurupati, R., Liu, L., Zhou, X.Y., Zhang, G., Hudaihed, A., et al. (2017). Enhancing CD8+ T cell fatty acid catabolism within a metabolically challenging tumor microenvironment increases the efficacy of melanoma immunotherapy. Cancer Cell *32*, 377–391.

46. Procaccini, C., De Rosa, V., Galgani, M., Abanni, L., Calì, G., Porcellini, A., et al. (2010). An oscillatory switch in mTOR kinase activity sets regulatory T cell responsiveness. Immunity *33*, 929–941.

47. Matsuo, K., Itoh, T., Koyama, A., Imamura, R., Kawai, S., Nishiwaki, K., et al. (2016). CCR4 is critically involved in effective antitumor immunity in mice bearing intradermal B16 melanoma. Cancer Lett. *378*, 16–22.

48. Rapp, M., Grassmann, S., Chaloupka, M., Layritz, P., Kruger, S., Ormanns, S., et al. (2016). C-C chemokine receptor type-4 transduction of T cells enhances interaction with dendritic cells, tumor infiltration and therapeutic efficacy of adoptive T cell transfer. Oncoimmunology *5*. e1105428. https://doi.org/10.1080/2162402X.2015.1105428.

49. Ferhat, M., Hablot, J., Taieb, M., Salem, F., Netter, P., Peyrin-Biroulet, L., et al. (2021). Lack of protective effect of CCR3 blockade during experimental colitis may be related to CCR3 expression by colonic Tregs. Clin. Transl. Med. *11*, e455. https://doi.org/10.1002/ctm2.455.

50. Korbecki, J., Kojder, K., Barczak, K., Simińska, D., Gutowska, I., Chlubek, D., et al. (2020). Hypoxia alters the expression of CC chemokines and cc chemokine receptors in a tumor–a literature review. Int. J. Mol. Sci. *21*, 1–32.

51. McNamee, E.N., Korns Johnson, D., Homann, D., and Clambey, E.T. (2013). Hypoxia and hypoxia-inducible factors as regulators of T cell development, differentiation, and function. Immunol. Res. *55*, 58–70.

52. Arina, A., Beckett, M., Fernandez, C., Zheng, W., Pitroda, S., Chmura, S.J., et al. (2019). Tumor-reprogrammed resident T cells resist radiation to control tumors. Nat. Commun. *10*, 3959. https://doi.org/10.1038/s41467-019-11906-2.

53. Chen, C., Rowell, E.A., Thomas, R.M., Hancock, W.W., and Wells, A.D. (2006). Transcriptional regulation by Foxp3 is associated with direct promoter occupancy and modulation of histone acetylation. J. Biol. Chem. *281*, 36828–36834.

54. Vignali, D.A.A. (2012). Mechanisms of Treg suppression: still a long way to go. Front. Immunol. *3*, 191. https://doi.org/10.3389/fimmu.2012.00191.

55. Oft, M. (2014). IL-10: master switch from tumor-promoting inflammation to antitumor immunity. Cancer Immunol. Res. *2*, 194–199.

56. Guo, Y., Xie, Y.Q., Gao, M., Zhao, Y., Franco, F., Wenes, M., et al. (2021). Metabolic reprogramming of terminally exhausted CD8+ T cells by IL-10 enhances anti-tumor immunity. Nat. Immunol. *22*, 746–756.

57. Schmidt, A., Oberle, N., and Krammer, P.H. (2012). Molecular mechanisms of treg-mediated T cell suppression. Front. Immunol. *3*, 51. https://doi.org/10.3389/fimmu.2012.00051.

58. Wu, R., Forget, M.A., Chacon, J., Bernatchez, C., Haymaker, C., Chen, J.Q., et al. (2012). Adoptive T-cell therapy using autologous tumor-infiltrating lymphocytes for metastatic melanoma: current status and future outlook. Cancer J. *18*, 160–175.

59. Okla, K., Farber, D.L., and Zou, W. (2021). Tissue-resident memory T cells in tumor immunity and immunotherapy. J. Exp. Med. *218*. e20201605. https://doi.org/10.1084/jem.20201605.

60. Lozano, T., Conde, E., Martín-Otal, C., Navarro, F., Lasarte-Cia, A., Nasrallah, R., et al. (2022). TCR-induced FOXP3 expression by CD8+ T cells impairs their anti-tumor activity. Cancer Lett. *528*, 45–58.

61. Vimond, N., Lasselin, J., Anegon, I., Guillonneau, C., and Bézie, S. (2021). Genetic engineering of human and mouse CD4+ and CD8+ Tregs using lentiviral vectors encoding chimeric antigen receptors. Mol. Ther. Methods Clin. Dev. *20*, 69–85.

62. Voltarelli, F.A., Frajacomo, F.T., de Souza Padilha, C., Testa, M.T.J., Cella, P.S., Ribeiro, D.F., et al. (2017). Syngeneic B16F10 melanoma causes cachexia and impaired skeletal muscle strength and locomotor activity in mice. Front. Physiol. *8*, 715. https://doi.org/10.3389/fphys.2017.00715.

YMTHE, Volume *31*

Supplemental Information

FOXP3 expression diversifies the metabolic capacity and enhances the efficacy of CD8 T cells in adoptive immunotherapy of melanoma

Enrique Conde, Noelia Casares, Uxua Mancheño, Edurne Elizalde, Enric Vercher, Roberto Capozzi, Eva Santamaria, Juan R. Rodriguez-Madoz, Felipe Prosper, Juan J. Lasarte, Teresa Lozano, and Sandra Hervas-Stubbs

SUPPLEMENTARY DATA

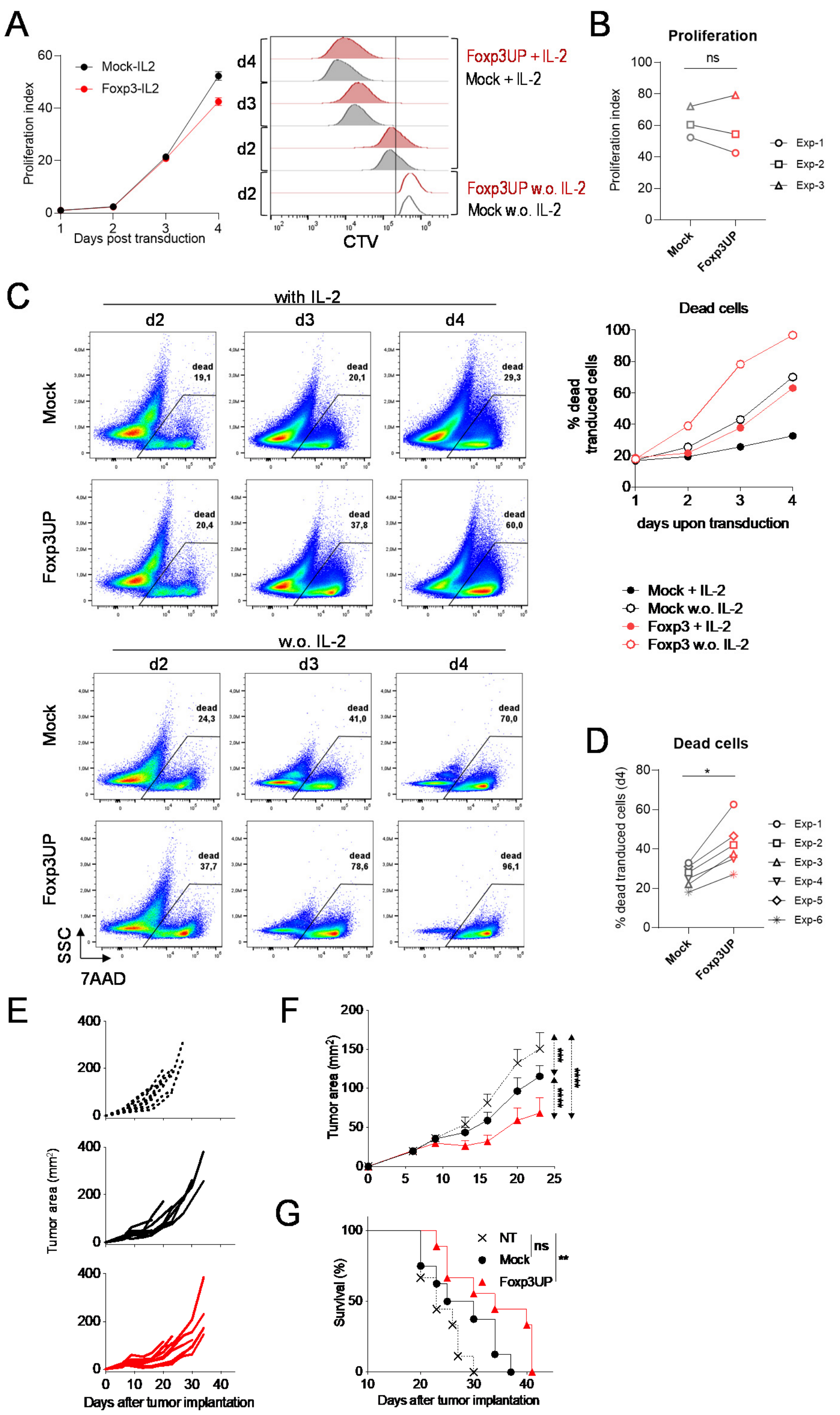

Figure S1. Effects of FOXP3 overexpression in CD8 T cells. (**A and B**) Proliferation rate of live transduced cells measured by CTV dilution assay. Magnetically sorted untouched CD8^{+} splenocytes were activated and 48 hours later they were labeled with CTV dye and infected with an empty RV or an RV encoding FOXP3 to generate Mock and Foxp3UP CD8 T cells, respectively. After transduction, cells were cultured with (+) or without (w.o.) IL-2 for 4 days. Cells were harvested and stained with anti-CD90.1 (transduced cell surrogate marker) mAb and 7AAD to assess proliferation. (**A**) The graph shows the proliferation index calculated as "Median Fluorescence Intensity (MFI) of cells cultured w.o. IL-2 corresponding to day 1 (average of replicates) / CTV of cells expanded with IL-2 at the day indicated". (Right) Overlay histograms showing CTV MFI of representative wells of Mock and Foxp3UP CD8 T cells expanded with or without IL2 at the days indicated. (**B**) Proliferation index of cells cultured with IL-2 (day 4 post infection). Compiled data from 3 independent experiments. (**C**) Percentage of dead (7AAD^{+}) cells within transduced (CD90.1^{+}) cells along the culture period. Dot plots show representative wells of each culture condition at the days indicated. (**D**) Percentage of dead cells within transduced cells at day 4 of culture. Compiled data from 6 independent experiments. (**E-G**) Six-day B16F10 tumor-bearing mice were treated with 7-day in vitro-expanded Foxp3UP or Mock Pmel cells (10^7/mouse; 7 mice/group). Tumor size (mm^2) from individual mice (**E**), average tumor size (**F**) and overall survival (**G**). Data are represented as mean±SD (A and C) and mean±SEM (F). Symbols represent individual mice (**F**) or experiments (**B and D**). Statistical significance was determined using paired t test (**B and D**), nonlinear regression (curve fit) (**F**), and Mantel-Cox test (**G**). ***$p < 0.0005$, **$p < 0.005$, *$p < 0.05$. One experiment representative of 6 (**C**), 3 (**A**) and 2 (**E-G**) is shown.

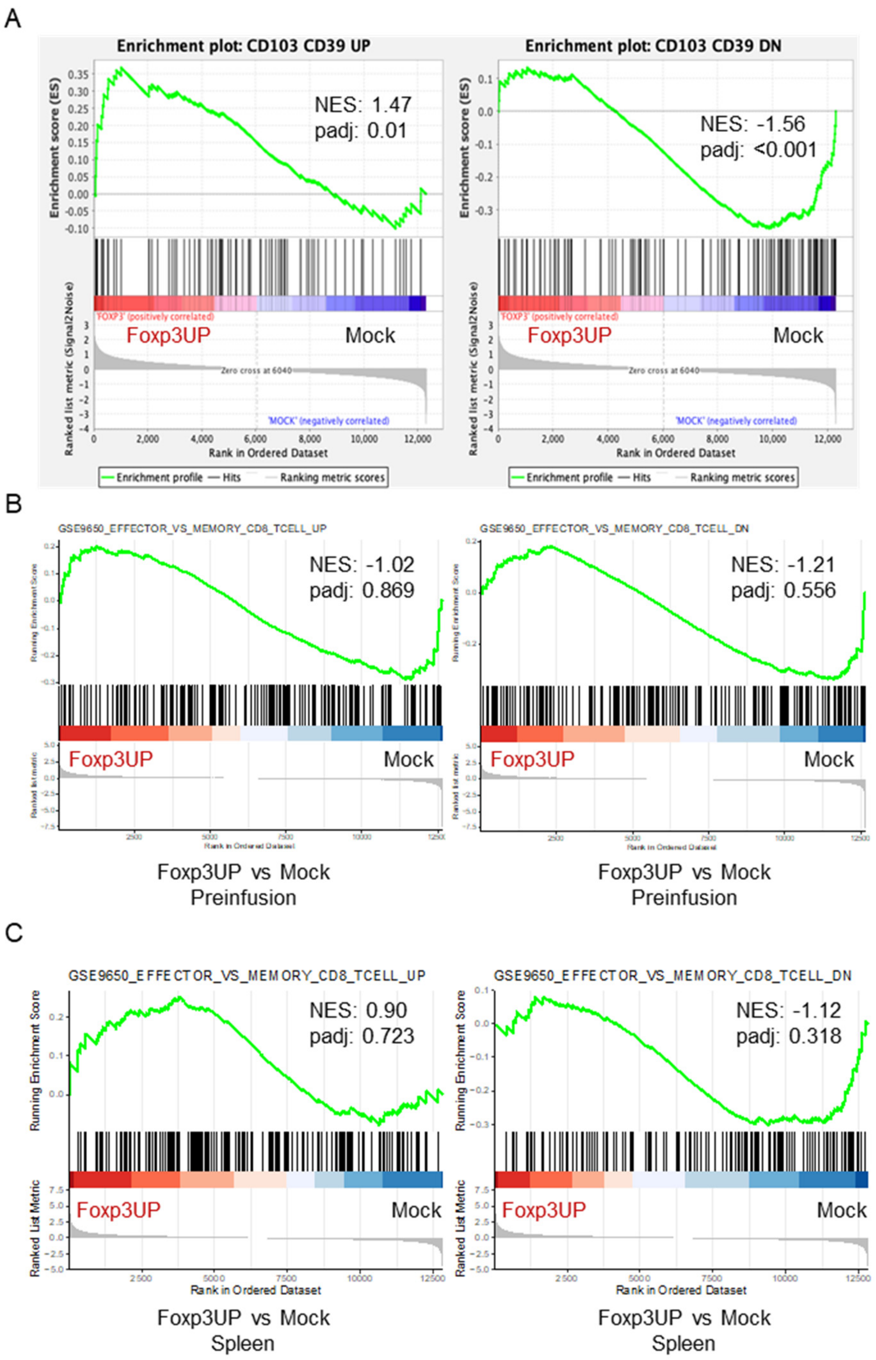

Figure S2. **Transcriptomic signatures of tumor-infiltrating Foxp3UP CD8 T cells** (**A**) GSEA of CD103^{+}CD39^{+} cell gene sets [upregulated (UP) and downregulated (DN) genes] obtained from GSE114944 in the Foxp3UP vs Mock TIL dataset. (**B and C**) In parallel to experiments shown in figure 2, RNAseq was performed in Foxp3UP and Mock OT-I cells prior to infusion (preinfusion) (**B**) and in those infiltrating the spleen after ACT (**C**). GSEA enrichment score curve of "Effector vs memory CD8+ " upregulated (UP) and downregulated (DN) gene sets in the transcriptome of Fox3UP vs Mock CD8 T cells presented as the normalized enrichment score (NES). (**A-C**) Compiled data from 4 different experiments.

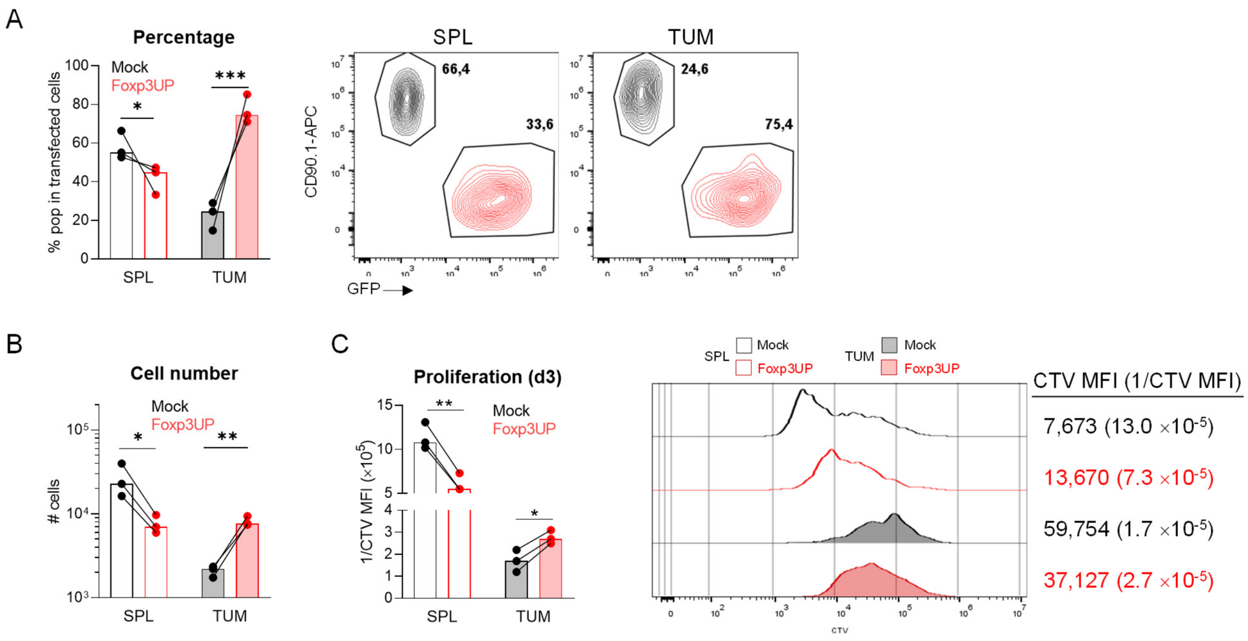

Figure S3. FOXP3 overexpression enhanced CD8 T cell proliferation in the TME. Second experiment representative of the one shown in figure 4A-E. OT-I cells were analyzed in tumor and spleen at day 3. (**A**) Graph (left) and representative dot plots show the percentage of Foxp3UP and Mock OT-I cells in total transduced (GPF$^+$ plus CD90.1$^+$) CD45.1$^+$ cells in tumor (TUM) and the spleen (SPL). (**B**) Total number of Foxp3UP and Mock CD8 T cells in tumor and the spleen. (**C**) (Left) Proliferation [depicted as reverse of CTV MFI (1/CTV MFI)] of Foxp3UP and Mock OT-I cells in tumor and spleen. (Right) Representative histogram. In this experiment, the number of Foxp3UP and Mock CD8 T cells per mg of tumor was not determined because tumor weight was not recorded. Data are represented as mean. Symbols represent individual mice. Statistical significance was determined using paired *t* test ***p < 0.0005, **p < 0.005, *p < 0.05.

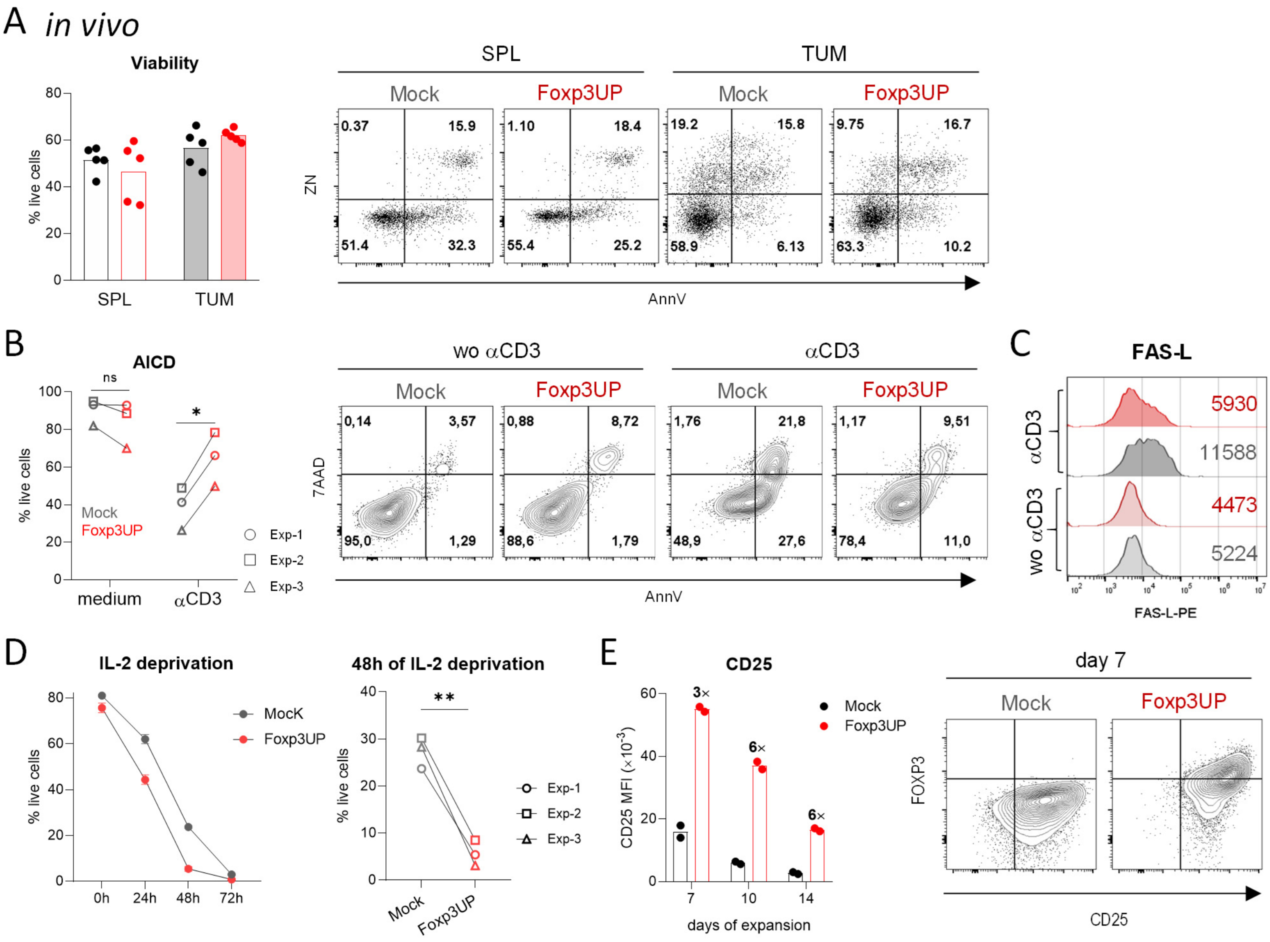

Figure S4. Effect of FOXP3 overexpression on CD8 T cell sensitivity to AICD and IL-2 deprivation. (A) *In vivo* viability of transferred cells. Since GFP expression is lost when cells die, *in vivo* viability assessment was performed using CD90.1 as the transduced cell surrogate marker. Foxp3UP ($CD90.1^+$) and Mock ($CD90.1^+$) OT1 ($CD45.1^+$) cells were separately injected into 10-day B16OVA tumor-bearing BL6 ($CD45.2^+$) mice. Five days later, cells from tumors and spleens were stained with Zombie NIR (ZN) and Annexin V (AnnV) and viability [% of viable (ZN^-AnnV^-) cells within transduced ($CD90.1^+CD45.1^+$) cells] was assessed by FACS. Bar plot and representative dot plots are shown. **(B)** AICD Assay. 4-day *in vitro*-expanded Mock ($CD90.1^+$) and Foxp3UP ($CD90.1^+$) CD8 T cells cleaned from dead cells were cultured in 96-well plates coated with or without (resting) anti-CD3 mAb (1µg/ml) in the absence of IL-2. After 24 hours of culture, samples were stained with 7AAD and AnnV and analyzed by FACS. (**B, left**) Percentage of live ($7AAD^-AnnV^-$) cells within transduced ($CD90.1^+$) cells. Compiled data from 3 independent experiments. (**B, right**) Representative dot plots from one experiment. **(C)** FASL surface expression depicted as MFI. Cells cultured as in **B** and stained with anti-FASL mAb, AnnV and 7AAD dye. Expression of FASL was analyzed in live transduced ($7AAD^-AnnV^-CD90.1^+$) cells by FACS. **(D)** IL-2 deprivation assay. Eight-day *in vitro*-expanded Mock ($CD90.1^+$) and Foxp3UP ($CD90.1^+$) CD8 T cells cleaned from dead cells were cultured in the absence of IL-2. The percentage of live ($7AAD^-AnnV^-$) cells of transduced ($CD90.1^+$) cells was analyzed at baseline and at different time of culture. **(D, left)** One representative experiment. (**D, right**) Percentage of live cells after 48h of IL-2 deprivation. Compiled data from 3 independent experiments. **(E)** CD25 surface expression depicted as MFI. $CD8^+$ splenocytes were activated (day 0) and 48 hours later they were transduced with Mock or FOXP3 RV. After transduction, cells were cultured with IL-2 up to day 14. Expression of CD25 was analyzed in live transduced ($7AAD^-CD90.1^+$) cells by FACS**.** Data are represented as mean (**A and E**), mean±SD (**D**). Symbols represent individual mice (**A**), independent experiments (**B and D left**) or experimental duplicates (**E, left**). Statistical significance was determined using two-way ANOVA for multiple comparisons (**A**) and paired t test (**B left and D right**). $**p < 0.005$, $*p < 0.05$. One experiment representative of 2 (**A and E**) and 3 (**C**) experiments is shown.

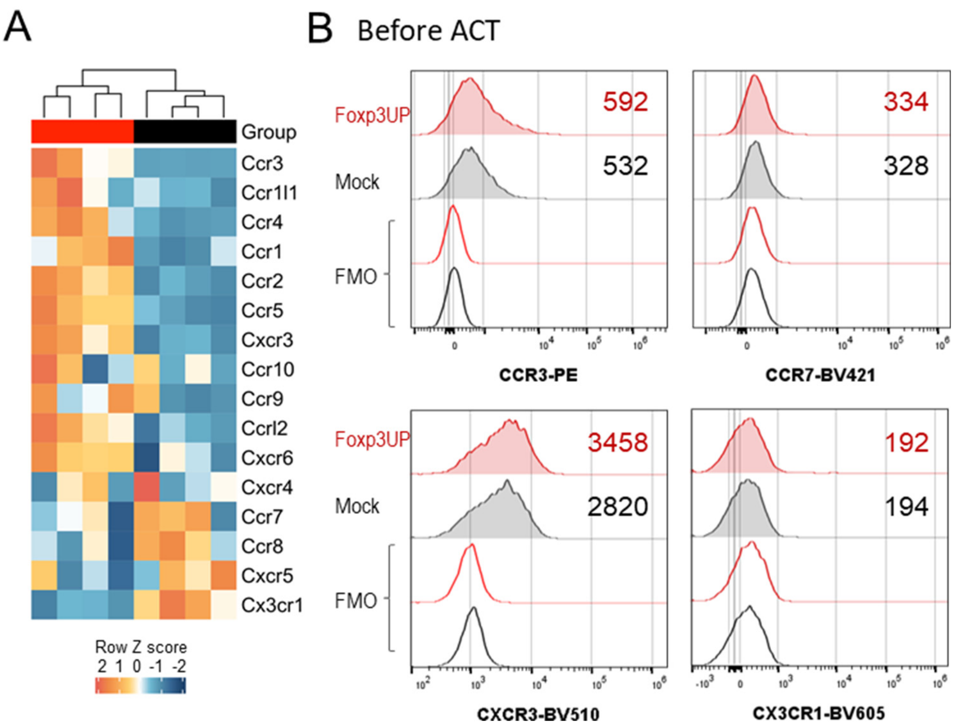

Figure S5. Effect of FOXP3 overexpression on the surface expression of chemokine receptors by CD8 T cells. (**A**) Gene expression heat map of chemokine receptors in Foxp3UP and Mock OT-I cells isolated form tumors (Data from RNAseq). (**B**) Expression of different chemokine receptors in 4-day *in vitro*-expanded Foxp3UP and Mock OT-I cells. Representative histograms are shown. As negative control, fluorescence minus one (FMO) is shown. Compiled data from 4 different experiments (**A**). One experiment representative of 2 experiments (**B**).

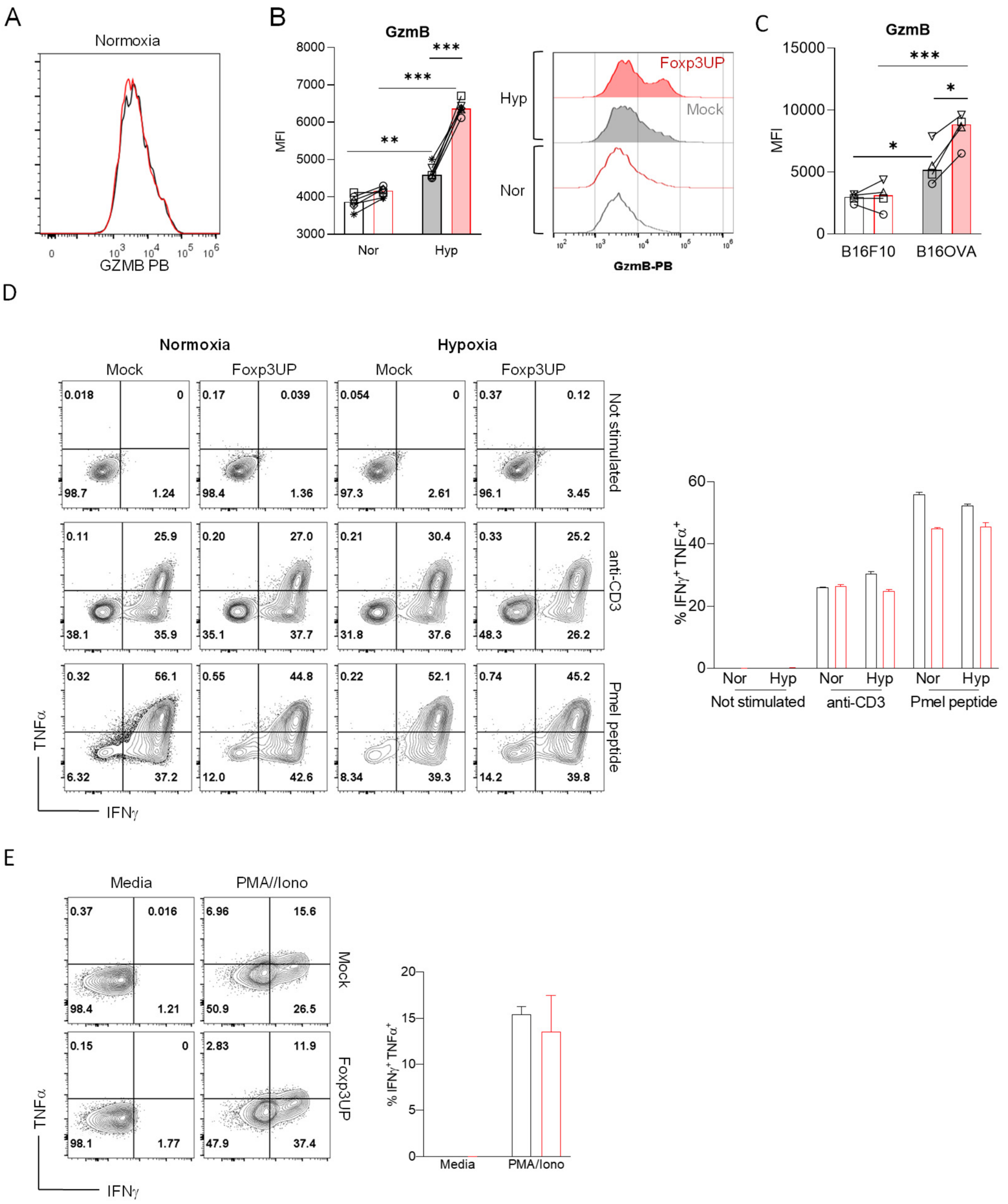

Figure S6. *In vitro*, Foxp3UP CD8 T cells exhibited enhanced granzyme B (GzmB) expression but diminished production of effector cytokines. (**A**) Overlay histograms showing GzmB MFI in Mock and Foxp3UP CD8 T cells before transfer. (**B and C**) Effect of hypoxia and tumor cells on the GzmB expression (depicted as MFI) in T cells. (**B**) Activated Mock (GFP^{+}) and Foxp3UP (GFP^{+}) OT-I cells were cultured *in vitro* for 48 hours under normoxic (20% O_2) or hypoxic (1% O_2) conditions. Graph shows compiled data from 6 different experiments. (**C**) OT-1 T cells were cultured (18 hours) with cognate (B16OVA) or non-cognate (B16F10) tumor cells at a T cell:tumor cell ratio of 5:1. Graph shows compiled data from 3 different experiments. (**D and E**) Cells were cultured in normoxic (**D and E**) or hypoxic (**D**) conditions as in **B**. At 48 hours of culture, OT-I cells were restimulated with OVA peptide (**D**) or PMA/Ionomycin (PMA/Iono) (**E**) and Brefeldin A. IFNγ and TNFα production was assessed 4 hours later by FACS. Representative FACS dot plots are depicted (**D and E**). (**D and E, right**) Percentage of $IFN\gamma^{+}TNF\alpha^{+}$ cells within Mock and Foxp3UP CD8 T cells. Data are represented as mean (**B and C**) and mean±SD (**D and E**). Symbols represent independent experiments (**B and C**). Statistical significance was determined using two-way ANOVA for multiple comparisons (**B and C**). ***$p < 0.0005$, **$p < 0.005$, *$p < 0.05$. One experiment representative of 2 (**D and E**), 4 (**A**) or 6 (**B, right**) experiments is shown.

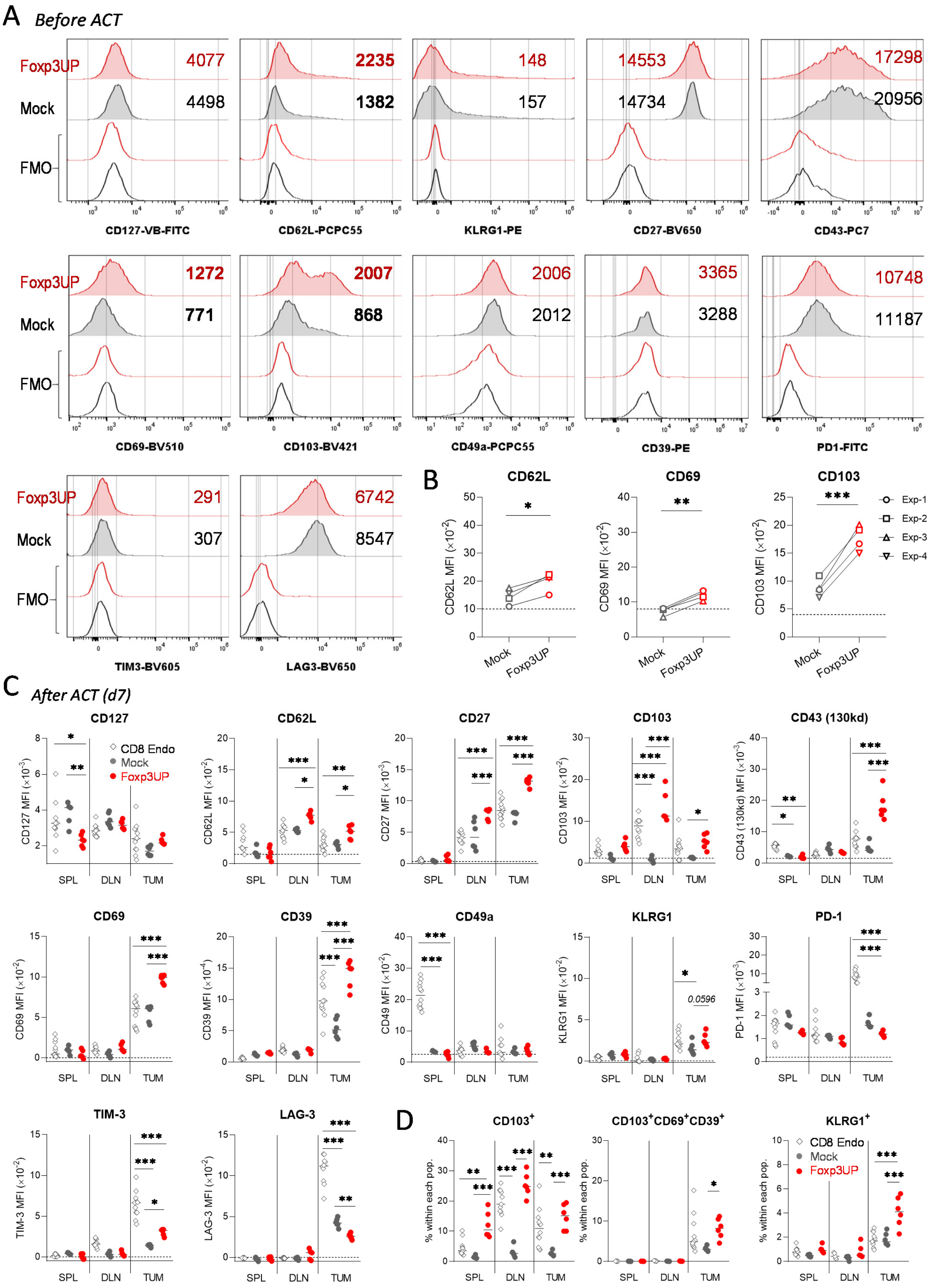

Figure S7. Continued on next page

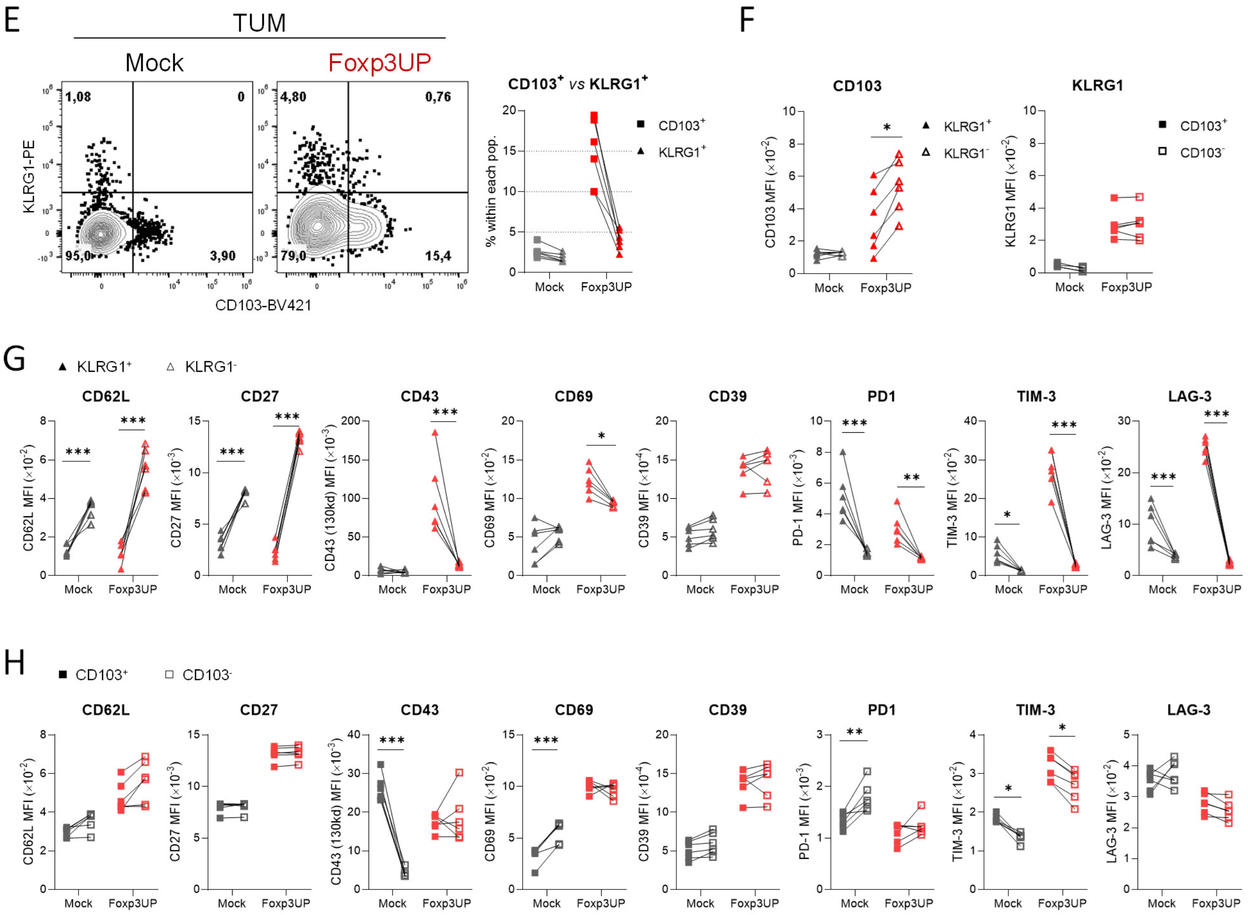

Figure S7. Effect of FOXP3 overexpression on the expression of different markers associated with differentiation, activation and exhaustion of CD8 T cells. (**A and B**) Expression of different surface markers in 4-day *in vitro*-expanded Foxp3UP and Mock OT-I cells. (**A**) Representative histograms. As negative control, FMO is shown. (**B**) MFI of CD62L, CD69 and CD103 in Foxp3UP and Mock CD8 T cells. Compiled data from 4 independent experiments. Dotted line indicates FMO values. (**C-H**) 4-day *in vitro*-expanded Foxp3UP and Mock OT1 cells were injected separately into 10-day B16OVA tumor-bearing BL6 mice (n = 6). The expression of different surface markers was assessed (FACS) in Foxp3UP and Mock CD8 T cells from spleen, dLN and tumors at day 7 of ACT. (**C**) MFI of assessed markers in Foxp3UP and Mock CD8 T cells. Dotted line indicates FMO values. (**D**) Percentage of $CD103^+$, $CD103^+CD69^+CD39^+$ and $KLRG1^+$ within each cell subset inndicated. (**C and D**) As reference, endogenous (endo) CD8 T cells are shown. (**E**) Percentage of $CD103^+$ cells vs $KLRG1^+$ cells within Mock and Foxp3UP CD8 TILs. (**F**) MFI of CD103 and KLRG1 marker on $KLRG1^+$ *vs* $KLRG1^-$ cells (left) and $CD103^+$ *vs* $CD103^-$ cells (right), respectively, within Foxp3UP and Mock OT-I TILs. (**G and H**) MFI of indicated markers in $KLRG1^+$ *vs* $KLRG1^-$ cells (**G**) and $CD103^+$ *vs* $CD103^-$ cells (**H**) within Foxp3UP and Mock OT-I TILs. Data are represented as median (**C**). Symbols represent independent experiments (**B**) or individual mice (**C-H**). Statistical significance was determined using paired *t* tests (**B**) and two-way ANOVA for multiple comparisons (**C-H**). (**C-H**) Only significances between relevant groups are shown. ***p < 0.0005, **p < 0.005, *p < 0.05. One experiment representative of 2 is shown (**A and C-H**).

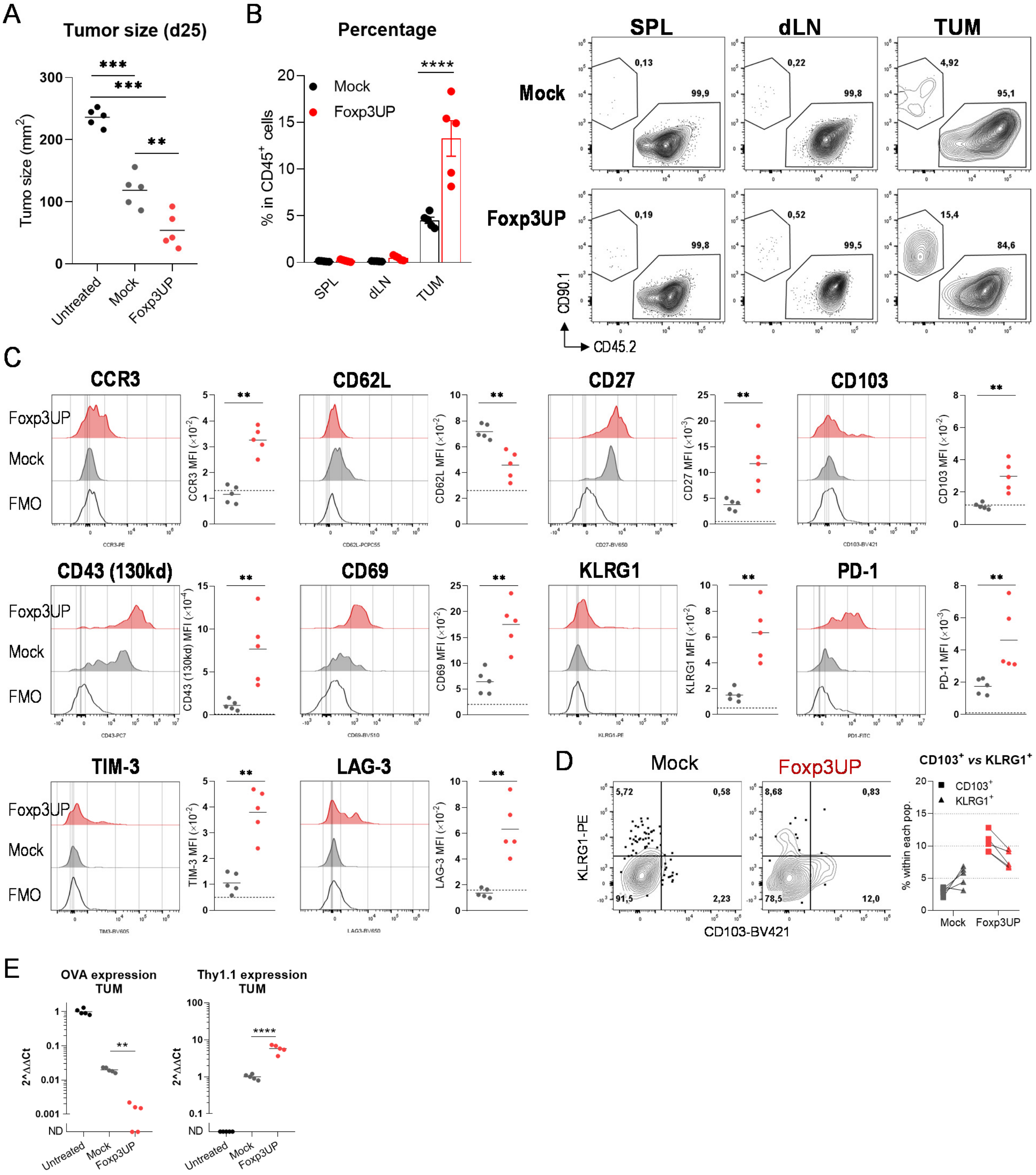

Figure S8. Foxp3UP CD8 T cells outnumbered Mock cells in tumors and exhibited a greater ability to control tumor growth in the late-phase of ACT at the expense of becoming exhausted. Foxp3UP (CD90.1$^+$) and Mock (CD90.1$^+$) OT1 (CD45.1$^+$) cells were separately injected into 10-day B16OVA-bearing BL6 (CD45.2$^+$) mice (10^6/mouse; 5 mice/group). As control, one group of mice was left untreated. Twenty-five days later, cells from spleens, dLNs and tumors were analyzed by FACS. (**A**) Tumor size (mm^2). (**B**) Percentage of transduced (CD90.1$^+$) cells within CD45$^+$ cells in treated mice. (Right) Representative dot plots. CD45.2$^+$ cells are endogenous cells. (**C**) The expression of different surface markers was assessed in Foxp3UP and Mock OT-I TILs. Representative histograms (left) and graphs (right) showing the MFIs for each marker. As negative control, FMO corresponding to Foxp3UP cells is included (empty grey histogram in the overlaid histograms and dotted line in the graphs). (**D**) OVA and Thy1.1 gene expression determined by quantitative RT-PCR using RNA isolated from total tumor. ND (not detected). Data are represented as mean (**A, C**-D) and mean+SEM (**B**). Symbols represent individual mice (**A-D**). Statistical significance was determined using unpaired *t* test (**C and D**) and two-way ANOVA for multiple comparisons (**A and B**). (**B**) Only significances between relevant groups are shown. ****p < 0.0001, ***p < 0.0005, **p < 0.005. One experiment representative of 2 experiments.

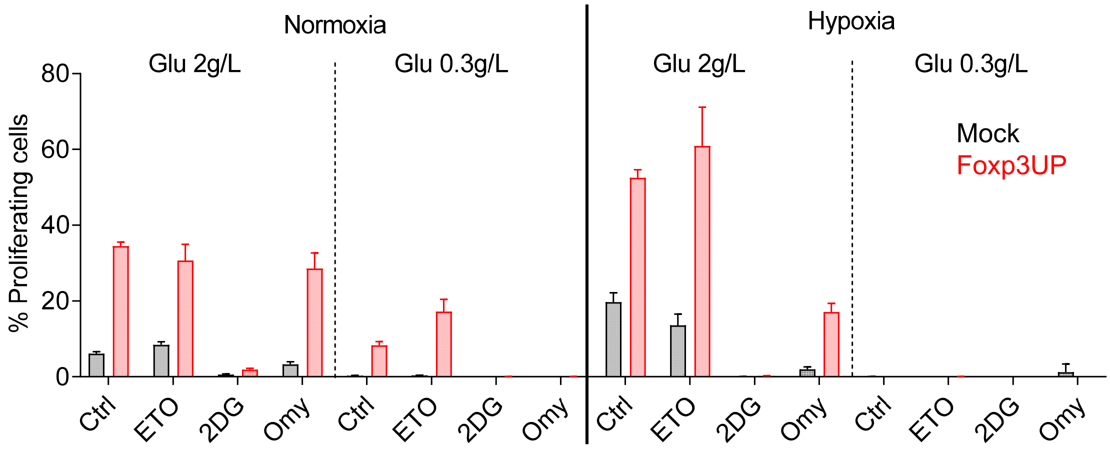

Figure S9. Proliferation of CD8 T cells in medium supplemented with low-glucose and dialyzed serum in the presence/absence or metabolic inhibitor under normoxic and hypoxic conditions. 8-day *in vitro*-activated Foxp3UP and Mock CD8 T cells (both GFP^+) were labeled with CTV dye and re-stimulated *in vitro* (72 hours) with plate-bound anti-CD3 mAb in glucose-free DMEM medium supplemented with normal or low glucose concentration (2 and 0.3 g/L, respectively), dialyzed fetal calf serum (FCS) in the presence or absence of 2-DG (1 mM), Eto (50 µM) or Oligo (3 nM), under normoxia (20% O_2) or hypoxia (1% O_2). The percentage of proliferating cells within transduced (GFP^+) cells is shown. Data are represented as mean±SD.

Table S1. GSEA of the comparison of Foxp3UP/Mock CD8 T cells before and after ACT in the spleen and tumor. GSEA by clusterProfiler illustrates gene sets significantly enriched in Foxp3UP/Mock cell comparison before and after ACT in the spleen and tumor. NES and padj in Foxp3UP/Mock cell comparison are shown. Those NES with a significant padj (padj < 0.05) are shown in bold. NA: not available

	BEFORE ACT		AFTER ACT			
			SPLEEN		TUMOR	
**Description**	**NES**	**padj**	**NES**	**padj**	**NES**	**padj**
Long-chain fatty acid metabolism	1.4198	*0.8117*	-1.0749	*0.8351*	**2.0133**	***0.0208***
Cell adhesion molecules	1.3152	*0.6769*	1.1161	*0.8326*	**1.9713**	***0.0035***
Tolerance induction	1.1341	*0.8744*	1.3966	*0.6984*	**1.9658**	***0.0269***
Oxidative phosphorylation	NA	*NA*	1.1615	*0.7702*	**1.8406**	***0.0042***
Leukocyte migration	1.1451	*0.8242*	1.1283	*0.7551*	**1.7568**	***0.0160***
cAMP-mediated signaling	1.2633	*0.8117*	-1.3747	*0.6781*	**1.7469**	***0.0466***
Cell chemotaxis	-1.2244	*0.8117*	-1.4005	*0.3519*	**1.6538**	***0.0266***
T cell proliferation	-1.1409	*0.8327*	0.9728	*0.8683*	**1.6456**	***0.0287***
Focal adhesion-PI3K-AKT-MTOR signaling pathway	0.9251	*0.8684*	NA	*NA*	**1.5786**	***0.0076***
Hypoxia	0.9164	*0.8079*	1.1854	*0.5327*	**1.4939**	***0.0117***
Adipogenesis	1.2129	*0.3981*	-1.0692	*0.6584*	**1.3900**	***0.0269***
Glycolysis	NA	*NA*	0.9410	*0.7427*	**1.3533**	***0.0361***
MTORC1 signaling	-1.2514	*0.3413*	-0.9966	*0.9140*	**-1.2802**	***0.0349***
WNT-beta-catenin signaling	-1.0957	*0.5629*	1.0317	*0.7083*	**-1.5268**	***0.0349***
TNFa signaling via NF-κB	1.0688	*0.5601*	-1.3841	*0.2889*	**-1.6707**	***0.0014***
Signaling pathways regulating pluripotency of stem cells	-1.1068	*0.7972*	0.8970	*0.9521*	**-2.2534**	***0.0042***
Ribosome biogenesis in eukaryotes	-1.0595	*0.8349*	-1.6486	*0.1661*	**-2.7562**	***0.0042***
MYC targets (V2)	-0.8982	*0.8037*	-1.3864	*0.3433*	**-2.9127**	***0.0009***

Table S2. Distinctive phenotypes of genetically modified CD8 T cells

**Engineered CD8 T cells**	**CD8 donor mouse strain**	**Donor cell phenotype**		**Retroviral Vector**	**Distinctive phenotype of genetically modified CD8 T cell**
		**CD45 allele**	**CD90 allele**		
Foxp3UP	OT-1xCD45.1	CD45.1	CD90.2	MSCV-*Foxp3*-IRES-*Gfp*	$CD45.1^{+}GFP^{+}$
				MSCV-*Foxp3*-IRES-*Thy1.1*(#)	$CD45.1^{+}CD90.1^{+}$
	Pmel (*Thy1.1*)	CD45.2	CD90.1	MSCV-*Foxp3*-IRES-*Gfp*	$CD90.1^{+}GFP^{+}$
Mock	OT-1xCD45.1	CD45.1	CD90.2	MSCV-IRES-*Gfp*	$CD45.1^{+}GFP^{+}$
				MSCV-IRES-*Thy1.1*	$CD45.1^{+}CD90.1^{+}$
	Pmel (*Thy1.1*)	CD45.2	CD90.1	MSCV-IRES-*Gfp*	$CD90.1^{+}GFP^{+}$

(#) *Thy1.1* encodes CD90.1 protein.

SUPPLEMENTARY METHODS

Plasmids

pCL-Eco was a gift from Inder Verma[1] (Addgene plasmid # 12371). MSCV-IRES-*Gfp* was a gift from Tannishtha Reya (Addgene plasmid # 20672). MSCV-IRES-*Thy1.1* DEST[2] was a gift from Anjana Rao (Addgene plasmid # 17442). Mouse *Foxp3* open reading frame (CCDS29965.1) was synthesized (GenScript) and inserted into MSCV-IRES- *Gfp* and MSCV-IRES-*Thy1.1* plasmids.

Retrovirus production

PLATE cells (6×10^5 cells/well) were seeded 24 hours before transfection into 6-well plates in 2 mL/well of PLATE medium [DMEM-Glutamax, FCS 10%, sodium pyruvate 1%, essential amino acids 1%, HEPES, 100 U/mL penicillin (P) and 100 µg/mL streptomycin (S)] without P/S. The next day, cells (70% confluence) were transfected with a mixture containing plasmids and Lipofectamine 2000 (500 µL/well). The mixtures contained 3 µg/well of transgene-carrying plasmid, 2 µg/well of pCL-Eco (helper plasmid) and 10 µL/well of Lipofectamine 2000, and were prepared in OPTIMEM medium according to the manufacturer´s protocol. PLATE medium was changed 24 hours post transfection. The supernatant containing the retroviruses (RV) was collected 48 hours and 72 hours post transfection. Debris was removed by centrifugation at 2000 rpm for 1 minute. Supernatants were kept at 4ºC until T cell transduction.

Tissue processing

Tumor-bearing mice were sacrificed and excised tumors were incubated in a dissociation solution [RPMI-1640-glutamax supplemented with 400 U/mL collagenase D (Roche) and 50 µg/mL of DNase I (Roche)] for 30 minutes at 37ºC. Tumors were mechanically homogenized, filtered through a 70 µm nylon cell strainer (FALCON) and centrifuged at 2000 rpm for 8 minutes. After erythrocyte lysis with ammonium-chloride-potassium (ACK) lysing buffer (0.15 M NH_4Cl, 1 mM $KHCO_3$ and 0.1 mM Na_2EDTA), tumor cell suspension ($10^6\times$cells/ml) was overlaid on 3 mL of Lympholyte-M (Cederlane) and centrifuged at 1400 g for 20 min at room temperature. The interface was collected and washed in phosphate buffered saline (PBS) before mAb staining and flow sorting as detailed below. In some experiments, spleens and draining lymph nodes were also harvested, homogenized by mechanical dissociation through a 70µm nylon cell strainer and treated with ACK buffer to remove erythrocytes.

Cell labelling for FACS

Different fluorophore-conjugated mAbs or proteins were used for FACS and cell sorting: antibody against mouse CD90.1 (OX-7), GFP (FM264G), FOXP3 (FJK-16s), CD8 (53-6-7), CD45 (I3/2.3), CD45.1 (A20), CD45.2 (104), CD103 (2 E7), CD69 (HI.2F3), CD39 (Duha59), FASL (MFL3), IFNγ (XMG1,2), TNFα (MP6-XT22), GLUT1 (EPR3915), CD36 (HM36), Ki-67 (16A8), CD25 (3C7), CD62L (MEL-14), CD27 (LG.3A10), CD127 (A7R34), CD49a (HMa1), KLRG1 (14C2A07), CD43 (130 kd) (1B11), PD-1 (29F-1A12), TIM-3 (RMT3-23), LAG-3 (C9B7W), CCR3 (J073E5), CCR4 (2G12), CCR7 (4B12), CXCR3 (Cxcr3-173), CX3CR1 (Z8-50), human GZMB (GB11) and Annexin V. All these reagents were fluorochrome-labeled and obtained from Biolegend. Other reagents and dyes used were: 2-NBDG [2-(N-(7-Nitrobenz-2-oxa-1,3-diazol-4-yl)Amino)-2-Deoxyglucose] (Invitrogen), Bodipy-493 (Thermofisher), Bodipy-FL-C16 (Thermofisher), Zombie NIR Fixable dye (Biolegend), SYTOX Blue Dead Cell Stain (Thermofisher), 7-aminoactinomycin D (7AAD) (Thermofisher) and cell trace violet (CTV) (Invitrogen).

For analysis of surface markers, cells were incubated with Zombie NIR Fixable dye and subsequently with a mix of fluorophore-conjugated mAbs containing purified anti-CD16/32 (2.4G2) (Fc Block) mAb at RT (15 min). For tetramer staining, cells were first stained with APC-labeled H-2Kb/p15E(604-611)-tetramer (MBL) and, after washing, with fluorochrome-conjugated mAbs against mouse CD8 (KT15), CD45.1 and CD90.1 in the presence of Fc-Block. For analysis of chemokine receptors, cells were incubated with a mix containing the fluorophore-conjugated mAbs specific for the chemokine receptors and Fc Block at 37ºC (30 min), followed by mAbs specific for cell population markers at RT (15 min). For intracellular staining (CD36, GLUT1, granzyme B, Ki-67, IFNγ and TNFα) after incubation with Zombie NIR and fluorophore-conjugated mAb against surface markers, cells were fixed and permeabilized with Cytofix/cytoperm buffer (BD Biosciences) and then stained with the respective mAbs in the presence of purified Rat IgG. Anti-GFP mAb was added to the mix to counteract the loss of GFP intensity upon fixation. Before the intracellular detection of IFNγ and TNFα, cells were incubated with cognate peptides [OVA peptide SIINFEKL (1 µg/ml), Pmel peptide (EGSRNQDWL, 2µg/ml), p15E(604-611) peptide (KSPWFTTL, 1 µg/ml) (Neomps)], in the presence of Brefeldin A (diluted 1:1000) (Biolegend) for 4-6 hours. For intracellular staining of Foxp3, the FOXP3 Staining kit (eBiosciences) was used.

For CTV labeling, cells were resuspended in PBS at 10^6 cell/ml and mixed 1:1 (volume) with CTV (10 µM in PBS) (CTV final concentration 5µM). Cells were incubated 15 minutes in darkness at room temperature, after which 100µl of bovine serum per ml of sample were added for quenching. Lastly, cells were washed with PBS.

For staining with Annexin V, cells were surface stained with mAb as described above, washed in 1× Annexin Binding Buffer (Biolegend) and stained with PE-conjugated Annexin V and 7AAD in 1× Annexin Binding Buffer.

For glucose uptake, cells ($1\text{-}5\times10^5$) were cultured in glucose-free RPMI medium for 60 minutes at 37ºC. Then, the glucose analog 2-NBDG was added (20 µM final concentration) and cells were cultured for an additional 30 minutes. Cells were surface stained with mAbs and labeled with 7AAD before FACS analysis.

For FA uptake, cells ($1\text{-}5\times10^5$) were cultured in PBS supplemented with FA-free bovine serum albumin (FA free-BSA) (20µM) for 60 minutes at 37ºC in an incubator. Bodipy-FL C16, the analog of palmitic acid, was added to the cells (1µM final concentration) and they were cultured for an additional 30 minutes. Surface staining with mAbs and 7AAD labeling were performed before FACS analysis.

For lipid droplet content, cells ($1\text{-}5\times10^5$) were surface stained with mAbs and resuspended in PBS-FA free-BSA (20 µM). Bodipy-493 was added (1 µg/ml final concentration) and cells were cultured for 60 minutes at 4ºC. 7AAD live/dead staining was performed before FACS analysis.

Chemotaxis assay

CD8 T cell migration was evaluated using 24-well, transwell plates with 5 µm pore size (Costar). Genetically modified CD8 T cells were washed and adjusted to 10^6 cells/ml in complete medium. One hundred µl of cell suspension were placed in the top chamber of the transwell. CCL22 (Inmunotool) prepared at the indicated concentration in culture medium (500 µl per well) was placed in the bottom chamber of the transwell. After 3 hours of incubation at 37ºC, the top chamber was removed and the number of cells in the bottom chamber was counted by FACS using a Cytoflex volumetric cytometer (Beckman-Coulter).

Suppression assay

$CD8^+$ splenocytes (2×10^4/well) isolated from BL6 ($CD45.2^+$) mice and labeled with CTV were activated (96-well polystyrene round-bottom microwell plates, Nunc) with anti-CD3/CD28 mAb-coated beads (2:1, bead:CD45.2 cell ratio) in the absence of IL-2. Activation occurred in co-culture with in vitro pre-activated (7 days) Foxp3UP or Mock OT-I ($CD45.1^+$) T cells, or with Foxp3UP or Mock OT-I ($CD45.1^+$) cells isolated from tumors 7 days after ACT. at different $CD45.1^+$:$CD45.2^+$ ratios. After 72 hours, cells were stained with Zombie NIR and anti-CD45.2 mAb and proliferation of $CD45.2^+$ CD8 cells was determined by CTV dilution and FACS analysis. As positive control, CD4 Treg ($CD4^+CD25^+$) cells were used. Murine $CD4^+CD25^+$ cells were isolated from the spleen of BL6 ($CD45.2^+$) mice using the $CD4^+CD25^+$ Regulatory T Cell Isolation Kit (Miltenyi) following the manufacture's recommendations.

Activation-induced cell death (AICD) Assay

Genetically modified CD8 T cells expanded i*n vitro* for 4 days were cleaned form dead cells using the Dead Cell Removal Kit (Miltenyi) following the manufacturer's instructions. Cells were cultured (10^5/well, 200 µl) in 96-well plates coated with or without anti-CD3 (1 µg/ml) in the absence of IL-2. After 24 hours of culture, samples were stained with anti-CD90.1 and anti-FASL mAbs, Annexin V and 7AAD and the surface expression of FASL and viability of transduced cells was analyzed by FACS.

IL-2 cytokine withdrawal-induced cell death (CWID) assay

Genetically modified CD8 T cells expanded *in vitro* for 8 days were cleaned from dead cells using the Dead Cell Removal Kit (Miltenyi). Cells were cultured (5×10^5/well, 1000 µl) in 48-well plates in complete medium without IL-2 for 72 hours, after which they were harvested and stained with Annexin V, 7AAD dye and anti-CD90.1 mAb. The viability of transduced cells was analyzed by FACS.

***Ova* and *Thy1.1* gene expression analysis**

RNA isolated from tumors (Maxwell 16 LEV simplyRNA tissue kit, Promega) (1 µg/sample) was treated with DNase I (Invitrogen) prior to RT with Moloney murine leukemia virus reverse transcriptase (Invitrogen) in the presence of RNaseOUT (Invitrogen) and 1/4 of cDNA preparation was used for each PCR. QPCR was performed with 200 nM of validated primers in 25 µl reactions using the Platinum SYBR Green One-Step qPCR kit (Invitrogen) and the following program: 50°C for 2 min, 95°C for 10 min, and 40 cycles of amplification at 95°C for 15 s and 62°C for 60 s. Relative levels of target mRNA were compared with *Gapdh* using the $2^{\Delta\Delta Ct}$ method, where the control cell type or treatment group was normalized to 1. Primers were obtained from Integrated DNA Technologies. *Ova*: (F) 5′-CACAAGCAATGCCTTTCAGA-3′; and (R) 5′-GAATGGATGGTCAGCCCTAA-3′; *Gapdh*: (F) 5′-AACTTTGGCATTGTGGAAGG-3′ and (R) 5′-ACACATTGGGGGTAGGAACA-3′; and *Thy1.1*: (F): GCCAACTTCACCACCAAGGATG and (R) GATGTGTTCTGAACCAGCAGGC.

Statistical analysis

The standard deviation (SD) was used to estimate the dispersion of individual observations over the mean, while SEM was used to estimate the variability of the means of different groups[3]. We used SD for the results obtained in an analytical assay, in which (for each experimental condition being compared) there were triplicate to quintuplicate analytical determinations from one representative experiment. We used SEM for the results obtained in a biological experiment, in which (for each

group being compared) there were data from different animals, or when (for each group being compared) compiled data from different experiments were available. For simple comparisons, unpaired or paired two-tailed Student *t* tests were used. Multiple comparisons were performed by two-way ANOVA followed by the Bonferroni multiple comparisons test. Tumor growth curves were compared using non-linear fit test. The FDR calculation was made using the Benjamini-Hochberg method. Differential survival was assessed using the Mantel-Cox test. Significance was set at $p < 0.05$.

REFERENCES OF SUPPLEMENTARY DATA

1. Naviaux, R.K., Costanzi, E., Haas, M. and Verma, I.M. (1996). The pCL vector system: rapid production of helper-free, high-titer, recombinant retroviruses. J. Virol. 70, 5701–5705.
2. Wu, Y., Borde. M., Heissmeyer, V., Feuerer, M., Lapan, A.D., Stroud, J.C. et al. (2006). FOXP3 Controls Regulatory T Cell Function through Cooperation with NFAT. Cell 126, 375–387.
3. Barde, P. and Barde, M. (2012). What to use to express the variability of data: Standard deviation or standard error of mean? Perspect. Clin. Res. 3, 113-116.
